# The Future Is Not Bright: Evaluation of Rat Preferences for Color and Intensity of Light

**DOI:** 10.3390/ani14142045

**Published:** 2024-07-12

**Authors:** Melissa Swan, Aidan Horvath, Rebecca K. Pritchett, Amanda J. Barabas, Debra Hickman, Brianna N. Gaskill

**Affiliations:** 1 College of Veterinary Medicine, Purdue University, West Lafayette, IN 47907, USA; 2 Animal Sciences Department, Purdue University, West Lafayette, IN 47906, USAajb201@case.edu (A.J.B.); 3 Laboratory Animal Resource Center, Indiana University, Indianapolis, IN 46202, USA; dhickman@aaalac.org

**Keywords:** rat, light, welfare, preference, refinement

## Abstract

**Simple Summary:**

Light is vital to many aspects of normal physiological functioning and behavior; however, the ideal lighting conditions for laboratory rats is unknown. The aim of this study was to determine rats’ preferences for the spectral make-up of light created by red or clear cages at high and low light levels (25 or 200 lux). Rats’ preferences were tested three times with a free choice test as they matured. Most rats preferred dim light to bright light, no matter the color. But some rats from clear and bright cages preferred clear light more; however, this did change with experience. Furthermore, the rats’ sex, type, and behavior mattered for their choices. This study concludes that darker environments are more likely to improve rat welfare by providing a condition they prefer, but the spectral make-up of the light created by red-tinted cages was not aversive.

**Abstract:**

Light is a key factor influencing the welfare of laboratory rodents, but little is known about their optimal lighting condition. It i common knowledge that rats prefer dim light, so bright light is mitigated with red-tinted shelters or cages, which alter both the color and intensity of light. Because both aspects are altered, the contribution of each feature to rodent preference is unknown. Further, it is unknown if this preference is influenced by previous experience. We hypothesized that rats would prefer lower light intensity and that their preferences would be influenced by their housing environment. Breeder pairs of rats were randomly separated into four treatments groups: red 200 lux, red 25 lux, clear 200 lux, and clear 25 lux. The breeders’ offspring were tested three times in an apparatus that offered access to each environment, and their preferences were analyzed. Generally, the rats preferred the lower-lux environments and showed no color preference. However, the rats from the clear, 200 lux cages, preferred clear caging and only showed a preference for 25 lux conditions during the second and third preference tests. These results suggest that the light intensity, more than color, should be considered when designing rodent housing and testing facilities.

## 1. Introduction

Animal welfare is an essential part of performing ethical and high-quality scientific studies. Fraser et al. outlines three concepts of animal welfare: functioning, defined as the preservation of animals’ good health and normal development; natural living or replicating natural environments that promote natural behaviors; and positive affect, or the mental wellbeing of animals, characterized by the lack of fear, distress, and pain and the ability to experience normal pleasures [1]. Light is a well-documented mediator of many aspects of rodent wellbeing, through its effects on circadian rhythm, hormone regulation, sleep, cognition, reproduction, growth, maturation, social interactions, cancer incidence, and tumor growth [2,3,4,5]. In particular, the intensity of light has been shown to induce stress and anxiety at levels as low as 60 lux and retinal deterioration of albino animals at levels as low as 80 lux [6,7,8]. Light is also thought to play a role in the mental health of rodents through its impacts on the hypothalamic–pituitary–adrenal (HPA) axis and the expression of corticosterone, a hormone that has been associated with stress and depression [9]. Lighting conditions inconsistent with natural preferences seemed to increase overall levels of corticosterone in rodents and increased depressive-like behavior [10], and rodents exposed to 30-h blue light pulses showed significantly higher levels of blood corticosterone compared to violet, green, and dark conditions [11]. Different lighting conditions have also been shown to impact circadian rhythms and aspects of homeostasis in rodents. Rodents housed in red cages showed an earlier onset of daily activity compared to rodents housed in regular caging [12]. Low-contrast light/dark cycles and constant low-level lighting environments both affect the daily rhythm of rodent locomotor activity [13]. Furthermore, the use of different-colored caging (red, yellow, and blue) had different effects on rodent physiology, with red caging in particular decreasing the circulating insulin, glucocorticoid, and glucose values [14].

Given these impacts of light on rodent physiology, it is unknown what lighting conditions may be preferred by laboratory rats. Understanding what those preferences are is more likely to improve welfare and reduce stress. However, there is very little in the literature defining what those preferences may be. One study found that rats were less willing to explore mazes lit with white light compared to red light [15], but they preferred white over red painted cages [16]. Despite this preference for white caging, it is commonly accepted that rodents prefer red-tinted environments. In recent years, caging manufacturers have begun selling red-tinted cages and shelters based on the notion that mice cannot see the color red in the visual spectrum due to their lack of red-sensitive photoreceptors [17,18]. It is worth noting that, recently, mice have been found to detect red light at high intensities [2]. While color is an important factor, only a single study has evaluated what intensities provoke avoidance in rats, and those thresholds depend on coat color and eye pigmentation. Pigmented animals found >65 lux to be aversive, while albino animals were more sensitive and avoided intensities above 25 lux [19]. Piecing together all this data to define preferences for the common laboratory rat is difficult since none of these studies controlled for both the spectral make-up of light and its intensity in the same study. For instance, when light moves through red-tinted cages, the color make-up and intensity are both altered. Thus, it is difficult to know which aspects of light (intensity vs. color) rats prefer or find aversive.

The lighting environment within laboratory rodent cages can be affected by many factors, including the clarity of the cage wall or the presence of tinted thermoplastic shelters. These factors induce variation in both the wavelength and intensity of light, which, as previously stated, could both impact rodent preference. Additionally, it is not clear how much of the observed preference is innate or due to familiarity to a particular lighting environment, a phenomenon occasionally seen in animal preference research [20]. To the best of our knowledge, no previous studies have attempted to parse the influence of light color vs. intensity on preference, nor investigated the contribution of familiarity or novelty on light preference. Therefore, this study has two primary aims. The first aim of this study is to investigate whether rodent lighting preferences are influenced by either the color or intensity of the lighting. Our initial hypothesis was that the light intensity drives rat preferences over the color make-up of the light. The second aim of our study is to determine whether home cage lighting environments influence lighting preference over time. Specifically, we hypothesized that rodents will initially prefer lighting conditions similar to those of the cage in which they were raised, but this may change over time and with experience.

## 2. Materials and Methods

The animal work for this study was conducted at Purdue University, in West Lafayette, Indiana, USA, in a facility accredited by the Association for Assessment and Accreditation of Laboratory Animal Care (AAALAC), under the approval of the Purdue Institutional Animal Care and Use Committee (protocol 1504001229).

### 2.1. Animals and General Housing

Prior to the start of this study, the rats were tickled to prepare them for frequent handling. During this time, data were collected for another study to understand the rats were affected under these different lighting conditions [21]. The specific methods for housing these animals can be found in that manuscript, but we have summarized the methods below. 

Breeding pairs for this study were purchased from Charles River (Raleigh, NC, USA) at 8 weeks old. To generalize across different types of rats, both pigmented (Crl: LE, Long–Evans; LE) and albino (Crl: CD; CD) rats. Albino animals lack melanin pigment in the iris and are, therefore, more susceptible to lesions [3] and more likely to avoid light intensities above 25 lux than pigmented rats (albino: HSD/CPB/WU; pigmented: HSD/CPB/S3) [19].

Rats are dichromats, with two types of cones that provide color vision. One peaks at approximately 510 nm (green color; [22]) and the other at about 358, which provides vision in the UV spectrum [23]. Only small differences in these peak sensitivities have been identified [24,25]. In one study, with albino rats (type of rat not reported) showed a preference for green and blue colors [26], while another showed that pigmented rats (Long–Evans) could successfully discriminate (80% correct) between 370 nm and a test light when wavelengths were above 420 nm [25]. When it comes to the ability to see the color red, rat cones and non-image-forming intrinsically photosensitive retinal ganglion cells are not responsive to light above 620 nm [27,28]. However, activation may also depend on the intensity of the light, not just the specific wavelength. It is now believed that rats are not red-light blind [29,30,31], and rat retinas respond to far-red light with great sensitivity [32]. A study conducted by Niklaus et al. found that pigmented Brown Norway rats exposed to photopic bright red light (656 nm at approximately 92 lux) resulted in a larger response than the albino Wistar rats [32]. 

The animals used were free of a vendor-selected set of diseases: http://www.criver.com/products-services/basic-research/health-reports, accessed on 4 July 2024. After shipment, the rats were randomly paired into eight monogamous breeding cages of the same stock using a random sequence generator generated by random.org. See Section 2.2 for the specific breeding procedures.

The rats were housed in either a clear or red polycarbonate cage with a stainless steel wire bar lid (22″ L × 12-1/2″ W × 8″ H; Lab Products, Inc., Seaford, DE, USA, Rat/Hamster Cage 18730 or 18730M with lid 20311). The red cages were custom-made to match the tint of commonly used red intra-cage shelters (e.g., Bio Serv, Rat RetreatTM, Flemington, NJ, USA). Aspen bedding (300 g, Envigo Teklad, Madison, WI, USA, 7093), 20 g of nesting material (Crink-l’nestTM material Anderson Inc., Maumee, OH, USA), and a kiln-dried hardwood block (Bio-Serv, Flemington, NJ, USA, K3512) were provided as bedding and nesting material. An ad libitum rodent diet (Envigo Teklad, 2018, Madison, WI, USA) and reverse osmosis water were provided to all cages. To reduce clouding and preserve consistent intra-cage light conditions, the cages were washed every two weeks. On alternative weeks, when the cage bottoms were not replaced, clean bedding and nesting material were provided. 

#### Detailed Housing Procedures

**Figure 1:**
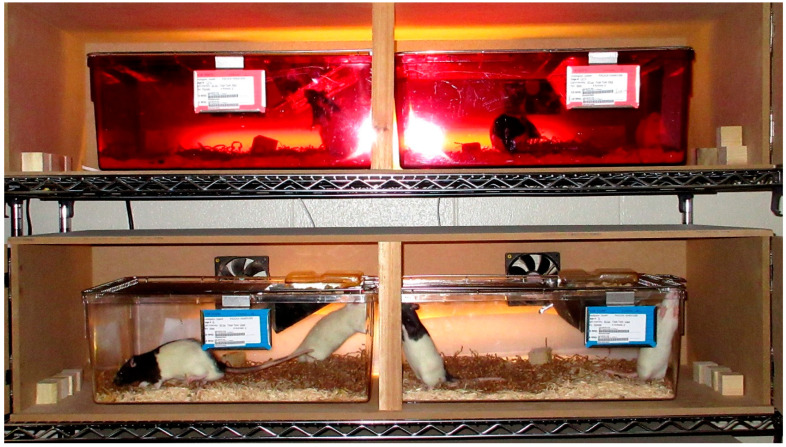
Two of sixteen light box assemblies (housing two cages per box) used to create the different home cage lighting conditions. Depicted is an example of the red home cage color condition on top and the clear home cage condition on bottom.

Different lighting conditions were created via the use of 16 wooden light boxes (made of medium-density fiberboard), in which the rats’ home cages could be placed. In total, four wire racks were assembled to hold the light boxes, creating unique lighting environments of 200 or 25 lux. A light box (which contained 2 rat cages) was randomly assigned to one of the following treatment groups (red 200 lux, red 25 lux, clear 200 lux, or clear 25 lux; Figure 1). The red cages were investigated because manufacturers have started to produce red cages that are meant to mimic the tint of commonly used red shelters, which rats are thought to prefer. The light intensity levels were chosen based on the average light measurement within the front of a cage housed on the middle of a rack (in-house evaluation; 200 lux) and the low lighting conditions that a red shelter provides. This is also similar to the threshold for albino avoidance (25 lux). We were prevented from placing both red and clear cages in the same box, as red-tinted plastic significantly reduces light intensity. During breeding, each light box held a cage of CD and a cage of LE rats. At weaning, a CD and LE rat pup were paired into a single cage and each lighting box contained a cage of each sex. Figure 1 depicts an example of this setup.

Again, refer to reference [21] for specifics. The light intensity levels were maintained with light fixtures (premium direct wire, 36-inch fluorescent; General Electric, Boston, MA, USA, 10142) and multi-layer filters (photography-grade diffusion paper; LEE Filters, Burbank, CA, USA, 129 Heavy Frost) attached to the center of the box at the cage level. The light intensity was monitored when the animals were first housed, and twice a week afterward, using a dual-range light meter (Traceable^®^ Products, Webster, TX, USA, 3251CC; measuring the photopic lux). Light measurements were taken at the center of the cage. The light intensity ranges were 193.47 ± 15.16 and 25.90 lux ± 3.99 lux. Lights in the housing boxes were on a shifted 12:12 h light/dark cycle (lights on at 12:00 a.m.). The main housing room lights were kept off. In order to perform basic tasks within the main room, a light, with similar intensity to what was in the rat’s housing box, was placed on a table.

The temperature within the box was maintained at 24 ± 2 °C, and two fans (Cooler Master, Zhonghe Dist., New Taipei City, Taiwan, Silent Fan 120 SI2) were installed at the back of the light box to provide air circulation. HOBO^®^ data loggers were used to monitor the temperature levels throughout the experiment.

### 2.2. Breeding Procedures

Ideally, we would have synchronized the breeding and births of the rat pups for each intensity and cage color treatment and stock. Unfortunately, we were unable to synchronize the breeding across all treatments because the clear 200 lux breeding pairs of both stock did not produce litters initially. In order to produce the pups needed, the lighting intensity was lowered to 60 lux and gradually stepped up to 200 lux during the week before birth. Therefore, no single month of testing contained all treatment combinations. As a result, months 1–2 contained the 25 lux treatments of both colors, month 4 tested the clear 200 lux, and month 5 tested the red 200 lux. 

To reduce the overall number of offspring for the project, sires were removed from the breeding cages and singly housed in a different box of the same lighting conditions once the female breeders were visibly pregnant. The dams were allowed to rear their pups until weaning at 21 days ± 2 days. All rats continued to receive the same enrichment (nesting and wood block) regardless of housing arrangement. Randomly chosen offspring (using the random number generator at Random.org) were weaned into same sex pairs (1 CD and 1 LE) of the caging and lighting treatments corresponding to their rearing conditions. 

### 2.3. Experimental Treatments

A 2 × 2 factorial design was used to compare the preferences of the rats for cage color (red or clear) and light intensity (25 or 200 lux). We calculated an a priori sample size using Mead’s resource equation (Mead, 1990). Six cages were used in each of the 4 possible combinations of cage color and light intensity, providing an overall total N of 24 cages. While the treatment was applied to the cage, behaviors could be further separated into differences between the two types of rats. Thus, the experimental unit was the rat (48 rats) with repeated measures per cage. The rats were bred in-house using the procedures outlined above in Section 2.2. 

### 2.4. Preference Testing Procedures

**Figure 2:**
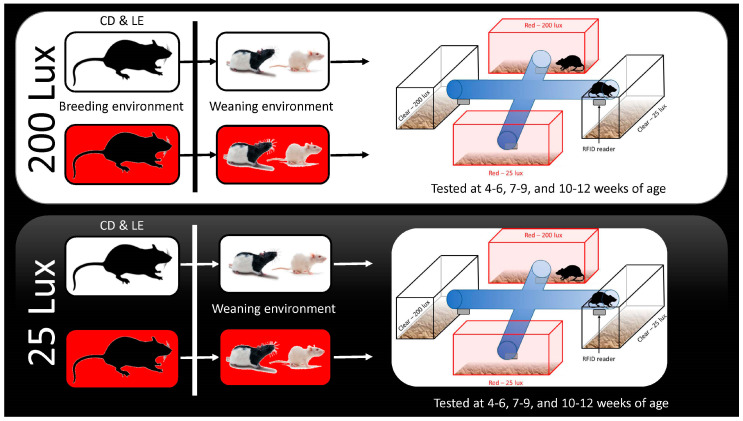
Full experimental procedure diagram depicting the environments in which each group was bred, weaned, and tested and the timeline of procedures.

To assess the rats’ preferences for the different lighting conditions, we used a preference apparatus consisting of one light box of each lighting environment (red 200 lux, red 25 lux, clear 200 lux and clear 25 lux) connected by clear tubing. The rats were allowed to acclimate to each environment and the connecting tubing for 12 h (6 h light, 6 h dark) before having unrestricted access to all four environments for 3 consecutive days. Preference testing was conducted 3 times during each of the critical developmental periods, including the juvenile (4–6 weeks), puberty (7–9 weeks), and adulthood (10–12 weeks) stages. Videos of the test sessions and rat location were scored using instantaneous scan sampling every 15 min. The behaviors were categorized using a standard ethogram: Active behavior included all locomotive behavior, including rearing, grooming, eating, nesting, or manipulating the wood chew block; essentially, if the animal was moving around the preference apparatus, they were considered active. Inactive behavior meant the animal was motionless, and either lying curled up on its side, or curled up with its face tucked into its body and out of sight of the camera, occasionally interrupted by brief single twitches of the body. The animal may also have been sitting still or curled up with the face lifted. Unknown behavior meant the animal was out of sight or the behavior could not be determined. Video observers were blind to the cage’s original housing color and intensity conditions but not blind to the type of rat, since that could be visually determined. They were, however, blind to the hypothesis about which environment albino and pigmented rats might choose. No data were excluded from the analysis. The testing sessions were run over three days, and the data from the light periods and dark periods were summarized separately (behavior from the 3 light periods = 100% of the light budget, and behavior from the 3 dark periods = 100% of the dark budget). Once the data were summarized, any occurrences of rats being observed in the tube or doing unknown behaviors were excluded from the final dataset to assure independence of the data. Thus, the light and dark time budgets did not equal 100%. To further validate the location preference, 20 g of nesting material was provided within each testing chamber, in which the rats were able to freely move. At the end of each preference test, the amount of material in each location was weighed to determine the nest movement. The full breeding and testing procedure is summarized in Figure 2.

### 2.5. Spectral Data

At the end of the study, the specific and more nuanced lighting environment was measured for each condition using a spectrometer (Ocean Optics Jaz, Winter Park, FL, USA) [21]. The data are reported as wavelengths (nm). The measurements were taken at the center of each cage and averaged across 10 measurements (Figure S1).

### 2.6. Statistical Analysis

All data were analyzed in JMP statistical software (JMP^®^ 16.1; SAS Institute Inc., Cary, NC, USA) using general linear mixed models (see Table S1 for the full statistical models and output). Assumptions of the general linear mixed models were confirmed via visualization, and logit transformations were used. For the nesting material analysis, a square root transformation was used. For all tests, the level of statistical significance was set at *p* < 0.05. The relationships between different factors were evaluated using three-way interactions, which were interpreted using test slices and subsequent Bonferroni corrected custom contrasts or Tukey corrections for multiple comparisons. The results from the statistical models are presented as the least square mean ± standard error of the mean (LSM ± SEM). Any data that were transformed for analysis are presented in text with back-transformed LSM values and SEM values estimated via the Delta Method.

To improve the readability of our results section, we have omitted the test statistic details from the written section but have provided the full model information in Table S1. For the in-text stat reporting, the exact *p*-values are reported unless they were smaller than 0.001. However, the results of the post hoc Tukey tests are reported as > or <0.05. Table S2 provides the analysis script in SAS and the data for transparency. 

## 3. Results

### 3.1. Effects of Housing Conditions on Preferred Location

The effects of the housing conditions on location preference were evaluated through post hoc examination of statistical interactions, interpreted as differences in the percentage of time the animals spent in a given testing environment depending on their original housing condition. In this context, the home cage color and home cage intensity refer to the lighting conditions in which animals were born and raised. The test color and test intensity refer to the chamber of the testing apparatus in which the animal was observed. 

**Figure 3:**
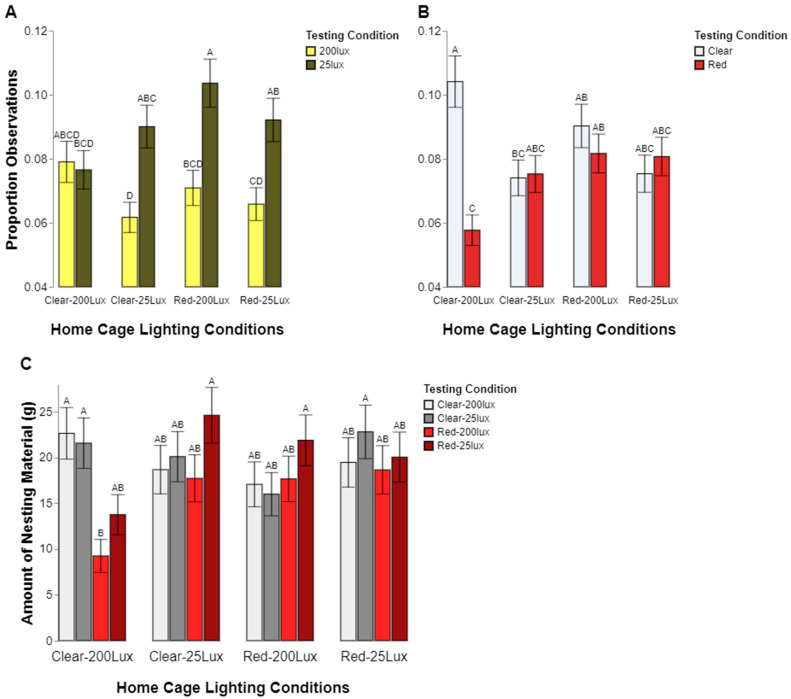
Proportions of observations where a rat was seen to be present in the test lighting conditions for the level of intensity, with light yellow representing 200 lux and dark yellow representing 25 lux (**A**), color, with white representing the clear test condition and red representing the red test condition (**B**), and the amount of nesting material measured in each test location, with white representing the clear/200 lux location, grey representing the clear/25 lux location, light red representing the red/200 lux location, and dark red representing the red/25 lux location (**C**). In each graph, the data are presented separately for each of the four possible home cage lighting conditions. Different letters indicate statistically significant differences.

Two significant three-way interactions existed (1) among the home cage color, home Cage intensity and intensity preference (*p* = 0.0017; Figure 3A) and (2) the home cage color, home cage intensity, and color preference (*p* = 0.0026; Figure 3B) indicating that the variability in preference differed depending on the animal’s original home cage conditions. Almost all housing groups showed a consistent preference for the 25 lux location within the preference apparatus (all *p* values < 0.05), except the clear/200 lux housing condition, which showed no significant difference in the percentage of time spent in the 200 lux vs. 25 lux conditions (*p* = 1.0000). There was also an interesting pattern in color preference. Rats from the clear/200 lux home cage condition were observed more often in the clear test chambers vs. the red (*p* < 0.05). Rats from the clear/25 lux, red/200 lux, and red/25 lux home cages showed no significant difference in the percentage of time spent in the clear vs. red testing chambers (*p* = 1.0000, *p* = 0.9746, *p* = 0.9976 respectively). Taken together, these results suggest that the rats generally preferred the lower light intensity conditions, with little preference for color, except for the animals raised in clear and bright housing.

Nesting material movement also provided insight into where the rats preferred to build their nest and potentially rest. While a simpler model was used to analyze the nesting movement, a similar pattern of the observed locations emerged. We found a significant three-way interaction among the home cage color, home cage intensity, and test location (*p* = 0.0074; Figure 3C). Similar to the previous analysis of the observed locations, the rats from the clear/200 lux home cages generally moved more nesting material into the clear testing chambers compared to the red, but only for the more intense, red/200 lux condition (clear/200 lux vs. red/200 lux, *p* = 0.0056; clear/25 lux vs. red/200 lux, *p* = 0.0150; clear/200 lux vs. red/25 lux, *p* = 0.4697; clear/25 lux vs. red/25 lux, *p* = 0.6710). No significant differences in the amount of nesting material in each chamber were observed for the rats within any of the other home cage lighting conditions.

### 3.2. Other Influences on Testing Preference

**Figure 4:**
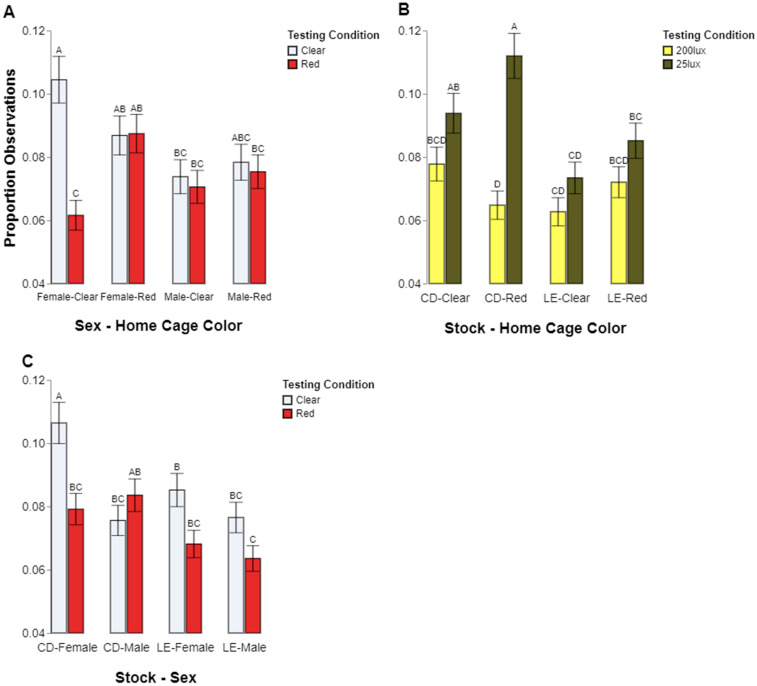
Proportions of observations where a rat was seen to be present in the given test color condition, with white representing the clear test condition and red representing the red test condition for each combination of sex and home cage color (**A**). Proportions of observations where a rat was seen to be present in the given test intensity condition, with light yellow representing 200 lux and dark yellow representing 25 lux for each combination of stock and home cage color (**B**). Proportions of observations where a rat was seen to be present in the given test color condition, with white representing the clear test condition and red representing the red test condition for each combination of stock and sex (**C**). Different letters indicate statistically significant differences.

There were significant interactions among sex, home cage color, and color preference on where the rats spent more time (*p* = 0.0003; Figure 4A). The post hoc tests revealed that the female rats raised in clear caging spent significantly more time in the clear testing chambers compared to the red (*p* < 0.05), while the females from red home cages and males from any other housing condition all showed no significant difference in color preference (female/red, *p* = 1.0000; male/clear, *p* = 0.9999; male/red, *p* = 0.9999). 

There were also significant interactions among stock, home cage color, and intensity preference (*p* = 0.0152; Figure 4B). The post hoc analysis showed that the CD rats from the red home cages spent a significantly higher percentage of time in the 25 lux testing chambers compared to the 200 lux chambers (CD-Red, *p* < 0.05), but this comparison was not significant for any other combinations of stock and home cage color (CD/clear, *p* = 0.4070; LE/clear, *p* = 0.6669; LE/red, *p* = 0.5480). 

Finally, there were significant interactions among the stock, sex, and color preference (*p* = 0.0125; Figure 4C). The post hoc tests revealed that the CD female rats spent a significantly higher percentage of time in the clear test chamber vs. the red test chamber (*p* < 0.05) and a greater percentage of time in the clear test chamber compared to all other groups (CD males, *p* < 0.05, LE males, *p* < 0.05; LE females, *p* < 0.05).

### 3.3. Effects on Behavior

In addition to where the rats spent their time (location in the preference apparatus), we were also interested in what they were doing in these environments and if they were using the different environments in different ways. For these analyses, interactions were interpreted for differences in the percentage of time spent in an active or inactive state depending on other factors. As such, for these analyses, the color/intensity preference acted as factors contributing to the observed differences in the same way as the home cage color/intensity. When they appear in the analyses, the home cage color/intensity refers to the effect of the animals’ habituated lighting conditions from their home cages, and the color/intensity testing chamber refers to the effect of the animals’ immediate environment, that is, the conditions in which they were observed at the time of activity evaluation. 

**Figure 5:**
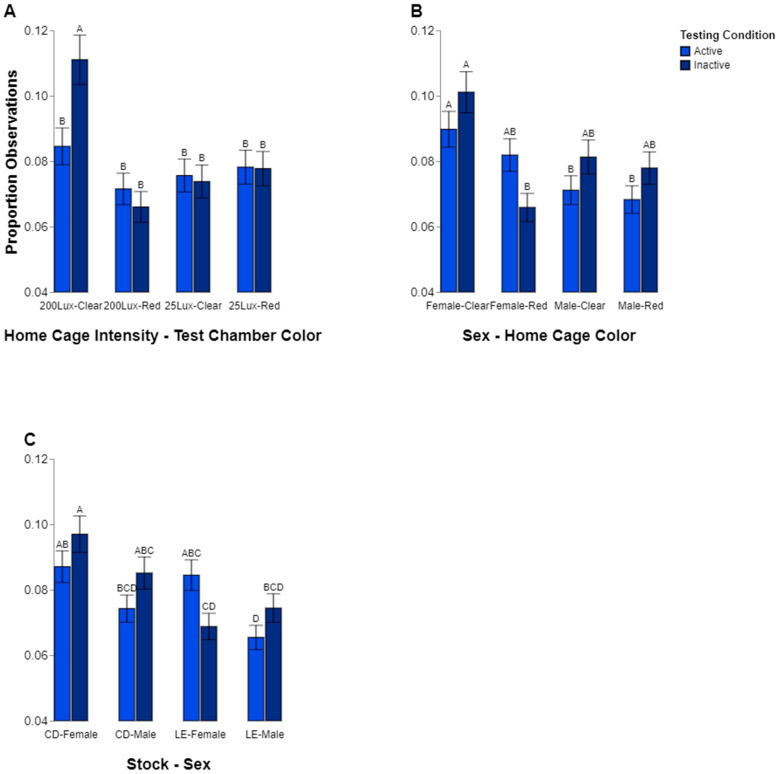
Proportions of observations where a rat was seen to be either active or inactive in the test chamber, with light blue representing active and dark blue representing inactive. The results are displayed separately for each combination of home cage intensity and test chamber color (**A**), each combination of sex and home cage color (**B**), and each combination of stock and sex (**C**). Different letters indicate statistically significant differences.

We found a three-way interaction among home cage intensity, color preference, and behavior (*p* = 0.0095; Figure 5A). The rats from the 200 lux home cages, when observed in the clear test chamber, spent significantly more time inactive compared to active (*p* < 0.05). When looking only at inactive behavior, and comparing it within home cage intensities, the rats from the 200 lux home cages spent significantly more time inactive when in the clear test chamber compared to the red test chamber (*p* < 0.05). However, inactivity did not differ between the rats housed in the 25 lux home cages (clear/25 lux vs. red/25 lux, *p* = 0.9991). When comparing the 200 lux and 25 lux housing conditions within the same-colored test chamber (clear/200 lux vs. clear/25 lux), the rats raised in the 200 lux home cages spent significantly more time inactive compared to the rats raised in the 25 lux conditions (*p* < 0.05). When observing the red testing chambers, no differences existed in the time spent inactive between the 200 lux and 25 lux rats (*p* = 0.6214). No other differences between active and inactive behavior were observed for any of the red/200 lux (*p* = 0.9593), clear/25 lux (*p* = 1.0000), or red/25 lux (*p* = 1.0000) combinations.

The behavior in each testing chamber was also influenced by a three-way interaction among sex, color preference, and behavior (*p* = 0.0198; Figure 5B). The female rats spent more time inactive inside the clear test chambers compared to the red test chambers (*p* < 0.05). No differences between the test chambers were present in the females’ active behavior (*p* = 0.9632), nor were there any differences observed among the males for these comparisons (inactive, red vs. clear, *p* = 0.9997; active, red vs. clear, *p* = 0.9997). When comparing between the sexes, we found that in the clear testing chamber, the females spent more time active compared to the males (*p* < 0.05), but there was no sex difference in the time spent active within the red test chambers (*p* = 0.1095). When looking at the sex differences in the time spent inactive, there were no differences observed in the clear or red testing chambers (*p* values all >0.05). When comparing the time spent active to the time spent inactive, no differences among the sex/chamber color groupings were observed.

Finally, sex and stock seemed to have an effect on behavior given the interaction between these factors (*p* = 0.0205; Figure 5C). Differences were only observed between the stocks of female rats. The CD female rats were observed to be inactive more often compared to the LE female rats (*p* < 0.05). When looking at the time spent in an active state, there were no differences observed between the stocks of females (*p* = 0.9999). For the males, no differences were observed between the stocks for either the active (*p* = 0.6391) or inactive state (*p* = 0.6107). When looking at sex differences within the stocks, we found that the LE female rats spent more time active than the LE males (*p* < 0.05), but no sex differences were found between the CD rats (*p* = 0.3045). No other differences among the groups were found. 

### 3.4. Effects over Time 

**Figure 6:**
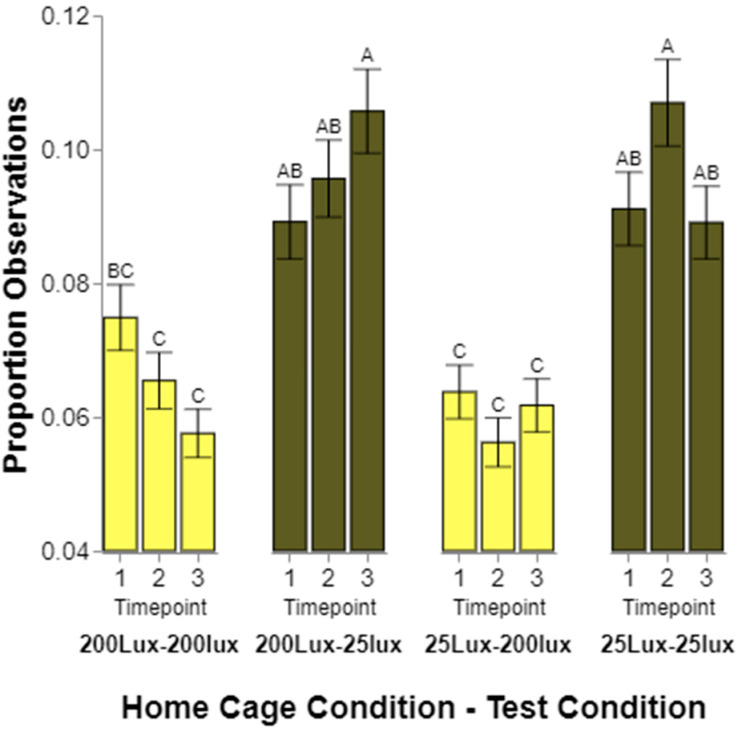
Proportions of observations where a rat was seen to be in either the 25 lux (dark yellow) or 200 lux (light yellow) test intensity condition over three observation time points for rats from both of the 25 lux and 200 lux home cage conditions. Different letters indicate statistically significant differences.

The rats’ preferences were tested three times as the rats aged. It is possible that rodents’ preferences for cage lighting change based on their exposure to and experience with other lighting conditions. We hypothesized that the rats would initially prefer lighting conditions similar to those of the home cages in which they were raised but would change over time. We looked for any alterations over time by analyzing three-way interactions among the time points, housing lighting, and lighting preference. When looking at color preferences, we did not find a significant three-way interaction among the time points, home cage color, and test color (*p* = 0.3872), indicating a lack of evidence for their color preference changing over time. There was a significant interaction among the time points, home cage intensity, and test intensity (*p* = 0.0087; Figure 6). In general, the rats spent more time in the 25 lux testing locations than the 200 lux. However, the rats originally raised and housed in the 200 lux environments were equally observed in the 200 and 25 lux testing conditions when tested for the first time. When tested later (time points 2 and 3), the time spent in 25 lux was more than in 200 lux. When looking only at animals that were born and raised in 25 lux housing, no patterns emerge, except that they were observed in 25 lux conditions more often than 200 lux. 

## 4. Discussion

Lighting conditions can impact animal physiology and welfare, but little is known about what rats actually prefer. The primary aim of this study was to investigate whether rodent lighting preferences are influenced by either the color or intensity of the lighting. Specifically, we expected light intensity to drive rat preferences over the color make-up of light. In general, most animals preferred lower light (25 lux) locations in our preference apparatus, which was consistent across both the albino and pigmented rats. While the rats housed in clear/200 lux appeared to choose differently than the rest, there was evidence of their preference shifting to 25 lux with repeated exposure and experience. This finding is consistent with citation [6]’s result suggesting that rodents were more averse to higher light levels. 

This study not only provides further evidence for laboratory rats in a home cage preferring low lighting conditions but also what conditions they should be exposed to generally. The guides for the care and use of laboratory animals do not specify any engineering standards for lighting environment, but many reference the statement that “…325 lux (30-ft candles) approximately 1 m (3.3 ft) above the floor appear to be sufficient for animal care and do not cause clinical signs of phototoxic retinopathy in albino rats” [33]. Instead of a maximum threshold, we might think instead about the difference in light exposure between a rat’s home cage and the room at large or environment we place them in. In a study by Barabas et al. [34], they measured the light intensity and other environmental factors in multiple facilities across a single institution. In the study, the room light intensity ranged from approximately 16 to 1400 lux. In the same study, the average light intensity inside a cage was quite low. The measurements depended on several factors but, generally, the top of the rack was brighter (13.21 lux) than the bottom of the rack (4.93 lux). If we consider an extreme example of removing a rat cage from as low as 5 lux and opening the cage on a tabletop in the 1400 lux room, this will require a substantial adjustment that could potentially be painful. Thus, considerations of lighting outside of the cage should also be considered in order to reduce negative experiences and improve rodent welfare. 

While our results support rats’ preference for lower intensity levels, we did not find as strong of evidence to support a preference for either light color. In the study by Kapogiannatou et al. [6], the rats preferred “warmer” temperature light (2500 K) compared to “cooler” light (4000 K). In our study, only rats raised in clear, high-intensity lighting conditions preferred the clear test chamber, which was the closest comparison to the cool light in the study by Kapogiannatou et al. It should be noted that no preference or aversion for the red-tinted caging was found in this study. This observation has implications for rodents continuously housed in red-tinted cages. While not preferred, there is a low likelihood that the cages are aversive but may affect their overall color vision. Using red-tinted cages may also call into question whether this in-cage illumination meets the guide, which states “Light in animal holding rooms should provide for both adequate vision and neuroendocrine regulation…” [33]. It is unclear whether appropriate color vision falls under this statement and unknown how the color filtering may affect eye development or perception. Thus, housing animals only in red-tinted caging is not recommended without further research. 

Our results also support the commonly held notion that eye pigmentation affects a rat’s perception of light. Even though we found a general preference for lower light levels across both types of rats tested, we found that the albino, CD, rats spent significantly more time in the low light condition compared to the pigmented, LE, rats. Again, the color preferences were less clear, with the CD rat preferences appearing to be contingent upon sex; the females preferred the clear chambers but the males showed no preference. The LE rats, however, did not show a significant color preference in either sex. It should be noted that the pigmented and albino stocks were genetically distinct, thus introducing the confound of other potential genetic components affecting their preferences aside from eye pigmentation.

While general findings for preferences were found, it is entirely likely that the rats may have chosen a particular environment based on behavior. For instance, we wanted to see if there was a difference in preference for a location when inactive, such as sleeping, compared to when active. We found some evidence that animals may choose for aspects of cage color depending on their behavior. However, this effect seemed to exist only in the animals raised in the high-intensity home cage lighting condition. The group of animals raised in the 200 lux lighting had a higher percentage of inactive observations in the clear vs. the red chambers. They were also observed to be more inactive in the clear test chamber compared to the rats raised in low lighting (25 lux). Finally, the rats raised in 200 lux were generally more inactive when they were in the clear test chamber. This seems to suggest that the rats raised in high-intensity lighting may prefer to rest in lighting conditions with a more diverse color make-up, which is consistent with our observation that these rats spent more time in the clear testing chamber overall. These observations may suggest a link between the intensity of home cage lighting and rodent preferences for lighting color when the light is bright. It is possible that due to the long exposure in the extreme condition, their ability to explore or adjust to a different environment might be altered. However, the result illustrating a shift in time spent in the 200 vs. the 25 lux environment over the course of the study illustrates that they do change, but it may be at a slower pace than the other animals tested. 

Other factors appeared to play a part in which behaviors were observed in which locations. Sex may also have played a role in where the rats spent their time. The males were observed to follow a pattern of spending about the same amount of time being active and inactive regardless of which test chamber they were in, but the females spent significantly more time inactive while in the clear test chamber than in the red. However, this effect is contextualized by the interactions among stock, sex, and behavior, which shows the same pattern independent of the test chamber color. The CD females spent significantly more time inactive than the LE females. Thus, it is not clear how much the difference in female inactivity was due to stock genetics or if the environment and color of chamber also influenced this. Taken together, our analyses of behavior fit with the overall conclusion that the cage color, and subsequently, the lighting make-up, is not a straightforward factor driving rodent lighting preferences. 

Finally, we expected the rodents to initially prefer lighting conditions similar to the cage in which they were raised, and that this would change over time and with experience. Generally, the frequency of observations for rats from one or the other home cage lighting conditions did not significantly differ from one time point to another. However, again, the rats housed in 200 lux did not fit the pattern of the other housing conditions. When first tested, the rats raised in 200 lux spent equal amounts of time in 200 and 25 lux. When tested for the second and third times, the rats spent significantly more time in 25 lux than 200. While minor, this does indicate a change from environments being perceived as equal to 25 lux being significantly preferred. The preference of the rats raised in 25 lux appeared to be relatively stable over time, showing a continued preference for 25 lux over all three tests. We considered running a linear contrast across the three time points to determine if a statistical linear slope exists, but since only three tests were conducted, we did not feel as if it was appropriate to try this analysis with so few time points. 

It is important to contextualize all of these results within the known limitations of preference testing. Our experimental procedures were designed to account for many of the problems of preference testing, but there remain some considerations that were not inherently addressed by our design. While we attempted to address environmental confounds by making the testing apparatus a close approximation of housing conditions, and by examining changes in preference over time to account for familiarity and novelty, we were only able to provide four distinct lighting conditions by combining two choices for color and two choices for intensity. As such, we can only make statements on the relative preference between the choices they were presented. It is possible that a lighting environment enriched with color that activates the UV cone might be more preferred than the choices offered in this experiment. We also cannot comment on the strength or importance of these preferences as we did not illicit any operant responses from the research animals; they were simply given free access to each possible combination of lighting conditions without any functional time limitation or significant obstacles. Thus, the preferences observed in this study should be considered the rats’ behaviors elicited in response to the specific conditions described and without significant cost for any given condition. Our study should be taken as an initial assessment of the importance of light intensity and color to rats in terms of their basic preference. 

## 5. Conclusions

Overall, the rats generally displayed a consistent choice of 25 over 200 lux, and their color preference appeared to be more nuanced. For instance, high-intensity home cage conditions may induce a color preference, and this observation may be more likely in females. In general, however, if researchers are seeking to improve the comfort and welfare of their animals through the manipulation of lighting conditions, it is obvious from these data that reducing the light intensity should be the first step rather than the unique spectral make-up of red-tinted cages, as this filters out the colors the animals can see. Since little is known about how this may affect eye development or physiology, the use of complete red caging should be implemented with caution. While color had little impact on choice, the few significant results show a preference for clear chambers, suggesting that light with a more diverse color make-up is desired.

## Data Availability

The original contributions presented in the study are included in the article or Supplementary Materials (Table S2); further inquiries can be directed to the corresponding author.

## Supplementary Materials

The following supporting information can be downloaded at: https://www.mdpi.com/article/10.3390/ani14142045/s1, Figure S1: Spectrograph of the four lighting conditions. Spectral figure showing the irradiance (μ watts/cm^2^/nm) for the given wavelength of light from 350 to 750 nm. Data are provided for each of the possible lighting conditions, with the dark red line representing the red/200 lux condition, dark grey representing the clear/200 lux condition, light grey representing the clear/25 lux condition, and light red representing the red/25 lux condition. The colored bars represent the general colors the rats see with their two types of cones that are sensitive to wavelengths at 510 and 359 nm. Table S1: Full statistical output with test statistics and *p* values. Table S2: SAS code for statistical models and accompanying data.

## References

[1] Fraser D., Weary D.M., Pajor E.A., Milligan B.N. (1997). A scientific conception of animal welfare that reflects ethical concerns. Anim. Welf..

[2] Peirson S.N., Brown L.A., Pothecary C.A., Benson L.A., Fisk A.S. (2018). Light and the laboratory mouse. J. Neurosci. Methods.

[3] Clough G. (1982). Environmental effects on animals used in biomedical research. Biol. Rev..

[4] Vanderschuren L.J., Niesink R.J., Spruijt B.M., Van Ree J.M. (1995). Influence of Environmental Factors on Social Play Behavior of Juvenile Rats. Physiol. Behav..

[5] Suckow M.A., Wolter W.R., Duffield G.E. (2017). The impact of environmental light intensity on experimental tumor growth. Anticancer Res..

[6] Kapogiannatou A., Paronis E., Paschidis K., Polissidis A., Kostomitsopoulos N.G. (2016). Effect of light colour temperature and intensity on τhε behaviour of male C57CL/6J mice. Appl. Anim. Behav. Sci..

[7] Roman E., Arborelius L. (2009). Male but not female Wistar rats show increased anxiety-like behaviour in response to bright light in the defensive withdrawal test. Behav. Brain Res..

[8] Penn J., Baker B., Howard A., Williams T. (1985). Retinal light-damage in albino rats: Lysosomal enzymes, rhodopsin, and age. Exp. Eye Res..

[9] Nestler E.J., Barrot M., Dileone R.J., Eisch A.J., Gold S.J., Monteggia L.M. (2002). Review Neurobiology of Depression the focus of efforts to understand the pathophysiology. Neurobiol. Depress. Neuron.

[10] Legates T.A., Altimus C.M., Wang H., Lee H.K., Yang S., Zhao H., Kirkwood A., Weber E.T., Hattar S. (2012). Aberrant light directly impairs mood and learning through melanopsin-expressing neurons. Nature.

[11] Pilorz V., Tam S.K.E., Hughes S., Pothecary C.A., Jagannath A., Hankins M.W., Bannerman D.M., Lightman S.L., Vyazovskiy V.V., Nolan P.M. (2016). Melanopsin Regulates Both Sleep-Promoting and Arousal-Promoting Responses to Light. PLoS Biol..

[12] Steel L.C.E., Tir S., Tam S.K.E., Bussell J.N., Spitschan M., Foster R.G., Peirson S.N. (2022). Effects of Cage Position and Light Transmission on Home Cage Activity and Circadian Entrainment in Mice. Front. Neurosci..

[13] Alves-Simoes M., Coleman G., Canal M.M. (2016). Effects of type of light on mouse circadian behaviour and stress levels. Lab. Anim..

[14] Dauchy R.T., Dauchy E.M., Hanifin J.P., Gauthreaux S.L., Mao L., Belancio V.P., Ooms T.G., Dupepe L.M., Jablonski M.R., Warfield B. (2013). Effects of Spectral Transmittance through Standard Laboratory Cages on Circadian Metabolism and Physiology in Nude Rats. J. Am. Assoc. Lab. Anim. Sci..

[15] Williams D.I. (1971). Maze exploration in the rat under different levels of illumination. Anim. Behav..

[16] Sherwin C.M., Glen E.F. (2003). Cage colour preferences and effects of home cage colour on anxiety in laboratory mice. Anim. Behav..

[17] Jacobs G.H., Neitz J., Deegan J.F. (1991). Retinal receptors in rodents maximally sensitive to ultraviolet light. Nature.

[18] Szél Á., Röhlich P. (1992). Two cone types of rat retina detected by anti-visual pigment antibodies. Exp. Eye Res..

[19] Schlingmann F., De Rijk H., Pereboom W., Remie R. (1993). Avoidance as a behavioural parameter in the determination of distress amongst albino and pigmented rats at various light intensities. Anim. Technol..

[20] Dawkins M. (1980). Environmental preference studies in the hen. Anim. Regul. Stud..

[21] LaFollette M.R., Swan M.P., Smith R.K., Hickman D.L., Gaskill B.N. (2019). The effects of cage color and light intensity on rat affect during heterospecific play. Appl. Anim. Behav. Sci..

[22] Neitz J., Jacobs G.H. (1986). Reexamination of spectral mechanisms in the rat (*Rattus norvegicus*). J. Comp. Psychol..

[23] Yokoyama S., Radlwimmer F.B., Kawamura S. (1998). Regeneration of ultraviolet pigments of vertebrates. FEBS Lett..

[24] Heiduschka P., Schraermeyer U. (2008). Comparison of visual function in pigmented and albino rats by electroretinography and visual evoked potentials. Graefe’s Arch. Clin. Exp. Ophthalmol..

[25] Jacobs G.H., Fenwick J.A., Williams G.A. (2001). Cone-based vision of rats for ultraviolet and visible lights. J. Exp. Biol..

[26] Walton W.E. (1933). Color vision and color preference in the albino rat. II. The experiments and results. J. Comp. Psychol..

[27] Rocha F.A.D., Gomes B.D., Silveira L.C.D., Martins S.L., Aguiar R.G., de Souza J.M., Ventura D.F. (2016). Spectral Sensitivity Measured with Electroretinogram Using a Constant Response Method. PLoS ONE.

[28] Panda S., Nayak S.K., Campo B., Walker J.R., Hogenesch J.B., Jegla T. (2005). Illumination of the Melanopsin Signaling Pathway. Science.

[29] Poeggeler B.H., Barlowwalden L.R., Reiter R.J., Saarela S., Menendezpelaez A., Yaga K., Manchester L.C., Chen L.D., Tan D.X. (1995). Red-Light-Induced Suppression of Melatonin Synthesis Is Mediated by N-Methyl-D-Aspartate Receptor Activation in Retinally Normal and Retinally Degenerate Rats. J. Neurobiol..

[30] Sun J.H., Yaga K., Reiter R.J., Garza M., Manchester L.C., Tan D.X., Poeggeler B. (1993). Reduction in pineal N-acetyltransferase activity and pineal and serum melatonin levels in rats after their exposure to red light at night. Neurosci. Lett..

[31] Hofstetter J.R., Hofstetter A.R., Hughes A.M., Mayeda A.R. (2005). Intermittent long-wavelength red light increases the period of daily locomotor activity in mice. J. Circadian Rhythm..

[32] Niklaus S., Albertini S., Schnitzer T., Denk N. (2020). Challenging a myth and misconception: Red-light vision in rats. Animals.

[33] National Research Council (2011). Guide for the Care and Use of Laboratory Animals: Eighth Edition.

[34] Barabas A.J., Darbyshire A.K., Schlegel S.L., Gaskill B.N. (2022). Evaluation of ambient sound, vibration, and light in rodent housing rooms. J. Am. Assoc. Lab. Anim. Sci..

