# Supplementary material for: The Future Is Not Bright: Evaluation of Rat Preferences for Color and Intensity of Light
Source: Animals (Basel). 2024 Jul 12;14(14):2045. doi: 10.3390/ani14142045 (PMC11273897; doi:10.3390/ani14142045)
Supplement: Supplementary file 1 [file animals-14-02045-s001.zip › Table S1 - Full statistical output with test statistics and p values.pdf]

Supplemental Table 2. Full results for the main analysis and the nesting analysis with separate tables for all Tukey post hoc comparisons. Exact p-values are reported with significant values bolded and denoted with an asterisk. Numbers for models correspond to results section of the manuscript

Preference model

This model includes location, behavior and time point within one statistical test.

Effect/Interaction	DF Numerator	DF Denominator	F Ratio	p-Value
Stock	1	2084.3296	6.0343	**0.0141***
Sex	1	169.3965	7.8241	**0.0058***
Housing Color	1	171.3577	1.2981	0.2561
Housing Intensity	1	170.9085	1.1758	0.2797
Light Phase	1	2086.4122	5.0197	**0.0252***
Color Preference	1	2088.0200	5.8760	**0.0154***
Lighting Preference	1	2087.3981	18.0858	**0.0***
Activity	1	2086.6116	1.2315	0.2672
Stock*Sex	1	2084.2484	0.6778	0.4104
Stock*Housing Color	1	2084.1597	3.9560	**0.0468***
Stock*Housing Intensity	1	2084.1409	2.0782	0.1496
Stock*Light Phase	1	2084.7240	0.4672	0.4943
Stock*Color Preference	1	2085.0558	2.0375	0.1536
Stock*Lighting Preference	1	2085.1938	8.0780	**0.0045***
Stock*Activity	1	2084.9261	5.0416	**0.0248***
Sex*Housing Color	1	17.5143	0.0572	0.8137
Sex*Housing Intensity	1	17.4706	0.0026	0.9596
Sex*Light Phase	1	2085.4639	0.4367	0.5088
Sex*Color Preference	1	2087.3659	9.2239	**0.0024***
Sex*Lighting Preference	1	2085.8627	15.1724	**0.0001***
Sex*Activity	1	2086.8280	6.0582	**0.0139***
Housing Color*Housing Intensity	1	17.6650	0.6211	0.4411
Housing Color*Light Phase	1	2085.6821	0.3691	0.5436
Housing Color*Color Preference	1	2087.7713	25.5954	**0.0***
Housing Color*Lighting Preference	1	2086.1729	6.3718	**0.0117***
Housing Color*Activity	1	2086.2407	0.1663	0.6834
Housing Intensity*Light Phase	1	2085.3632	1.1021	0.2939
Housing Intensity*Color Preference	1	2087.6373	29.0055	**0.0***
Housing Intensity*Lighting Preference	1	2085.9998	0.7742	0.379
Housing Intensity*Activity	1	2086.1066	2.3872	0.1225
Light Phase*Color Preference	1	2084.3232	0.1422	0.7061

Light Phase*Lighting Preference	1	2085.2984	2.7736	0.096
Light Phase*Activity	1	2084.7436	18.1132	**0.0***
Color Preference*Lighting Preference	1	2088.5082	8.9192	**0.0029***
Color Preference*Activity	1	2086.4825	5.5241	**0.0188***
Lighting Preference*Activity	1	2086.7814	36.8995	**0.0***
Stock*Sex*Color Preference	1	2085.0753	6.2685	**0.0124***
Stock*Sex*Activity	1	2084.5578	4.5612	**0.0328***
Stock*Housing Color*Lighting Preference	1	2085.1239	6.0057	**0.0143***
Stock*Light Phase*Activity	1	2085.0562	4.4765	**0.0345***
Sex*Housing Color*Color Preference	1	2087.4440	13.1226	**0.0003***
Sex*Color Preference*Activity	1	2086.2579	5.4418	**0.0198***
Housing Color*Housing Intensity*Color Preference	1	2087.3634	9.4636	**0.0021***
Housing Color*Housing Intensity*Lighting Preference	1	2086.3881	9.3348	**0.0023***
Housing Color*Housing Intensity*Activity	1	2086.1691	0.7374	0.3906
Housing Color*Color Preference*Lighting Preference	1	2088.3171	2.5802	0.1084
Housing Color*Color Preference*Activity	1	2087.2204	2.3518	0.1253
Housing Color*Lighting Preference*Activity	1	2086.5240	2.9524	0.0859
Housing Intensity*Color Preference*Lighting Preference	1	2088.2664	0.0337	0.8544
Housing Intensity*Color Preference*Activity	1	2086.5104	7.0272	**0.0081***
Housing Intensity*Lighting Preference*Activity	1	2086.2614	0.3206	0.5713
Color Preference*Lighting Preference*Activity	1	2087.0300	2.9368	0.0867
Timepoint	2	2089.7013	0.3519	0.7034
Housing Color*Timepoint	2	2089.4155	0.7918	0.4532
Timepoint*Housing Intensity	2	2089.5809	0.2032	0.8161
Timepoint*Color Preference	2	2086.6673	2.6142	0.0735
Timepoint*Lighting Preference	2	2086.3755	4.4973	**0.0112***
Timepoint*Stock	2	2084.9275	0.1902	0.8268
Timepoint*Sex	2	2090.0175	0.6299	0.5327

Nesting Model

Effect/Interaction	DF Numerator	DF Denominator	F Ratio	p-Value
Cage Number	16	240	0.2987	0.9964

Caging Treatment	1	240	0.5165	0.473
Lighting Treatment	1	240	5.3236	**0.0219***
Sex	1	240	0.2367	0.627
Location	3	240	2.8380	**0.0387***
Caging Treatment*Lighting Treatment	1	240	0.4960	0.482
Caging Treatment*Sex	1	240	0.6361	0.4259
Caging Treatment*Location	3	240	1.9117	0.1283
Lighting Treatment*Sex	1	240	0.0046	0.9461
Lighting Treatment*Location	3	240	1.1163	0.3431
Sex*Location	3	240	0.6887	0.5597
Caging Treatment*Lighting Treatment*Sex	1	240	0.8509	0.3572
Caging Treatment*Lighting Treatment*Location	3	240	4.0940	**0.0074***
Timepoint	1	240	0.0139	0.9061

Preference Tukey

Interaction being evaluated	Comparison Level 1	Comparison Level 2	p-Value
**Housing Color*Color Preference*Activity**	Clear,Clear,Active	Clear,Red,Active	0.8395
	Clear,Clear,Active	Clear,Red,Inactive	0.3714
	Clear,Clear,Inactive	Clear,Clear,Active	0.1106
	Clear,Clear,Inactive	Clear,Red,Active	**0.0197***
	Clear,Clear,Inactive	Clear,Red,Inactive	**0.0017***
	Clear,Clear,Inactive	Red,Clear,Active	0.5773
	Clear,Clear,Inactive	Red,Clear,Inactive	0.8965
	Clear,Clear,Inactive	Red,Red,Active	0.6574
	Clear,Clear,Inactive	Red,Red,Inactive	0.6362
	Clear,Red,Active	Clear,Red,Inactive	0.9559
	Red,Clear,Active	Clear,Clear,Active	1.0
	Red,Clear,Active	Clear,Red,Active	0.791
	Red,Clear,Active	Clear,Red,Inactive	0.3147
	Red,Clear,Inactive	Clear,Clear,Active	0.9987
	Red,Clear,Inactive	Clear,Red,Active	0.4597
	Red,Clear,Inactive	Clear,Red,Inactive	0.1115
	Red,Clear,Inactive	Red,Clear,Active	0.9953
	Red,Clear,Inactive	Red,Red,Active	0.9999
	Red,Clear,Inactive	Red,Red,Inactive	0.9999
	Red,Red,Active	Clear,Clear,Active	1.0
	Red,Red,Active	Clear,Red,Active	0.7079
	Red,Red,Active	Clear,Red,Inactive	0.2413
	Red,Red,Active	Red,Clear,Active	1.0
	Red,Red,Inactive	Clear,Clear,Active	1.0
	Red,Red,Inactive	Clear,Red,Active	0.7337

	Red,Red,Inactive	Clear,Red,Inactive	0.2623
	Red,Red,Inactive	Red,Clear,Active	1.0
**Housing Color*Color Preference*Lighting Preference**	Clear,Clear,200lux	Clear,Red,200lux	0.9977
	Clear,Clear,200lux	Clear,Red,25lux	0.9776
	Clear,Clear,200lux	Red,Clear,200lux	0.9841
	Clear,Clear,200lux	Red,Red,200lux	1.0
	Clear,Clear,25lux	Clear,Clear,200lux	**0.0057***
	Clear,Clear,25lux	Clear,Red,200lux	**0.002***
	Clear,Clear,25lux	Clear,Red,25lux	**0.0005***
	Clear,Clear,25lux	Red,Clear,200lux	**0.0044***
	Clear,Clear,25lux	Red,Clear,25lux	1.0
	Clear,Clear,25lux	Red,Red,200lux	**0.0075***
	Clear,Clear,25lux	Red,Red,25lux	0.9376
	Clear,Red,200lux	Clear,Red,25lux	1.0
	Clear,Red,200lux	Red,Clear,200lux	1.0
	Red,Clear,200lux	Clear,Red,25lux	1.0
	Red,Clear,25lux	Clear,Clear,200lux	0.1004
	Red,Clear,25lux	Clear,Red,200lux	**0.0051***
	Red,Clear,25lux	Clear,Red,25lux	**0.0014***
	Red,Clear,25lux	Red,Clear,200lux	**0.0004***
	Red,Clear,25lux	Red,Red,200lux	**0.019***
	Red,Clear,25lux	Red,Red,25lux	0.9865
	Red,Red,200lux	Clear,Red,200lux	0.9997
	Red,Red,200lux	Clear,Red,25lux	0.9972
	Red,Red,200lux	Red,Clear,200lux	0.999
	Red,Red,25lux	Clear,Clear,200lux	0.3689
	Red,Red,25lux	Clear,Red,200lux	0.1557
	Red,Red,25lux	Clear,Red,25lux	**0.0095***
	Red,Red,25lux	Red,Clear,200lux	0.0558
	Red,Red,25lux	Red,Red,200lux	0.1152
**Housing Color*Housing Intensity*Activity**	Clear,200Lux,Active	Clear,25Lux,Active	1.0
	Clear,200Lux,Inactive	Clear,200Lux,Active	0.9472
	Clear,200Lux,Inactive	Clear,25Lux,Active	0.9558
	Clear,200Lux,Inactive	Clear,25Lux,Inactive	0.9961
	Clear,200Lux,Inactive	Red,25Lux,Active	1.0
	Clear,200Lux,Inactive	Red,25Lux,Inactive	0.9978
	Clear,25Lux,Inactive	Clear,200Lux,Active	1.0
	Clear,25Lux,Inactive	Clear,25Lux,Active	0.9998
	Red,200Lux,Active	Clear,200Lux,Active	0.9777

	Red,200Lux,Active	Clear,25Lux,Active	0.974
	Red,200Lux,Active	Clear,25Lux,Inactive	0.9979
	Red,200Lux,Active	Red,25Lux,Active	1.0
	Red,200Lux,Active	Red,25Lux,Inactive	0.9924
	Red,200Lux,Inactive	Clear,200Lux,Active	0.3106
	Red,200Lux,Inactive	Clear,200Lux,Inactive	0.9164
	Red,200Lux,Inactive	Clear,25Lux,Active	0.3924
	Red,200Lux,Inactive	Clear,25Lux,Inactive	0.6267
	Red,200Lux,Inactive	Red,200Lux,Active	0.7513
	Red,200Lux,Inactive	Red,25Lux,Active	0.8564
	Red,200Lux,Inactive	Red,25Lux,Inactive	0.419
	Red,25Lux,Active	Clear,200Lux,Active	0.9952
	Red,25Lux,Active	Clear,25Lux,Active	0.9637
	Red,25Lux,Active	Clear,25Lux,Inactive	0.9977
	Red,25Lux,Active	Red,25Lux,Inactive	0.9882
	Red,25Lux,Inactive	Clear,200Lux,Active	1.0
	Red,25Lux,Inactive	Clear,25Lux,Active	1.0
**Housing Color*Housing Intensity*Color Preference**	Clear,200Lux,Clear	Clear,200Lux,Red	**0.0***
	Clear,200Lux,Clear	Clear,25Lux,Clear	**0.0025***
	Clear,200Lux,Clear	Clear,25Lux,Red	0.0903
	Clear,200Lux,Clear	Red,200Lux,Clear	0.8525
	Clear,200Lux,Clear	Red,200Lux,Red	0.2338
	Clear,200Lux,Clear	Red,25Lux,Clear	0.0959
	Clear,200Lux,Clear	Red,25Lux,Red	0.3276
	Clear,25Lux,Clear	Clear,200Lux,Red	0.4005
	Clear,25Lux,Red	Clear,200Lux,Red	0.0664
	Clear,25Lux,Red	Clear,25Lux,Clear	1.0
	Red,200Lux,Clear	Clear,200Lux,Red	**0.0006***
	Red,200Lux,Clear	Clear,25Lux,Clear	0.6384
	Red,200Lux,Clear	Clear,25Lux,Red	0.7453
	Red,200Lux,Clear	Red,200Lux,Red	0.9746
	Red,200Lux,Clear	Red,25Lux,Clear	0.4389
	Red,200Lux,Clear	Red,25Lux,Red	0.9741
	Red,200Lux,Red	Clear,200Lux,Red	**0.0196***
	Red,200Lux,Red	Clear,25Lux,Clear	0.9878
	Red,200Lux,Red	Clear,25Lux,Red	0.9963
	Red,200Lux,Red	Red,25Lux,Clear	0.9967
	Red,200Lux,Red	Red,25Lux,Red	1.0
	Red,25Lux,Clear	Clear,200Lux,Red	0.3305
	Red,25Lux,Clear	Clear,25Lux,Clear	1.0

	Red,25Lux,Red	Clear,200Lux,Red	0.0822
	Red,25Lux,Red	Clear,25Lux,Clear	0.9891
	Red,25Lux,Red	Clear,25Lux,Red	0.9972
	Red,25Lux,Red	Red,25Lux,Clear	0.9976
**Housing Color*Housing Intensity*Lighting Preference**	Clear,200Lux,200lux	Clear,200Lux,25lux	1.0
	Clear,200Lux,200lux	Clear,25Lux,200lux	0.2663
	Clear,200Lux,200lux	Red,200Lux,200lux	0.9307
	Clear,200Lux,200lux	Red,25Lux,200lux	0.7909
	Clear,200Lux,25lux	Clear,25Lux,200lux	0.4232
	Clear,200Lux,25lux	Red,200Lux,200lux	0.998
	Clear,200Lux,25lux	Red,25Lux,200lux	0.9043
	Clear,25Lux,25lux	Clear,200Lux,200lux	0.9119
	Clear,25Lux,25lux	Clear,200Lux,25lux	0.7474
	Clear,25Lux,25lux	Clear,25Lux,200lux	**0.0049***
	Clear,25Lux,25lux	Red,200Lux,200lux	0.4035
	Clear,25Lux,25lux	Red,25Lux,200lux	0.1066
	Red,200Lux,200lux	Clear,25Lux,200lux	0.9281
	Red,200Lux,200lux	Red,25Lux,200lux	0.9966
	Red,200Lux,25lux	Clear,200Lux,200lux	0.2638
	Red,200Lux,25lux	Clear,200Lux,25lux	**0.0115***
	Red,200Lux,25lux	Clear,25Lux,200lux	**0.0001***
	Red,200Lux,25lux	Clear,25Lux,25lux	0.9051
	Red,200Lux,25lux	Red,200Lux,200lux	**0.0046***
	Red,200Lux,25lux	Red,25Lux,200lux	**0.0002***
	Red,200Lux,25lux	Red,25Lux,25lux	0.9305
	Red,25Lux,200lux	Clear,25Lux,200lux	0.9962
	Red,25Lux,25lux	Clear,200Lux,200lux	0.89
	Red,25Lux,25lux	Clear,200Lux,25lux	0.7334
	Red,25Lux,25lux	Clear,25Lux,200lux	**0.0083***
	Red,25Lux,25lux	Clear,25Lux,25lux	1.0
	Red,25Lux,25lux	Red,200Lux,200lux	0.1582
	Red,25Lux,25lux	Red,25Lux,200lux	**0.0212***
**Housing Color*Lighting Preference*Activity**	Clear,200lux,Active	Clear,200lux,Inactive	0.9091
	Clear,200lux,Active	Red,200lux,Inactive	0.3326
	Clear,200lux,Inactive	Red,200lux,Inactive	0.949
	Clear,25lux,Active	Clear,200lux,Active	1.0
	Clear,25lux,Active	Clear,200lux,Inactive	0.9147
	Clear,25lux,Active	Red,200lux,Inactive	0.401
	Clear,25lux,Inactive	Clear,200lux,Active	0.1597

	Clear,25lux,Inactive	Clear,200lux,Inactive	**0.0098***
	Clear,25lux,Inactive	Clear,25lux,Active	0.0653
	Clear,25lux,Inactive	Red,200lux,Active	0.709
	Clear,25lux,Inactive	Red,200lux,Inactive	**0.0011***
	Clear,25lux,Inactive	Red,25lux,Active	0.9602
	Red,200lux,Active	Clear,200lux,Active	0.9973
	Red,200lux,Active	Clear,200lux,Inactive	0.6749
	Red,200lux,Active	Clear,25lux,Active	0.9999
	Red,200lux,Active	Red,200lux,Inactive	**0.0089***
	Red,25lux,Active	Clear,200lux,Active	0.8824
	Red,25lux,Active	Clear,200lux,Inactive	0.3418
	Red,25lux,Active	Clear,25lux,Active	0.8829
	Red,25lux,Active	Red,200lux,Active	0.9888
	Red,25lux,Active	Red,200lux,Inactive	**0.0045***
	Red,25lux,Inactive	Clear,200lux,Active	**0.0003***
	Red,25lux,Inactive	Clear,200lux,Inactive	**0.0***
	Red,25lux,Inactive	Clear,25lux,Active	**0.0***
	Red,25lux,Inactive	Clear,25lux,Inactive	0.241
	Red,25lux,Inactive	Red,200lux,Active	**0.0003***
	Red,25lux,Inactive	Red,200lux,Inactive	**0.0***
	Red,25lux,Inactive	Red,25lux,Active	**0.0004***
**Color Preference*Lighting Preference*Activity**	Clear,200lux,Active	Clear,200lux,Inactive	0.9999
	Clear,200lux,Active	Red,200lux,Inactive	0.6523
	Clear,200lux,Active	Red,25lux,Active	1.0
	Clear,200lux,Inactive	Red,200lux,Inactive	0.8417
	Clear,25lux,Active	Clear,200lux,Active	0.1853
	Clear,25lux,Active	Clear,200lux,Inactive	0.1104
	Clear,25lux,Active	Red,200lux,Active	0.9493
	Clear,25lux,Active	Red,200lux,Inactive	**0.0006***
	Clear,25lux,Active	Red,25lux,Active	0.111
	Clear,25lux,Active	Red,25lux,Inactive	1.0
	Clear,25lux,Inactive	Clear,200lux,Active	**0.0***
	Clear,25lux,Inactive	Clear,200lux,Inactive	**0.0***
	Clear,25lux,Inactive	Clear,25lux,Active	**0.002***
	Clear,25lux,Inactive	Red,200lux,Active	**0.0024***
	Clear,25lux,Inactive	Red,200lux,Inactive	**0.0***
	Clear,25lux,Inactive	Red,25lux,Active	**0.0***
	Clear,25lux,Inactive	Red,25lux,Inactive	**0.039***
	Red,200lux,Active	Clear,200lux,Active	0.863
	Red,200lux,Active	Clear,200lux,Inactive	0.7294

	Red,200lux,Active	Red,200lux,Inactive	**0.0003***
	Red,200lux,Active	Red,25lux,Active	0.7546
	Red,25lux,Active	Clear,200lux,Inactive	1.0
	Red,25lux,Active	Red,200lux,Inactive	0.7644
	Red,25lux,Inactive	Clear,200lux,Active	0.3995
	Red,25lux,Inactive	Clear,200lux,Inactive	0.2673
	Red,25lux,Inactive	Red,200lux,Active	0.9952
	Red,25lux,Inactive	Red,200lux,Inactive	**0.0032***
	Red,25lux,Inactive	Red,25lux,Active	**0.0202***
**Housing Intensity*Color Preference*Activity**	200Lux,Clear,Active	200Lux,Red,Active	0.5426
	200Lux,Clear,Active	200Lux,Red,Inactive	0.111
	200Lux,Clear,Active	25Lux,Clear,Active	0.9041
	200Lux,Clear,Active	25Lux,Clear,Inactive	0.784
	200Lux,Clear,Active	25Lux,Red,Active	0.9942
	200Lux,Clear,Active	25Lux,Red,Inactive	0.9915
	200Lux,Clear,Inactive	200Lux,Clear,Active	**0.0032***
	200Lux,Clear,Inactive	200Lux,Red,Active	**0.0***
	200Lux,Clear,Inactive	200Lux,Red,Inactive	**0.0***
	200Lux,Clear,Inactive	25Lux,Clear,Active	**0.0003***
	200Lux,Clear,Inactive	25Lux,Clear,Inactive	**0.0001***
	200Lux,Clear,Inactive	25Lux,Red,Active	**0.013***
	200Lux,Clear,Inactive	25Lux,Red,Inactive	**0.0119***
	200Lux,Red,Active	200Lux,Red,Inactive	0.9593
	25Lux,Clear,Active	200Lux,Red,Active	0.9994
	25Lux,Clear,Active	200Lux,Red,Inactive	0.8979
	25Lux,Clear,Active	25Lux,Clear,Inactive	1.0
	25Lux,Clear,Inactive	200Lux,Red,Active	1.0
	25Lux,Clear,Inactive	200Lux,Red,Inactive	0.9653
	25Lux,Red,Active	200Lux,Red,Active	0.9712
	25Lux,Red,Active	200Lux,Red,Inactive	0.563
	25Lux,Red,Active	25Lux,Clear,Active	0.9999
	25Lux,Red,Active	25Lux,Clear,Inactive	0.9981
	25Lux,Red,Active	25Lux,Red,Inactive	1.0
	25Lux,Red,Inactive	200Lux,Red,Active	0.9816
	25Lux,Red,Inactive	200Lux,Red,Inactive	0.6214
	25Lux,Red,Inactive	25Lux,Clear,Active	1.0
	25Lux,Red,Inactive	25Lux,Clear,Inactive	0.9991
**Housing Intensity*Color Preference*Lighting Preference**	200Lux,Clear,200lux	200Lux,Red,200lux	0.6135
	200Lux,Clear,200lux	200Lux,Red,25lux	0.8368

	200Lux,Clear,200lux	25Lux,Clear,200lux	**0.0219***
	200Lux,Clear,200lux	25Lux,Red,200lux	0.9028
	200Lux,Clear,25lux	200Lux,Clear,200lux	0.0898
	200Lux,Clear,25lux	200Lux,Red,200lux	**0.0002***
	200Lux,Clear,25lux	200Lux,Red,25lux	**0.0***
	200Lux,Clear,25lux	25Lux,Clear,200lux	**0.0***
	200Lux,Clear,25lux	25Lux,Clear,25lux	0.6199
	200Lux,Clear,25lux	25Lux,Red,200lux	**0.0003***
	200Lux,Clear,25lux	25Lux,Red,25lux	0.3349
	200Lux,Red,200lux	25Lux,Clear,200lux	0.9303
	200Lux,Red,25lux	200Lux,Red,200lux	1.0
	200Lux,Red,25lux	25Lux,Clear,200lux	0.7995
	25Lux,Clear,25lux	200Lux,Clear,200lux	0.9032
	25Lux,Clear,25lux	200Lux,Red,200lux	0.0606
	25Lux,Clear,25lux	200Lux,Red,25lux	0.168
	25Lux,Clear,25lux	25Lux,Clear,200lux	**0.0004***
	25Lux,Clear,25lux	25Lux,Red,200lux	0.0985
	25Lux,Clear,25lux	25Lux,Red,25lux	0.9822
	25Lux,Red,200lux	200Lux,Red,200lux	1.0
	25Lux,Red,200lux	25Lux,Clear,200lux	0.6545
	25Lux,Red,25lux	200Lux,Clear,200lux	0.9998
	25Lux,Red,25lux	200Lux,Red,200lux	0.3943
	25Lux,Red,25lux	200Lux,Red,25lux	0.3435
	25Lux,Red,25lux	25Lux,Clear,200lux	**0.0111***
	25Lux,Red,25lux	25Lux,Red,200lux	0.5247
**Housing Intensity*Lighting Preference*Activity**	200Lux,200lux,Active	200Lux,200lux,Inactive	0.56
	200Lux,200lux,Active	200Lux,25lux,Active	0.9987
	200Lux,200lux,Active	25Lux,200lux,Active	0.9325
	200Lux,200lux,Active	25Lux,200lux,Inactive	**0.0248***
	200Lux,200lux,Inactive	25Lux,200lux,Inactive	0.5835
	200Lux,25lux,Active	200Lux,200lux,Inactive	0.9963
	200Lux,25lux,Active	25Lux,200lux,Active	0.999
	200Lux,25lux,Active	25Lux,200lux,Inactive	0.129
	200Lux,25lux,Inactive	200Lux,200lux,Active	0.1385
	200Lux,25lux,Inactive	200Lux,200lux,Inactive	**0.0028***
	200Lux,25lux,Inactive	200Lux,25lux,Active	**0.0001***
	200Lux,25lux,Inactive	25Lux,200lux,Active	**0.0027***
	200Lux,25lux,Inactive	25Lux,200lux,Inactive	**0.0***
	200Lux,25lux,Inactive	25Lux,25lux,Active	0.2663
	200Lux,25lux,Inactive	25Lux,25lux,Inactive	0.9994

	25Lux,200lux,Active	200Lux,200lux,Inactive	1.0
	25Lux,200lux,Active	25Lux,200lux,Inactive	0.0673
	25Lux,25lux,Active	200Lux,200lux,Active	1.0
	25Lux,25lux,Active	200Lux,200lux,Inactive	0.705
	25Lux,25lux,Active	200Lux,25lux,Active	0.9764
	25Lux,25lux,Active	25Lux,200lux,Active	0.7682
	25Lux,25lux,Active	25Lux,200lux,Inactive	**0.0065***
	25Lux,25lux,Inactive	200Lux,200lux,Active	0.3764
	25Lux,25lux,Inactive	200Lux,200lux,Inactive	**0.0149***
	25Lux,25lux,Inactive	200Lux,25lux,Active	0.093
	25Lux,25lux,Inactive	25Lux,200lux,Active	**0.0166***
	25Lux,25lux,Inactive	25Lux,200lux,Inactive	**0.0***
	25Lux,25lux,Inactive	25Lux,25lux,Active	0.1683
**Timepoint*Housing Color*Color Preference**	1,Clear,Clear	1,Clear,Red	0.0697
	1,Clear,Clear	1,Red,Clear	0.9999
	1,Clear,Clear	1,Red,Red	0.999
	1,Clear,Clear	2,Clear,Red	0.1095
	1,Clear,Clear	2,Red,Clear	0.9732
	1,Clear,Clear	2,Red,Red	0.9997
	1,Clear,Clear	3,Clear,Red	0.284
	1,Clear,Clear	3,Red,Clear	**0.0264***
	1,Clear,Clear	3,Red,Red	1.0
	1,Clear,Red	3,Red,Clear	1.0
	1,Red,Clear	1,Clear,Red	0.3545
	1,Red,Clear	2,Clear,Red	0.4682
	1,Red,Clear	2,Red,Clear	1.0
	1,Red,Clear	3,Clear,Red	0.7666
	1,Red,Clear	3,Red,Clear	0.2033
	1,Red,Red	1,Clear,Red	0.4542
	1,Red,Red	2,Clear,Red	0.5772
	1,Red,Red	2,Red,Clear	1.0
	1,Red,Red	3,Clear,Red	0.8541
	1,Red,Red	3,Red,Clear	0.278
	2,Clear,Clear	1,Clear,Red	0.0543
	2,Clear,Clear	1,Red,Clear	0.9997
	2,Clear,Clear	1,Red,Red	0.9976
	2,Clear,Clear	2,Clear,Red	0.0862
	2,Clear,Clear	2,Red,Clear	0.9576
	2,Clear,Clear	2,Red,Red	0.9992
	2,Clear,Clear	3,Clear,Clear	1.0

	2,Clear,Clear	3,Clear,Red	0.237
	2,Clear,Clear	3,Red,Clear	**0.0197***
	2,Clear,Clear	3,Red,Red	1.0
	2,Clear,Red	3,Red,Clear	1.0
	2,Red,Clear	1,Clear,Red	0.7778
	2,Red,Clear	2,Clear,Red	0.8687
	2,Red,Clear	3,Clear,Red	0.9834
	2,Red,Clear	3,Red,Clear	0.5981
	2,Red,Red	1,Clear,Red	0.3927
	2,Red,Red	2,Clear,Red	0.5112
	2,Red,Red	2,Red,Clear	1.0
	2,Red,Red	3,Clear,Red	0.8043
	2,Red,Red	3,Red,Clear	0.2312
	3,Clear,Clear	1,Clear,Red	0.0975
	3,Clear,Clear	1,Red,Clear	1.0
	3,Clear,Clear	1,Red,Red	0.9998
	3,Clear,Clear	2,Clear,Red	0.149
	3,Clear,Clear	2,Red,Clear	0.9891
	3,Clear,Clear	2,Red,Red	1.0
	3,Clear,Clear	3,Clear,Red	0.3618
	3,Clear,Clear	3,Red,Clear	**0.0385***
	3,Clear,Red	1,Clear,Red	1.0
	3,Clear,Red	2,Clear,Red	1.0
	3,Clear,Red	3,Red,Clear	0.9992
	3,Red,Red	1,Clear,Red	0.1113
	3,Red,Red	1,Red,Clear	1.0
	3,Red,Red	1,Red,Red	0.9999
	3,Red,Red	2,Clear,Red	0.1685
	3,Red,Red	2,Red,Clear	0.9933
	3,Red,Red	2,Red,Red	1.0
	3,Red,Red	3,Clear,Red	0.3972
	3,Red,Red	3,Red,Clear	0.0503
**Timepoint*Color Preference*Lighting Preference**	1,Clear,200lux	2,Clear,200lux	0.6851
	1,Clear,200lux	2,Red,200lux	0.9993
	1,Clear,200lux	3,Clear,200lux	0.8831
	1,Clear,200lux	3,Red,200lux	0.8966
	1,Clear,25lux	1,Clear,200lux	**0.0003***
	1,Clear,25lux	1,Red,200lux	**0.0002***
	1,Clear,25lux	1,Red,25lux	**0.0353***
	1,Clear,25lux	2,Clear,200lux	**0.0***

1,Clear,25lux	2,Red,200lux	**0.0***
1,Clear,25lux	2,Red,25lux	0.5072
1,Clear,25lux	3,Clear,200lux	**0.0***
1,Clear,25lux	3,Clear,25lux	0.9855
1,Clear,25lux	3,Red,200lux	**0.0***
1,Clear,25lux	3,Red,25lux	1.0
1,Red,200lux	2,Clear,200lux	0.7145
1,Red,200lux	2,Red,200lux	0.9996
1,Red,200lux	3,Clear,200lux	0.9015
1,Red,200lux	3,Red,200lux	0.9132
1,Red,25lux	1,Clear,200lux	0.9827
1,Red,25lux	1,Red,200lux	0.9728
1,Red,25lux	2,Clear,200lux	**0.0431***
1,Red,25lux	2,Red,200lux	0.5731
1,Red,25lux	3,Clear,200lux	0.1143
1,Red,25lux	3,Red,200lux	0.1246
2,Clear,25lux	1,Clear,200lux	**0.0***
2,Clear,25lux	1,Clear,25lux	0.9303
2,Clear,25lux	1,Red,200lux	**0.0***
2,Clear,25lux	1,Red,25lux	**0.0001***
2,Clear,25lux	2,Clear,200lux	**0.0***
2,Clear,25lux	2,Red,200lux	**0.0***
2,Clear,25lux	2,Red,25lux	**0.0088***
2,Clear,25lux	3,Clear,200lux	**0.0***
2,Clear,25lux	3,Clear,25lux	0.1941
2,Clear,25lux	3,Red,200lux	**0.0***
2,Clear,25lux	3,Red,25lux	0.7072
2,Red,200lux	2,Clear,200lux	0.9932
2,Red,200lux	3,Clear,200lux	0.9998
2,Red,200lux	3,Red,200lux	0.9999
2,Red,25lux	1,Clear,200lux	0.4109
2,Red,25lux	1,Red,200lux	0.3523
2,Red,25lux	1,Red,25lux	0.9931
2,Red,25lux	2,Clear,200lux	**0.0005***
2,Red,25lux	2,Red,200lux	**0.0453***
2,Red,25lux	3,Clear,200lux	**0.0022***
2,Red,25lux	3,Red,200lux	**0.0025***
3,Clear,200lux	2,Clear,200lux	1.0
3,Clear,25lux	1,Clear,200lux	**0.035***
3,Clear,25lux	1,Red,200lux	**0.0254***
3,Clear,25lux	1,Red,25lux	0.5935

	3,Clear,25lux	2,Clear,200lux	**0.0***
	3,Clear,25lux	2,Red,200lux	**0.0011***
	3,Clear,25lux	2,Red,25lux	0.9974
	3,Clear,25lux	3,Clear,200lux	**0.0***
	3,Clear,25lux	3,Red,200lux	**0.0***
	3,Red,200lux	2,Clear,200lux	1.0
	3,Red,25lux	1,Clear,200lux	**0.0017***
	3,Red,25lux	1,Red,200lux	**0.0011***
	3,Red,25lux	1,Red,25lux	0.1226
	3,Red,25lux	2,Clear,200lux	**0.0***
	3,Red,25lux	2,Red,200lux	**0.0***
	3,Red,25lux	2,Red,25lux	0.8036
	3,Red,25lux	3,Clear,200lux	**0.0***
	3,Red,25lux	3,Clear,25lux	0.9997
	3,Red,25lux	3,Red,200lux	**0.0***
**Timepoint*Housing Intensity*Lighting Preference**	1,200Lux,200lux	1,25Lux,200lux	0.8348
	1,200Lux,200lux	2,200Lux,200lux	0.9503
	1,200Lux,200lux	2,25Lux,200lux	0.0834
	1,200Lux,200lux	3,200Lux,200lux	0.1474
	1,200Lux,200lux	3,25Lux,200lux	0.622
	1,200Lux,25lux	1,200Lux,200lux	0.7407
	1,200Lux,25lux	1,25Lux,200lux	**0.0083***
	1,200Lux,25lux	2,200Lux,200lux	**0.0287***
	1,200Lux,25lux	2,25Lux,200lux	**0.0***
	1,200Lux,25lux	3,200Lux,200lux	**0.0001***
	1,200Lux,25lux	3,25Lux,200lux	**0.0023***
	1,25Lux,200lux	2,25Lux,200lux	0.9669
	1,25Lux,200lux	3,200Lux,200lux	0.9922
	1,25Lux,200lux	3,25Lux,200lux	1.0
	1,25Lux,25lux	1,200Lux,200lux	0.545
	1,25Lux,25lux	1,200Lux,25lux	1.0
	1,25Lux,25lux	1,25Lux,200lux	**0.0028***
	1,25Lux,25lux	2,200Lux,200lux	**0.0096***
	1,25Lux,25lux	2,25Lux,200lux	**0.0***
	1,25Lux,25lux	3,200Lux,200lux	**0.0***
	1,25Lux,25lux	3,25Lux,200lux	**0.0007***
	1,25Lux,25lux	3,25Lux,25lux	1.0
	2,200Lux,200lux	1,25Lux,200lux	1.0
	2,200Lux,200lux	2,25Lux,200lux	0.8865
	2,200Lux,200lux	3,200Lux,200lux	0.9595

	2,200Lux,200lux	3,25Lux,200lux	1.0
	2,200Lux,25lux	1,200Lux,200lux	0.2094
	2,200Lux,25lux	1,200Lux,25lux	0.9997
	2,200Lux,25lux	1,25Lux,200lux	**0.0002***
	2,200Lux,25lux	1,25Lux,25lux	1.0
	2,200Lux,25lux	2,200Lux,200lux	**0.0012***
	2,200Lux,25lux	2,25Lux,200lux	**0.0***
	2,200Lux,25lux	3,200Lux,200lux	**0.0***
	2,200Lux,25lux	3,25Lux,200lux	**0.0***
	2,200Lux,25lux	3,25Lux,25lux	0.9996
	2,25Lux,25lux	1,200Lux,200lux	**0.0038***
	2,25Lux,25lux	1,200Lux,25lux	0.6244
	2,25Lux,25lux	1,25Lux,200lux	**0.0***
	2,25Lux,25lux	1,25Lux,25lux	0.7788
	2,25Lux,25lux	2,200Lux,200lux	**0.0***
	2,25Lux,25lux	2,200Lux,25lux	0.9779
	2,25Lux,25lux	2,25Lux,200lux	**0.0***
	2,25Lux,25lux	3,200Lux,200lux	**0.0***
	2,25Lux,25lux	3,25Lux,200lux	**0.0***
	2,25Lux,25lux	3,25Lux,25lux	0.6076
	3,200Lux,200lux	2,25Lux,200lux	1.0
	3,200Lux,25lux	1,200Lux,200lux	**0.006***
	3,200Lux,25lux	1,200Lux,25lux	0.7142
	3,200Lux,25lux	1,25Lux,200lux	**0.0***
	3,200Lux,25lux	1,25Lux,25lux	0.8408
	3,200Lux,25lux	2,200Lux,200lux	**0.0***
	3,200Lux,25lux	2,200Lux,25lux	0.9907
	3,200Lux,25lux	2,25Lux,200lux	**0.0***
	3,200Lux,25lux	3,200Lux,200lux	**0.0***
	3,200Lux,25lux	3,25Lux,200lux	**0.0***
	3,200Lux,25lux	3,25Lux,25lux	0.6842
	3,25Lux,200lux	2,25Lux,200lux	0.9973
	3,25Lux,200lux	3,200Lux,200lux	0.9998
	3,25Lux,25lux	1,200Lux,200lux	0.7325
	3,25Lux,25lux	1,25Lux,200lux	**0.0083***
	3,25Lux,25lux	2,200Lux,200lux	**0.0255***
	3,25Lux,25lux	2,25Lux,200lux	**0.0***
	3,25Lux,25lux	3,200Lux,200lux	**0.0***
	3,25Lux,25lux	3,25Lux,200lux	**0.0023***
**Sex*Housing Color*Color Preference**	Female,Clear,Clear	Female,Clear,Red	**0.0***
	Female,Clear,Clear	Female,Red,Clear	0.5897

	Female,Clear,Clear	Female,Red,Red	0.6223
	Female,Clear,Clear	Male,Clear,Clear	**0.0***
	Female,Clear,Clear	Male,Clear,Red	**0.0033***
	Female,Clear,Clear	Male,Red,Clear	0.0863
	Female,Clear,Clear	Male,Red,Red	**0.0242***
	Female,Red,Clear	Female,Clear,Red	**0.0223***
	Female,Red,Clear	Male,Clear,Clear	0.7503
	Female,Red,Clear	Male,Clear,Red	0.4646
	Female,Red,Clear	Male,Red,Clear	0.8355
	Female,Red,Clear	Male,Red,Red	0.8488
	Female,Red,Red	Female,Clear,Red	**0.0161***
	Female,Red,Red	Female,Red,Clear	1.0
	Female,Red,Red	Male,Clear,Clear	0.6983
	Female,Red,Red	Male,Clear,Red	0.4075
	Female,Red,Red	Male,Red,Clear	0.9591
	Female,Red,Red	Male,Red,Red	0.3991
	Male,Clear,Clear	Female,Clear,Red	0.6772
	Male,Clear,Clear	Male,Clear,Red	0.9999
	Male,Clear,Red	Female,Clear,Red	0.5823
	Male,Red,Clear	Female,Clear,Red	0.3007
	Male,Red,Clear	Male,Clear,Clear	0.9991
	Male,Red,Clear	Male,Clear,Red	0.9727
	Male,Red,Clear	Male,Red,Red	0.9999
	Male,Red,Red	Female,Clear,Red	0.5201
	Male,Red,Red	Male,Clear,Clear	1.0
	Male,Red,Red	Male,Clear,Red	0.9982
**Sex*Color Preference*Activity**	Female,Clear,Active	Female,Red,Active	0.9641
	Female,Clear,Active	Female,Red,Inactive	**0.0132***
	Female,Clear,Active	Male,Clear,Active	**0.0205***
	Female,Clear,Active	Male,Clear,Inactive	0.8628
	Female,Clear,Active	Male,Red,Active	**0.0349***
	Female,Clear,Active	Male,Red,Inactive	0.7402
	Female,Clear,Inactive	Female,Clear,Active	0.7065
	Female,Clear,Inactive	Female,Red,Active	0.2367
	Female,Clear,Inactive	Female,Red,Inactive	**0.0001***
	Female,Clear,Inactive	Male,Clear,Active	**0.0***
	Female,Clear,Inactive	Male,Clear,Inactive	0.0557
	Female,Clear,Inactive	Male,Red,Active	**0.0002***
	Female,Clear,Inactive	Male,Red,Inactive	0.065
	Female,Red,Active	Female,Red,Inactive	0.058
	Female,Red,Active	Male,Clear,Active	0.7399

	Female,Red,Active	Male,Clear,Inactive	1.0
	Female,Red,Active	Male,Red,Active	0.1582
	Female,Red,Active	Male,Red,Inactive	0.9971
	Male,Clear,Active	Female,Red,Inactive	0.9895
	Male,Clear,Active	Male,Red,Active	0.9998
	Male,Clear,Inactive	Female,Red,Inactive	0.2953
	Male,Clear,Inactive	Male,Clear,Active	0.6025
	Male,Clear,Inactive	Male,Red,Active	0.5189
	Male,Clear,Inactive	Male,Red,Inactive	0.9998
	Male,Red,Active	Female,Red,Inactive	0.9997
	Male,Red,Inactive	Female,Red,Inactive	0.3166
	Male,Red,Inactive	Male,Clear,Active	0.9713
	Male,Red,Inactive	Male,Red,Active	0.6073
**Stock*Housing Color*Lighting Preference**	CD,Clear,200lux	CD,Red,200lux	0.4584
	CD,Clear,200lux	LE,Clear,200lux	0.0747
	CD,Clear,200lux	LE,Clear,25lux	0.9982
	CD,Clear,200lux	LE,Red,200lux	0.989
	CD,Clear,25lux	CD,Clear,200lux	0.407
	CD,Clear,25lux	CD,Red,200lux	**0.0082***
	CD,Clear,25lux	LE,Clear,200lux	**0.0002***
	CD,Clear,25lux	LE,Clear,25lux	**0.0144***
	CD,Clear,25lux	LE,Red,200lux	0.1592
	CD,Clear,25lux	LE,Red,25lux	0.9529
	CD,Red,200lux	LE,Clear,200lux	1.0
	CD,Red,25lux	CD,Clear,200lux	**0.007***
	CD,Red,25lux	CD,Clear,25lux	0.4393
	CD,Red,25lux	CD,Red,200lux	**0.0***
	CD,Red,25lux	LE,Clear,200lux	**0.0***
	CD,Red,25lux	LE,Clear,25lux	**0.0***
	CD,Red,25lux	LE,Red,200lux	**0.0***
	CD,Red,25lux	LE,Red,25lux	**0.0022***
	LE,Clear,25lux	CD,Red,200lux	0.9319
	LE,Clear,25lux	LE,Clear,200lux	0.6669
	LE,Clear,25lux	LE,Red,200lux	1.0
	LE,Red,200lux	CD,Red,200lux	0.8347
	LE,Red,200lux	LE,Clear,200lux	0.7888
	LE,Red,25lux	CD,Clear,200lux	0.987
	LE,Red,25lux	CD,Red,200lux	**0.0447***
	LE,Red,25lux	LE,Clear,200lux	0.0606
	LE,Red,25lux	LE,Clear,25lux	0.6913

	LE,Red,25lux	LE,Red,200lux	0.548
**Stock*Light Phase*Activity**	CD,Dark,Active	LE,Dark,Active	0.5792
	CD,Dark,Active	LE,Light,Inactive	0.7755
	CD,Dark,Inactive	CD,Dark,Active	0.1049
	CD,Dark,Inactive	CD,Light,Active	1.0
	CD,Dark,Inactive	LE,Dark,Active	**0.0001***
	CD,Dark,Inactive	LE,Dark,Inactive	0.5878
	CD,Dark,Inactive	LE,Light,Active	0.9992
	CD,Dark,Inactive	LE,Light,Inactive	**0.0007***
	CD,Light,Active	CD,Dark,Active	0.1595
	CD,Light,Active	LE,Dark,Active	**0.0003***
	CD,Light,Active	LE,Dark,Inactive	0.7143
	CD,Light,Active	LE,Light,Active	1.0
	CD,Light,Active	LE,Light,Inactive	**0.0013***
	CD,Light,Inactive	CD,Dark,Active	**0.0318***
	CD,Light,Inactive	CD,Dark,Inactive	0.9999
	CD,Light,Inactive	CD,Light,Active	0.9981
	CD,Light,Inactive	LE,Dark,Active	**0.0***
	CD,Light,Inactive	LE,Dark,Inactive	0.3182
	CD,Light,Inactive	LE,Light,Active	0.9753
	CD,Light,Inactive	LE,Light,Inactive	**0.0001***
	LE,Dark,Inactive	CD,Dark,Active	0.9895
	LE,Dark,Inactive	LE,Dark,Active	0.1354
	LE,Dark,Inactive	LE,Light,Inactive	0.2676
	LE,Light,Active	CD,Dark,Active	0.3298
	LE,Light,Active	LE,Dark,Active	**0.0012***
	LE,Light,Active	LE,Dark,Inactive	0.8933
	LE,Light,Active	LE,Light,Inactive	**0.005***
	LE,Light,Inactive	LE,Dark,Active	1.0
**Stock*Sex*Activity**	CD,Female,Active	CD,Male,Active	0.3045
	CD,Female,Active	CD,Male,Inactive	1.0
	CD,Female,Active	LE,Female,Active	0.9999
	CD,Female,Active	LE,Female,Inactive	**0.0261***
	CD,Female,Active	LE,Male,Active	**0.0013***
	CD,Female,Active	LE,Male,Inactive	0.3618
	CD,Female,Inactive	CD,Female,Active	0.7994
	CD,Female,Inactive	CD,Male,Active	**0.0045***
	CD,Female,Inactive	CD,Male,Inactive	0.6179
	CD,Female,Inactive	LE,Female,Active	0.5269
	CD,Female,Inactive	LE,Female,Inactive	**0.0001***
	CD,Female,Inactive	LE,Male,Active	**0.0***

	CD,Female,Inactive	LE,Male,Inactive	**0.0074***
	CD,Male,Active	LE,Female,Inactive	0.9654
	CD,Male,Active	LE,Male,Active	0.6391
	CD,Male,Inactive	CD,Male,Active	0.555
	CD,Male,Inactive	LE,Female,Active	1.0
	CD,Male,Inactive	LE,Female,Inactive	0.0748
	CD,Male,Inactive	LE,Male,Active	**0.0073***
	CD,Male,Inactive	LE,Male,Inactive	0.6107
	LE,Female,Active	CD,Male,Active	0.5837
	LE,Female,Active	LE,Female,Inactive	0.0871
	LE,Female,Active	LE,Male,Active	**0.0069***
	LE,Female,Active	LE,Male,Inactive	0.6419
	LE,Female,Inactive	LE,Male,Active	0.9974
	LE,Male,Inactive	LE,Female,Inactive	0.9653
	LE,Male,Inactive	LE,Male,Active	0.653
**Stock*Sex*Color Preference**	CD,Female,Clear	CD,Female,Red	**0.0181***
	CD,Female,Clear	CD,Male,Clear	**0.0***
	CD,Female,Clear	CD,Male,Red	0.0996
	CD,Female,Clear	LE,Female,Clear	**0.0376***
	CD,Female,Clear	LE,Female,Red	**0.0***
	CD,Female,Clear	LE,Male,Clear	**0.0001***
	CD,Female,Clear	LE,Male,Red	**0.0***
	CD,Female,Red	CD,Male,Clear	0.9996
	CD,Female,Red	LE,Female,Red	0.4411
	CD,Female,Red	LE,Male,Clear	0.9999
	CD,Female,Red	LE,Male,Red	0.0501
	CD,Male,Clear	LE,Female,Red	0.9435
	CD,Male,Clear	LE,Male,Red	0.5331
	CD,Male,Red	CD,Female,Red	0.995
	CD,Male,Red	CD,Male,Clear	0.9495
	CD,Male,Red	LE,Female,Red	0.0869
	CD,Male,Red	LE,Male,Clear	0.9753
	CD,Male,Red	LE,Male,Red	**0.0042***
	LE,Female,Clear	CD,Female,Red	0.9913
	LE,Female,Clear	CD,Male,Clear	0.7038
	LE,Female,Clear	CD,Male,Red	1.0
	LE,Female,Clear	LE,Female,Red	0.1913
	LE,Female,Clear	LE,Male,Clear	0.8077
	LE,Female,Clear	LE,Male,Red	**0.0212***
	LE,Female,Red	LE,Male,Red	0.9807
	LE,Male,Clear	CD,Male,Clear	1.0

LE,Male,Clear	LE,Female,Red	0.9004
LE,Male,Clear	LE,Male,Red	0.4381

Nesting Tukey

Comparison Level 1	Comparison Level 2	p-Value
Clear,200Lux,Clear-200lux	Clear,200Lux,Clear-25lux	1.0
Clear,200Lux,Clear-200lux	Clear,200Lux,Red-200lux	**0.0056***
Clear,200Lux,Clear-200lux	Clear,200Lux,Red-25lux	0.4697
Clear,200Lux,Clear-200lux	Clear,25Lux,Clear-200lux	0.9997
Clear,200Lux,Clear-200lux	Clear,25Lux,Clear-25lux	1.0
Clear,200Lux,Clear-200lux	Clear,25Lux,Red-200lux	0.996
Clear,200Lux,Clear-200lux	Red,200Lux,Clear-200lux	0.9821
Clear,200Lux,Clear-200lux	Red,200Lux,Clear-25lux	0.9098
Clear,200Lux,Clear-200lux	Red,200Lux,Red-200lux	0.9946
Clear,200Lux,Clear-200lux	Red,200Lux,Red-25lux	1.0
Clear,200Lux,Clear-200lux	Red,25Lux,Clear-200lux	1.0
Clear,200Lux,Clear-200lux	Red,25Lux,Red-200lux	0.9997
Clear,200Lux,Clear-200lux	Red,25Lux,Red-25lux	1.0
Clear,200Lux,Clear-25lux	Clear,200Lux,Red-200lux	**0.015***
Clear,200Lux,Clear-25lux	Clear,200Lux,Red-25lux	0.671
Clear,200Lux,Clear-25lux	Clear,25Lux,Clear-200lux	1.0
Clear,200Lux,Clear-25lux	Clear,25Lux,Clear-25lux	1.0
Clear,200Lux,Clear-25lux	Clear,25Lux,Red-200lux	0.9997
Clear,200Lux,Clear-25lux	Red,200Lux,Clear-200lux	0.9977
Clear,200Lux,Clear-25lux	Red,200Lux,Clear-25lux	0.9766
Clear,200Lux,Clear-25lux	Red,200Lux,Red-200lux	0.9996
Clear,200Lux,Clear-25lux	Red,25Lux,Clear-200lux	1.0

Clear,200Lux,Clear-25lux	Red,25Lux,Red-200lux	1.0
Clear,200Lux,Clear-25lux	Red,25Lux,Red-25lux	1.0
Clear,200Lux,Red-25lux	Clear,200Lux,Red-200lux	0.968
Clear,25Lux,Clear-200lux	Clear,200Lux,Red-200lux	0.1738
Clear,25Lux,Clear-200lux	Clear,200Lux,Red-25lux	0.9876
Clear,25Lux,Clear-200lux	Clear,25Lux,Red-200lux	1.0
Clear,25Lux,Clear-200lux	Red,200Lux,Clear-200lux	1.0
Clear,25Lux,Clear-200lux	Red,200Lux,Clear-25lux	1.0
Clear,25Lux,Clear-200lux	Red,200Lux,Red-200lux	1.0
Clear,25Lux,Clear-200lux	Red,25Lux,Red-200lux	1.0
Clear,25Lux,Clear-25lux	Clear,200Lux,Red-200lux	0.0629
Clear,25Lux,Clear-25lux	Clear,200Lux,Red-25lux	0.9076
Clear,25Lux,Clear-25lux	Clear,25Lux,Clear-200lux	1.0
Clear,25Lux,Clear-25lux	Clear,25Lux,Red-200lux	1.0
Clear,25Lux,Clear-25lux	Red,200Lux,Clear-200lux	1.0
Clear,25Lux,Clear-25lux	Red,200Lux,Clear-25lux	0.999
Clear,25Lux,Clear-25lux	Red,200Lux,Red-200lux	1.0
Clear,25Lux,Clear-25lux	Red,25Lux,Clear-200lux	1.0
Clear,25Lux,Clear-25lux	Red,25Lux,Red-200lux	1.0
Clear,25Lux,Clear-25lux	Red,25Lux,Red-25lux	1.0
Clear,25Lux,Red-200lux	Clear,200Lux,Red-200lux	0.3075
Clear,25Lux,Red-200lux	Clear,200Lux,Red-25lux	0.9985
Clear,25Lux,Red-200lux	Red,200Lux,Clear-200lux	1.0
Clear,25Lux,Red-200lux	Red,200Lux,Clear-25lux	1.0
Clear,25Lux,Red-200lux	Red,200Lux,Red-200lux	1.0
Clear,25Lux,Red-25lux	Clear,200Lux,Clear-200lux	1.0
Clear,25Lux,Red-25lux	Clear,200Lux,Clear-25lux	1.0
Clear,25Lux,Red-25lux	Clear,200Lux,Red-200lux	**0.0011***
Clear,25Lux,Red-25lux	Clear,200Lux,Red-25lux	0.2019

Clear,25Lux,Red-25lux	Clear,25Lux,Clear-200lux	0.9832
Clear,25Lux,Red-25lux	Clear,25Lux,Clear-25lux	0.9992
Clear,25Lux,Red-25lux	Clear,25Lux,Red-200lux	0.9329
Clear,25Lux,Red-25lux	Red,200Lux,Clear-200lux	0.8497
Clear,25Lux,Red-25lux	Red,200Lux,Clear-25lux	0.653
Clear,25Lux,Red-25lux	Red,200Lux,Red-200lux	0.9202
Clear,25Lux,Red-25lux	Red,200Lux,Red-25lux	1.0
Clear,25Lux,Red-25lux	Red,25Lux,Clear-200lux	0.9966
Clear,25Lux,Red-25lux	Red,25Lux,Clear-25lux	1.0
Clear,25Lux,Red-25lux	Red,25Lux,Red-200lux	0.9831
Clear,25Lux,Red-25lux	Red,25Lux,Red-25lux	0.9992
Red,200Lux,Clear-200lux	Clear,200Lux,Red-200lux	0.3996
Red,200Lux,Clear-200lux	Clear,200Lux,Red-25lux	0.9998
Red,200Lux,Clear-200lux	Red,200Lux,Clear-25lux	1.0
Red,200Lux,Clear-25lux	Clear,200Lux,Red-200lux	0.6317
Red,200Lux,Clear-25lux	Clear,200Lux,Red-25lux	1.0
Red,200Lux,Red-200lux	Clear,200Lux,Red-200lux	0.2907
Red,200Lux,Red-200lux	Clear,200Lux,Red-25lux	0.9985
Red,200Lux,Red-200lux	Red,200Lux,Clear-200lux	1.0
Red,200Lux,Red-200lux	Red,200Lux,Clear-25lux	1.0
Red,200Lux,Red-25lux	Clear,200Lux,Clear-25lux	1.0
Red,200Lux,Red-25lux	Clear,200Lux,Red-200lux	**0.0113***
Red,200Lux,Red-25lux	Clear,200Lux,Red-25lux	0.6132
Red,200Lux,Red-25lux	Clear,25Lux,Clear-200lux	1.0
Red,200Lux,Red-25lux	Clear,25Lux,Clear-25lux	1.0
Red,200Lux,Red-25lux	Clear,25Lux,Red-200lux	0.9993
Red,200Lux,Red-25lux	Red,200Lux,Clear-200lux	0.9955
Red,200Lux,Red-25lux	Red,200Lux,Clear-25lux	0.9634
Red,200Lux,Red-25lux	Red,200Lux,Red-200lux	0.999
Red,200Lux,Red-25lux	Red,25Lux,Clear-200lux	1.0
Red,200Lux,Red-25lux	Red,25Lux,Red-200lux	1.0
Red,200Lux,Red-25lux	Red,25Lux,Red-25lux	1.0
Red,25Lux,Clear-200lux	Clear,200Lux,Red-200lux	0.1003

Red,25Lux,Clear-200lux	Clear,200Lux,Red-25lux	0.9563
Red,25Lux,Clear-200lux	Clear,25Lux,Clear-200lux	1.0
Red,25Lux,Clear-200lux	Clear,25Lux,Red-200lux	1.0
Red,25Lux,Clear-200lux	Red,200Lux,Clear-200lux	1.0
Red,25Lux,Clear-200lux	Red,200Lux,Clear-25lux	0.9999
Red,25Lux,Clear-200lux	Red,200Lux,Red-200lux	1.0
Red,25Lux,Clear-200lux	Red,25Lux,Red-200lux	1.0
Red,25Lux,Clear-25lux	Clear,200Lux,Clear-200lux	1.0
Red,25Lux,Clear-25lux	Clear,200Lux,Clear-25lux	1.0
Red,25Lux,Clear-25lux	Clear,200Lux,Red-200lux	**0.0062***
Red,25Lux,Clear-25lux	Clear,200Lux,Red-25lux	0.4703
Red,25Lux,Clear-25lux	Clear,25Lux,Clear-200lux	0.9996
Red,25Lux,Clear-25lux	Clear,25Lux,Clear-25lux	1.0
Red,25Lux,Clear-25lux	Clear,25Lux,Red-200lux	0.9953
Red,25Lux,Clear-25lux	Red,200Lux,Clear-200lux	0.9802
Red,25Lux,Clear-25lux	Red,200Lux,Clear-25lux	0.9056
Red,25Lux,Clear-25lux	Red,200Lux,Red-200lux	0.9937
Red,25Lux,Clear-25lux	Red,200Lux,Red-25lux	1.0
Red,25Lux,Clear-25lux	Red,25Lux,Clear-200lux	1.0
Red,25Lux,Clear-25lux	Red,25Lux,Red-200lux	0.9996
Red,25Lux,Clear-25lux	Red,25Lux,Red-25lux	1.0
Red,25Lux,Red-200lux	Clear,200Lux,Red-200lux	0.1758
Red,25Lux,Red-200lux	Clear,200Lux,Red-25lux	0.988
Red,25Lux,Red-200lux	Clear,25Lux,Red-200lux	1.0
Red,25Lux,Red-200lux	Red,200Lux,Clear-200lux	1.0
Red,25Lux,Red-200lux	Red,200Lux,Clear-25lux	1.0
Red,25Lux,Red-200lux	Red,200Lux,Red-200lux	1.0
Red,25Lux,Red-25lux	Clear,200Lux,Red-200lux	0.0653
Red,25Lux,Red-25lux	Clear,200Lux,Red-25lux	0.9122
Red,25Lux,Red-25lux	Clear,25Lux,Clear-200lux	1.0
Red,25Lux,Red-25lux	Clear,25Lux,Red-200lux	1.0
Red,25Lux,Red-25lux	Red,200Lux,Clear-200lux	1.0
Red,25Lux,Red-25lux	Red,200Lux,Clear-25lux	0.9991

Red,25Lux,Red-25lux	Red,200Lux,Red-200lux	1.0
Red,25Lux,Red-25lux	Red,25Lux,Clear-200lux	1.0
Red,25Lux,Red-25lux	Red,25Lux,Red-200lux	1.0
