# Supplementary material for: The Future Is Not Bright: Evaluation of Rat Preferences for Color and Intensity of Light
Source: Animals (Basel). 2024 Jul 12;14(14):2045. doi: 10.3390/ani14142045 (PMC11273897; doi:10.3390/ani14142045)
Supplement: Supplementary file 1 [file animals-14-02045-s001.zip › Table S2 - SAS code for statistical models and accompanying data 20240531.pdf]

Data and statistical script

This document provides both data and statistical script used to analyze results from our project. While the data was analyzed in JMP, the software provides a SAS script output with the data used for the analysis. The data can be easily cut and pasted into other statistical software.

1. Behavioral data and model

```
DATA RatCageColorAnalyses09012023_aid; INPUT  cage Strain &$16. Sex &$16.
Color_Housing &$16. Lighting_Housing &$16. Preference_ Light_Phase &$16.
Color_Preference &$16. Lighting_Preference &$16. BehaviorCategory2 &$16.; Lines;
      CD  Male  Clear  25Lux  1  Dark  Clear  200lux  Active
CD  Male  Clear  25Lux  1  Dark  Clear  200lux  Inactive
CD  Male  Clear  25Lux  1  Dark  Clear  25lux  Active
CD  Male  Clear  25Lux  1  Dark  Clear  25lux  Inactive
CD  Male  Clear  25Lux  1  Dark  Red  200lux  Active
CD  Male  Clear  25Lux  1  Dark  Red  200lux  Inactive
CD  Male  Clear  25Lux  1  Dark  Red  25lux  Active
CD  Male  Clear  25Lux  1  Dark  Red  25lux  Inactive
CD  Male  Clear  25Lux  1  Light  Clear  200lux  Active
CD  Male  Clear  25Lux  1  Light  Clear  200lux  Inactive
CD  Male  Clear  25Lux  1  Light  Clear  25lux  Active
CD  Male  Clear  25Lux  1  Light  Clear  25lux  Inactive
CD  Male  Clear  25Lux  1  Light  Red  200lux  Active
CD  Male  Clear  25Lux  1  Light  Red  200lux  Inactive
CD  Male  Clear  25Lux  1  Light  Red  25lux  Active
CD  Male  Clear  25Lux  1  Light  Red  25lux  Inactive
CD  Male  Clear  25Lux  2  Dark  Clear  200lux  Active
CD  Male  Clear  25Lux  2  Dark  Clear  200lux  Inactive
CD  Male  Clear  25Lux  2  Dark  Clear  25lux  Active
CD  Male  Clear  25Lux  2  Dark  Clear  25lux  Inactive
CD  Male  Clear  25Lux  2  Dark  Red  200lux  Active
CD  Male  Clear  25Lux  2  Dark  Red  200lux  Inactive
CD  Male  Clear  25Lux  2  Dark  Red  25lux  Active
CD  Male  Clear  25Lux  2  Dark  Red  25lux  Inactive
CD  Male  Clear  25Lux  2  Light  Clear  200lux  Active
CD  Male  Clear  25Lux  2  Light  Clear  200lux  Inactive
CD  Male  Clear  25Lux  2  Light  Clear  25lux  Active
CD  Male  Clear  25Lux  2  Light  Clear  25lux  Inactive
CD  Male  Clear  25Lux  2  Light  Red  200lux  Active
CD  Male  Clear  25Lux  2  Light  Red  200lux  Inactive
CD  Male  Clear  25Lux  2  Light  Red  25lux  Active
CD  Male  Clear  25Lux  2  Light  Red  25lux  Inactive
CD  Male  Clear  25Lux  3  Dark  Clear  200lux  Active
CD  Male  Clear  25Lux  3  Dark  Clear  200lux  Inactive
CD  Male  Clear  25Lux  3  Dark  Clear  25lux  Active
CD  Male  Clear  25Lux  3  Dark  Clear  25lux  Inactive
CD  Male  Clear  25Lux  3  Dark  Red  200lux  Active
CD  Male  Clear  25Lux  3  Dark  Red  200lux  Inactive
CD  Male  Clear  25Lux  3  Dark  Red  25lux  Active
CD  Male  Clear  25Lux  3  Dark  Red  25lux  Inactive
```

CD	Male	Clear	25Lux	3	Light	Clear	200lux	Active
CD	Male	Clear	25Lux	3	Light	Clear	200lux	Inactive
CD	Male	Clear	25Lux	3	Light	Clear	25lux	Active
CD	Male	Clear	25Lux	3	Light	Clear	25lux	Inactive
CD	Male	Clear	25Lux	3	Light	Red	200lux	Active
CD	Male	Clear	25Lux	3	Light	Red	200lux	Inactive
CD	Male	Clear	25Lux	3	Light	Red	25lux	Active
CD	Male	Clear	25Lux	3	Light	Red	25lux	Inactive
CD	Male	Clear	25Lux	1	Dark	Clear	200lux	Active
CD	Male	Clear	25Lux	1	Dark	Clear	200lux	Inactive
CD	Male	Clear	25Lux	1	Dark	Clear	25lux	Active
CD	Male	Clear	25Lux	1	Dark	Clear	25lux	Inactive
CD	Male	Clear	25Lux	1	Dark	Red	200lux	Active
CD	Male	Clear	25Lux	1	Dark	Red	200lux	Inactive
CD	Male	Clear	25Lux	1	Dark	Red	25lux	Active
CD	Male	Clear	25Lux	1	Dark	Red	25lux	Inactive
CD	Male	Clear	25Lux	1	Light	Clear	200lux	Active
CD	Male	Clear	25Lux	1	Light	Clear	200lux	Inactive
CD	Male	Clear	25Lux	1	Light	Clear	25lux	Active
CD	Male	Clear	25Lux	1	Light	Clear	25lux	Inactive
CD	Male	Clear	25Lux	1	Light	Red	200lux	Active
CD	Male	Clear	25Lux	1	Light	Red	200lux	Inactive
CD	Male	Clear	25Lux	1	Light	Red	25lux	Active
CD	Male	Clear	25Lux	1	Light	Red	25lux	Inactive
CD	Male	Clear	25Lux	2	Dark	Clear	200lux	Active
CD	Male	Clear	25Lux	2	Dark	Clear	200lux	Inactive
CD	Male	Clear	25Lux	2	Dark	Clear	25lux	Active
CD	Male	Clear	25Lux	2	Dark	Clear	25lux	Inactive
CD	Male	Clear	25Lux	2	Dark	Red	200lux	Active
CD	Male	Clear	25Lux	2	Dark	Red	200lux	Inactive
CD	Male	Clear	25Lux	2	Dark	Red	25lux	Active
CD	Male	Clear	25Lux	2	Dark	Red	25lux	Inactive
CD	Male	Clear	25Lux	2	Light	Clear	200lux	Active
CD	Male	Clear	25Lux	2	Light	Clear	200lux	Inactive
CD	Male	Clear	25Lux	2	Light	Clear	25lux	Active
CD	Male	Clear	25Lux	2	Light	Clear	25lux	Inactive
CD	Male	Clear	25Lux	2	Light	Red	200lux	Active
CD	Male	Clear	25Lux	2	Light	Red	200lux	Inactive
CD	Male	Clear	25Lux	2	Light	Red	25lux	Active
CD	Male	Clear	25Lux	2	Light	Red	25lux	Inactive
CD	Male	Clear	25Lux	3	Dark	Clear	200lux	Active
CD	Male	Clear	25Lux	3	Dark	Clear	200lux	Inactive
CD	Male	Clear	25Lux	3	Dark	Clear	25lux	Active
CD	Male	Clear	25Lux	3	Dark	Clear	25lux	Inactive
CD	Male	Clear	25Lux	3	Dark	Red	200lux	Active
CD	Male	Clear	25Lux	3	Dark	Red	200lux	Inactive
CD	Male	Clear	25Lux	3	Dark	Red	25lux	Active
CD	Male	Clear	25Lux	3	Dark	Red	25lux	Inactive
CD	Male	Clear	25Lux	3	Light	Clear	200lux	Active
CD	Male	Clear	25Lux	3	Light	Clear	200lux	Inactive
CD	Male	Clear	25Lux	3	Light	Clear	25lux	Active
CD	Male	Clear	25Lux	3	Light	Clear	25lux	Inactive
CD	Male	Clear	25Lux	3	Light	Red	200lux	Active
CD	Male	Clear	25Lux	3	Light	Red	200lux	Inactive
CD	Male	Clear	25Lux	3	Light	Red	25lux	Active

CD	Male	Clear	25Lux	3	Light	Red	25lux	Inactive
CD	Female	Clear	25Lux	1	Dark	Clear	200lux	Active
CD	Female	Clear	25Lux	1	Dark	Clear	200lux	Inactive
CD	Female	Clear	25Lux	1	Dark	Clear	25lux	Active
CD	Female	Clear	25Lux	1	Dark	Clear	25lux	Inactive
CD	Female	Clear	25Lux	1	Dark	Red	200lux	Active
CD	Female	Clear	25Lux	1	Dark	Red	200lux	Inactive
CD	Female	Clear	25Lux	1	Dark	Red	25lux	Active
CD	Female	Clear	25Lux	1	Dark	Red	25lux	Inactive
CD	Female	Clear	25Lux	1	Light	Clear	200lux	Active
CD	Female	Clear	25Lux	1	Light	Clear	200lux	Inactive
CD	Female	Clear	25Lux	1	Light	Clear	25lux	Active
CD	Female	Clear	25Lux	1	Light	Clear	25lux	Inactive
CD	Female	Clear	25Lux	1	Light	Red	200lux	Active
CD	Female	Clear	25Lux	1	Light	Red	200lux	Inactive
CD	Female	Clear	25Lux	1	Light	Red	25lux	Active
CD	Female	Clear	25Lux	1	Light	Red	25lux	Inactive
CD	Female	Clear	25Lux	2	Dark	Clear	200lux	Active
CD	Female	Clear	25Lux	2	Dark	Clear	200lux	Inactive
CD	Female	Clear	25Lux	2	Dark	Clear	25lux	Active
CD	Female	Clear	25Lux	2	Dark	Clear	25lux	Inactive
CD	Female	Clear	25Lux	2	Dark	Red	200lux	Active
CD	Female	Clear	25Lux	2	Dark	Red	200lux	Inactive
CD	Female	Clear	25Lux	2	Dark	Red	25lux	Active
CD	Female	Clear	25Lux	2	Dark	Red	25lux	Inactive
CD	Female	Clear	25Lux	2	Light	Clear	200lux	Active
CD	Female	Clear	25Lux	2	Light	Clear	200lux	Inactive
CD	Female	Clear	25Lux	2	Light	Clear	25lux	Active
CD	Female	Clear	25Lux	2	Light	Clear	25lux	Inactive
CD	Female	Clear	25Lux	2	Light	Red	200lux	Active
CD	Female	Clear	25Lux	2	Light	Red	200lux	Inactive
CD	Female	Clear	25Lux	2	Light	Red	25lux	Active
CD	Female	Clear	25Lux	2	Light	Red	25lux	Inactive
CD	Female	Clear	25Lux	3	Dark	Clear	200lux	Active
CD	Female	Clear	25Lux	3	Dark	Clear	200lux	Inactive
CD	Female	Clear	25Lux	3	Dark	Clear	25lux	Active
CD	Female	Clear	25Lux	3	Dark	Clear	25lux	Inactive
CD	Female	Clear	25Lux	3	Dark	Red	200lux	Active
CD	Female	Clear	25Lux	3	Dark	Red	200lux	Inactive
CD	Female	Clear	25Lux	3	Dark	Red	25lux	Active
CD	Female	Clear	25Lux	3	Dark	Red	25lux	Inactive
CD	Female	Clear	25Lux	3	Light	Clear	200lux	Active
CD	Female	Clear	25Lux	3	Light	Clear	200lux	Inactive
CD	Female	Clear	25Lux	3	Light	Clear	25lux	Active
CD	Female	Clear	25Lux	3	Light	Clear	25lux	Inactive
CD	Female	Clear	25Lux	3	Light	Red	200lux	Active
CD	Female	Clear	25Lux	3	Light	Red	200lux	Inactive
CD	Female	Clear	25Lux	3	Light	Red	25lux	Active
CD	Female	Clear	25Lux	3	Light	Red	25lux	Inactive
CD	Female	Clear	25Lux	1	Dark	Clear	200lux	Active
CD	Female	Clear	25Lux	1	Dark	Clear	200lux	Inactive
CD	Female	Clear	25Lux	1	Dark	Clear	25lux	Active
CD	Female	Clear	25Lux	1	Dark	Clear	25lux	Inactive
CD	Female	Clear	25Lux	1	Dark	Red	200lux	Active
CD	Female	Clear	25Lux	1	Dark	Red	200lux	Inactive

CD Female Clear 25Lux 1 Dark Red 25lux Active
CD Female Clear 25Lux 1 Dark Red 25lux Inactive
CD Female Clear 25Lux 1 Light Clear 200lux Active
CD Female Clear 25Lux 1 Light Clear 200lux Inactive
CD Female Clear 25Lux 1 Light Clear 25lux Active
CD Female Clear 25Lux 1 Light Clear 25lux Inactive
CD Female Clear 25Lux 1 Light Red 200lux Active
CD Female Clear 25Lux 1 Light Red 200lux Inactive
CD Female Clear 25Lux 1 Light Red 25lux Active
CD Female Clear 25Lux 1 Light Red 25lux Inactive
CD Female Clear 25Lux 2 Dark Clear 200lux Active
CD Female Clear 25Lux 2 Dark Clear 200lux Inactive
CD Female Clear 25Lux 2 Dark Clear 25lux Active
CD Female Clear 25Lux 2 Dark Clear 25lux Inactive
CD Female Clear 25Lux 2 Dark Red 200lux Active
CD Female Clear 25Lux 2 Dark Red 200lux Inactive
CD Female Clear 25Lux 2 Dark Red 25lux Active
CD Female Clear 25Lux 2 Dark Red 25lux Inactive
CD Female Clear 25Lux 2 Light Clear 200lux Active
CD Female Clear 25Lux 2 Light Clear 200lux Inactive
CD Female Clear 25Lux 2 Light Clear 25lux Active
CD Female Clear 25Lux 2 Light Clear 25lux Inactive
CD Female Clear 25Lux 2 Light Red 200lux Active
CD Female Clear 25Lux 2 Light Red 200lux Inactive
CD Female Clear 25Lux 2 Light Red 25lux Active
CD Female Clear 25Lux 2 Light Red 25lux Inactive
4 CD Female Clear 25Lux 3 Dark Clear 200lux Active
4 CD Female Clear 25Lux 3 Dark Clear 200lux Inactive
4 CD Female Clear 25Lux 3 Dark Clear 25lux Active
4 CD Female Clear 25Lux 3 Dark Clear 25lux Inactive
4 CD Female Clear 25Lux 3 Dark Red 200lux Active
4 CD Female Clear 25Lux 3 Dark Red 200lux Inactive
4 CD Female Clear 25Lux 3 Dark Red 25lux Active
4 CD Female Clear 25Lux 3 Dark Red 25lux Inactive
4 CD Female Clear 25Lux 3 Light Clear 200lux Active
4 CD Female Clear 25Lux 3 Light Clear 200lux Inactive
4 CD Female Clear 25Lux 3 Light Clear 25lux Active
4 CD Female Clear 25Lux 3 Light Clear 25lux Inactive
4 CD Female Clear 25Lux 3 Light Red 200lux Active
4 CD Female Clear 25Lux 3 Light Red 200lux Inactive
4 CD Female Clear 25Lux 3 Light Red 25lux Active
4 CD Female Clear 25Lux 3 Light Red 25lux Inactive
CD Male Red 25Lux 1 Dark Clear 200lux Active
CD Male Red 25Lux 1 Dark Clear 200lux Inactive
CD Male Red 25Lux 1 Dark Clear 25lux Active
CD Male Red 25Lux 1 Dark Clear 25lux Inactive
CD Male Red 25Lux 1 Dark Red 200lux Active
CD Male Red 25Lux 1 Dark Red 200lux Inactive
CD Male Red 25Lux 1 Dark Red 25lux Active
CD Male Red 25Lux 1 Dark Red 25lux Inactive
CD Male Red 25Lux 1 Light Clear 200lux Active
CD Male Red 25Lux 1 Light Clear 200lux Inactive
CD Male Red 25Lux 1 Light Clear 25lux Active
CD Male Red 25Lux 1 Light Clear 25lux Inactive
CD Male Red 25Lux 1 Light Red 200lux Active

CD Male Red 25Lux 1 Light Red 200lux Inactive
CD Male Red 25Lux 1 Light Red 25lux Active
CD Male Red 25Lux 1 Light Red 25lux Inactive
CD Male Red 25Lux 2 Dark Clear 200lux Active
CD Male Red 25Lux 2 Dark Clear 200lux Inactive
CD Male Red 25Lux 2 Dark Clear 25lux Active
CD Male Red 25Lux 2 Dark Clear 25lux Inactive
CD Male Red 25Lux 2 Dark Red 200lux Active
CD Male Red 25Lux 2 Dark Red 200lux Inactive
CD Male Red 25Lux 2 Dark Red 25lux Active
CD Male Red 25Lux 2 Dark Red 25lux Inactive
CD Male Red 25Lux 2 Light Clear 200lux Active
CD Male Red 25Lux 2 Light Clear 200lux Inactive
CD Male Red 25Lux 2 Light Clear 25lux Active
CD Male Red 25Lux 2 Light Clear 25lux Inactive
CD Male Red 25Lux 2 Light Red 200lux Active
CD Male Red 25Lux 2 Light Red 200lux Inactive
CD Male Red 25Lux 2 Light Red 25lux Active
CD Male Red 25Lux 2 Light Red 25lux Inactive
5 CD Male Red 25Lux 3 Dark Clear 200lux Active
5 CD Male Red 25Lux 3 Dark Clear 200lux Inactive
5 CD Male Red 25Lux 3 Dark Clear 25lux Active
5 CD Male Red 25Lux 3 Dark Clear 25lux Inactive
5 CD Male Red 25Lux 3 Dark Red 200lux Active
5 CD Male Red 25Lux 3 Dark Red 200lux Inactive
5 CD Male Red 25Lux 3 Dark Red 25lux Active
5 CD Male Red 25Lux 3 Dark Red 25lux Inactive
5 CD Male Red 25Lux 3 Light Clear 200lux Active
5 CD Male Red 25Lux 3 Light Clear 200lux Inactive
5 CD Male Red 25Lux 3 Light Clear 25lux Active
5 CD Male Red 25Lux 3 Light Clear 25lux Inactive
5 CD Male Red 25Lux 3 Light Red 200lux Active
5 CD Male Red 25Lux 3 Light Red 200lux Inactive
5 CD Male Red 25Lux 3 Light Red 25lux Active
5 CD Male Red 25Lux 3 Light Red 25lux Inactive
CD Male Red 25Lux 1 Dark Clear 200lux Active
CD Male Red 25Lux 1 Dark Clear 200lux Inactive
CD Male Red 25Lux 1 Dark Clear 25lux Active
CD Male Red 25Lux 1 Dark Clear 25lux Inactive
CD Male Red 25Lux 1 Dark Red 200lux Active
CD Male Red 25Lux 1 Dark Red 200lux Inactive
CD Male Red 25Lux 1 Dark Red 25lux Active
CD Male Red 25Lux 1 Dark Red 25lux Inactive
CD Male Red 25Lux 1 Light Clear 200lux Active
CD Male Red 25Lux 1 Light Clear 200lux Inactive
CD Male Red 25Lux 1 Light Clear 25lux Active
CD Male Red 25Lux 1 Light Clear 25lux Inactive
CD Male Red 25Lux 1 Light Red 200lux Active
CD Male Red 25Lux 1 Light Red 200lux Inactive
CD Male Red 25Lux 1 Light Red 25lux Active
CD Male Red 25Lux 1 Light Red 25lux Inactive
CD Male Red 25Lux 2 Dark Clear 200lux Active
CD Male Red 25Lux 2 Dark Clear 200lux Inactive
CD Male Red 25Lux 2 Dark Clear 25lux Active
CD Male Red 25Lux 2 Dark Clear 25lux Inactive

CD Male Red 25Lux 2 Dark Red 200lux Active
CD Male Red 25Lux 2 Dark Red 200lux Inactive
CD Male Red 25Lux 2 Dark Red 25lux Active
CD Male Red 25Lux 2 Dark Red 25lux Inactive
CD Male Red 25Lux 2 Light Clear 200lux Active
CD Male Red 25Lux 2 Light Clear 200lux Inactive
CD Male Red 25Lux 2 Light Clear 25lux Active
CD Male Red 25Lux 2 Light Clear 25lux Inactive
CD Male Red 25Lux 2 Light Red 200lux Active
CD Male Red 25Lux 2 Light Red 200lux Inactive
CD Male Red 25Lux 2 Light Red 25lux Active
CD Male Red 25Lux 2 Light Red 25lux Inactive
6 CD Male Red 25Lux 3 Dark Clear 200lux Active
6 CD Male Red 25Lux 3 Dark Clear 200lux Inactive
6 CD Male Red 25Lux 3 Dark Clear 25lux Active
6 CD Male Red 25Lux 3 Dark Clear 25lux Inactive
6 CD Male Red 25Lux 3 Dark Red 200lux Active
6 CD Male Red 25Lux 3 Dark Red 200lux Inactive
6 CD Male Red 25Lux 3 Dark Red 25lux Active
6 CD Male Red 25Lux 3 Dark Red 25lux Inactive
6 CD Male Red 25Lux 3 Light Clear 200lux Active
6 CD Male Red 25Lux 3 Light Clear 200lux Inactive
6 CD Male Red 25Lux 3 Light Clear 25lux Active
6 CD Male Red 25Lux 3 Light Clear 25lux Inactive
6 CD Male Red 25Lux 3 Light Red 200lux Active
6 CD Male Red 25Lux 3 Light Red 200lux Inactive
6 CD Male Red 25Lux 3 Light Red 25lux Active
6 CD Male Red 25Lux 3 Light Red 25lux Inactive
CD Female Red 25Lux 1 Dark Clear 200lux Active
CD Female Red 25Lux 1 Dark Clear 200lux Inactive
CD Female Red 25Lux 1 Dark Clear 25lux Active
CD Female Red 25Lux 1 Dark Clear 25lux Inactive
CD Female Red 25Lux 1 Dark Red 200lux Active
CD Female Red 25Lux 1 Dark Red 200lux Inactive
CD Female Red 25Lux 1 Dark Red 25lux Active
CD Female Red 25Lux 1 Dark Red 25lux Inactive
CD Female Red 25Lux 1 Light Clear 200lux Active
CD Female Red 25Lux 1 Light Clear 200lux Inactive
CD Female Red 25Lux 1 Light Clear 25lux Active
CD Female Red 25Lux 1 Light Clear 25lux Inactive
CD Female Red 25Lux 1 Light Red 200lux Active
CD Female Red 25Lux 1 Light Red 200lux Inactive
CD Female Red 25Lux 1 Light Red 25lux Active
CD Female Red 25Lux 1 Light Red 25lux Inactive
CD Female Red 25Lux 2 Dark Clear 200lux Active
CD Female Red 25Lux 2 Dark Clear 200lux Inactive
CD Female Red 25Lux 2 Dark Clear 25lux Active
CD Female Red 25Lux 2 Dark Clear 25lux Inactive
CD Female Red 25Lux 2 Dark Red 200lux Active
CD Female Red 25Lux 2 Dark Red 200lux Inactive
CD Female Red 25Lux 2 Dark Red 25lux Active
CD Female Red 25Lux 2 Dark Red 25lux Inactive
CD Female Red 25Lux 2 Light Clear 200lux Active
CD Female Red 25Lux 2 Light Clear 200lux Inactive
CD Female Red 25Lux 2 Light Clear 25lux Active

CD Female Red 25Lux 2 Light Clear 25lux Inactive
CD Female Red 25Lux 2 Light Red 200lux Active
CD Female Red 25Lux 2 Light Red 200lux Inactive
CD Female Red 25Lux 2 Light Red 25lux Active
CD Female Red 25Lux 2 Light Red 25lux Inactive
7 CD Female Red 25Lux 3 Dark Clear 200lux Active
7 CD Female Red 25Lux 3 Dark Clear 200lux Inactive
7 CD Female Red 25Lux 3 Dark Clear 25lux Active
7 CD Female Red 25Lux 3 Dark Clear 25lux Inactive
7 CD Female Red 25Lux 3 Dark Red 200lux Active
7 CD Female Red 25Lux 3 Dark Red 200lux Inactive
7 CD Female Red 25Lux 3 Dark Red 25lux Active
7 CD Female Red 25Lux 3 Dark Red 25lux Inactive
7 CD Female Red 25Lux 3 Light Clear 200lux Active
7 CD Female Red 25Lux 3 Light Clear 200lux Inactive
7 CD Female Red 25Lux 3 Light Clear 25lux Active
7 CD Female Red 25Lux 3 Light Clear 25lux Inactive
7 CD Female Red 25Lux 3 Light Red 200lux Active
7 CD Female Red 25Lux 3 Light Red 200lux Inactive
7 CD Female Red 25Lux 3 Light Red 25lux Active
7 CD Female Red 25Lux 3 Light Red 25lux Inactive
CD Female Red 25Lux 1 Dark Clear 200lux Active
CD Female Red 25Lux 1 Dark Clear 200lux Inactive
CD Female Red 25Lux 1 Dark Clear 25lux Active
CD Female Red 25Lux 1 Dark Clear 25lux Inactive
CD Female Red 25Lux 1 Dark Red 200lux Active
CD Female Red 25Lux 1 Dark Red 200lux Inactive
CD Female Red 25Lux 1 Dark Red 25lux Active
CD Female Red 25Lux 1 Dark Red 25lux Inactive
CD Female Red 25Lux 1 Light Clear 200lux Active
CD Female Red 25Lux 1 Light Clear 200lux Inactive
CD Female Red 25Lux 1 Light Clear 25lux Active
CD Female Red 25Lux 1 Light Clear 25lux Inactive
CD Female Red 25Lux 1 Light Red 200lux Active
CD Female Red 25Lux 1 Light Red 200lux Inactive
CD Female Red 25Lux 1 Light Red 25lux Active
CD Female Red 25Lux 1 Light Red 25lux Inactive
CD Female Red 25Lux 2 Dark Clear 200lux Active
CD Female Red 25Lux 2 Dark Clear 200lux Inactive
CD Female Red 25Lux 2 Dark Clear 25lux Active
CD Female Red 25Lux 2 Dark Clear 25lux Inactive
CD Female Red 25Lux 2 Dark Red 200lux Active
CD Female Red 25Lux 2 Dark Red 200lux Inactive
CD Female Red 25Lux 2 Dark Red 25lux Active
CD Female Red 25Lux 2 Dark Red 25lux Inactive
CD Female Red 25Lux 2 Light Clear 200lux Active
CD Female Red 25Lux 2 Light Clear 200lux Inactive
CD Female Red 25Lux 2 Light Clear 25lux Active
CD Female Red 25Lux 2 Light Clear 25lux Inactive
CD Female Red 25Lux 2 Light Red 200lux Active
CD Female Red 25Lux 2 Light Red 200lux Inactive
CD Female Red 25Lux 2 Light Red 25lux Active
CD Female Red 25Lux 2 Light Red 25lux Inactive
8 CD Female Red 25Lux 3 Dark Clear 200lux Active
8 CD Female Red 25Lux 3 Dark Clear 200lux Inactive

8 CD Female Red 25Lux 3 Dark Clear 25lux Active
8 CD Female Red 25Lux 3 Dark Clear 25lux Inactive
8 CD Female Red 25Lux 3 Dark Red 200lux Active
8 CD Female Red 25Lux 3 Dark Red 200lux Inactive
8 CD Female Red 25Lux 3 Dark Red 25lux Active
8 CD Female Red 25Lux 3 Dark Red 25lux Inactive
8 CD Female Red 25Lux 3 Light Clear 200lux Active
8 CD Female Red 25Lux 3 Light Clear 200lux Inactive
8 CD Female Red 25Lux 3 Light Clear 25lux Active
8 CD Female Red 25Lux 3 Light Clear 25lux Inactive
8 CD Female Red 25Lux 3 Light Red 200lux Active
8 CD Female Red 25Lux 3 Light Red 200lux Inactive
8 CD Female Red 25Lux 3 Light Red 25lux Active
8 CD Female Red 25Lux 3 Light Red 25lux Inactive
CD Male Clear 200Lux 1 Dark Clear 200lux Active
CD Male Clear 200Lux 1 Dark Clear 200lux Inactive
CD Male Clear 200Lux 1 Dark Clear 25lux Active
CD Male Clear 200Lux 1 Dark Clear 25lux Inactive
CD Male Clear 200Lux 1 Dark Red 200lux Active
CD Male Clear 200Lux 1 Dark Red 200lux Inactive
CD Male Clear 200Lux 1 Dark Red 25lux Active
CD Male Clear 200Lux 1 Dark Red 25lux Inactive
CD Male Clear 200Lux 1 Light Clear 200lux Active
CD Male Clear 200Lux 1 Light Clear 200lux Inactive
CD Male Clear 200Lux 1 Light Clear 25lux Active
CD Male Clear 200Lux 1 Light Clear 25lux Inactive
CD Male Clear 200Lux 1 Light Red 200lux Active
CD Male Clear 200Lux 1 Light Red 200lux Inactive
CD Male Clear 200Lux 1 Light Red 25lux Active
CD Male Clear 200Lux 1 Light Red 25lux Inactive
CD Male Clear 200Lux 2 Dark Clear 200lux Active
CD Male Clear 200Lux 2 Dark Clear 200lux Inactive
CD Male Clear 200Lux 2 Dark Clear 25lux Active
CD Male Clear 200Lux 2 Dark Clear 25lux Inactive
CD Male Clear 200Lux 2 Dark Red 200lux Active
CD Male Clear 200Lux 2 Dark Red 200lux Inactive
CD Male Clear 200Lux 2 Dark Red 25lux Active
CD Male Clear 200Lux 2 Dark Red 25lux Inactive
CD Male Clear 200Lux 2 Light Clear 200lux Active
CD Male Clear 200Lux 2 Light Clear 200lux Inactive
CD Male Clear 200Lux 2 Light Clear 25lux Active
CD Male Clear 200Lux 2 Light Clear 25lux Inactive
CD Male Clear 200Lux 2 Light Red 200lux Active
CD Male Clear 200Lux 2 Light Red 200lux Inactive
CD Male Clear 200Lux 2 Light Red 25lux Active
CD Male Clear 200Lux 2 Light Red 25lux Inactive
9 CD Male Clear 200Lux 3 Dark Clear 200lux Active
9 CD Male Clear 200Lux 3 Dark Clear 200lux Inactive
9 CD Male Clear 200Lux 3 Dark Clear 25lux Active
9 CD Male Clear 200Lux 3 Dark Clear 25lux Inactive
9 CD Male Clear 200Lux 3 Dark Red 200lux Active
9 CD Male Clear 200Lux 3 Dark Red 200lux Inactive
9 CD Male Clear 200Lux 3 Dark Red 25lux Active
9 CD Male Clear 200Lux 3 Dark Red 25lux Inactive
9 CD Male Clear 200Lux 3 Light Clear 200lux Active

9 CD Male Clear 200Lux 3 Light Clear 200lux Inactive
9 CD Male Clear 200Lux 3 Light Clear 25lux Active
9 CD Male Clear 200Lux 3 Light Clear 25lux Inactive
9 CD Male Clear 200Lux 3 Light Red 200lux Active
9 CD Male Clear 200Lux 3 Light Red 200lux Inactive
9 CD Male Clear 200Lux 3 Light Red 25lux Active
9 CD Male Clear 200Lux 3 Light Red 25lux Inactive
CD Female Clear 200Lux 1 Dark Clear 200lux Active
CD Female Clear 200Lux 1 Dark Clear 200lux Inactive
CD Female Clear 200Lux 1 Dark Clear 25lux Active
CD Female Clear 200Lux 1 Dark Clear 25lux Inactive
CD Female Clear 200Lux 1 Dark Red 200lux Active
CD Female Clear 200Lux 1 Dark Red 200lux Inactive
CD Female Clear 200Lux 1 Dark Red 25lux Active
CD Female Clear 200Lux 1 Dark Red 25lux Inactive
CD Female Clear 200Lux 1 Light Clear 200lux Active
CD Female Clear 200Lux 1 Light Clear 200lux Inactive
CD Female Clear 200Lux 1 Light Clear 25lux Active
CD Female Clear 200Lux 1 Light Clear 25lux Inactive
CD Female Clear 200Lux 1 Light Red 200lux Active
CD Female Clear 200Lux 1 Light Red 200lux Inactive
CD Female Clear 200Lux 1 Light Red 25lux Active
CD Female Clear 200Lux 1 Light Red 25lux Inactive
CD Female Clear 200Lux 2 Dark Clear 200lux Active
CD Female Clear 200Lux 2 Dark Clear 200lux Inactive
CD Female Clear 200Lux 2 Dark Clear 25lux Active
CD Female Clear 200Lux 2 Dark Clear 25lux Inactive
CD Female Clear 200Lux 2 Dark Red 200lux Active
CD Female Clear 200Lux 2 Dark Red 200lux Inactive
CD Female Clear 200Lux 2 Dark Red 25lux Active
CD Female Clear 200Lux 2 Dark Red 25lux Inactive
CD Female Clear 200Lux 2 Light Clear 200lux Active
CD Female Clear 200Lux 2 Light Clear 200lux Inactive
CD Female Clear 200Lux 2 Light Clear 25lux Active
CD Female Clear 200Lux 2 Light Clear 25lux Inactive
CD Female Clear 200Lux 2 Light Red 200lux Active
CD Female Clear 200Lux 2 Light Red 200lux Inactive
CD Female Clear 200Lux 2 Light Red 25lux Active
CD Female Clear 200Lux 2 Light Red 25lux Inactive
10 CD Female Clear 200Lux 3 Dark Clear 200lux Active
10 CD Female Clear 200Lux 3 Dark Clear 200lux Inactive
10 CD Female Clear 200Lux 3 Dark Clear 25lux Active
10 CD Female Clear 200Lux 3 Dark Clear 25lux Inactive
10 CD Female Clear 200Lux 3 Dark Red 200lux Active
10 CD Female Clear 200Lux 3 Dark Red 200lux Inactive
10 CD Female Clear 200Lux 3 Dark Red 25lux Active
10 CD Female Clear 200Lux 3 Dark Red 25lux Inactive
10 CD Female Clear 200Lux 3 Light Clear 200lux Active
10 CD Female Clear 200Lux 3 Light Clear 200lux Inactive
10 CD Female Clear 200Lux 3 Light Clear 25lux Active
10 CD Female Clear 200Lux 3 Light Clear 25lux Inactive
10 CD Female Clear 200Lux 3 Light Red 200lux Active
10 CD Female Clear 200Lux 3 Light Red 200lux Inactive
10 CD Female Clear 200Lux 3 Light Red 25lux Active
10 CD Female Clear 200Lux 3 Light Red 25lux Inactive

CD Male Red 200Lux 1 Dark Clear 200lux Active
CD Male Red 200Lux 1 Dark Clear 200lux Inactive
CD Male Red 200Lux 1 Dark Clear 25lux Active
CD Male Red 200Lux 1 Dark Clear 25lux Inactive
CD Male Red 200Lux 1 Dark Red 200lux Active
CD Male Red 200Lux 1 Dark Red 200lux Inactive
CD Male Red 200Lux 1 Dark Red 25lux Active
CD Male Red 200Lux 1 Dark Red 25lux Inactive
CD Male Red 200Lux 1 Light Clear 200lux Active
CD Male Red 200Lux 1 Light Clear 200lux Inactive
CD Male Red 200Lux 1 Light Clear 25lux Active
CD Male Red 200Lux 1 Light Clear 25lux Inactive
CD Male Red 200Lux 1 Light Red 200lux Active
CD Male Red 200Lux 1 Light Red 200lux Inactive
CD Male Red 200Lux 1 Light Red 25lux Active
CD Male Red 200Lux 1 Light Red 25lux Inactive
CD Male Red 200Lux 2 Dark Clear 200lux Active
CD Male Red 200Lux 2 Dark Clear 200lux Inactive
CD Male Red 200Lux 2 Dark Clear 25lux Active
CD Male Red 200Lux 2 Dark Clear 25lux Inactive
CD Male Red 200Lux 2 Dark Red 200lux Active
CD Male Red 200Lux 2 Dark Red 200lux Inactive
CD Male Red 200Lux 2 Dark Red 25lux Active
CD Male Red 200Lux 2 Dark Red 25lux Inactive
CD Male Red 200Lux 2 Light Clear 200lux Active
CD Male Red 200Lux 2 Light Clear 200lux Inactive
CD Male Red 200Lux 2 Light Clear 25lux Active
CD Male Red 200Lux 2 Light Clear 25lux Inactive
CD Male Red 200Lux 2 Light Red 200lux Active
CD Male Red 200Lux 2 Light Red 200lux Inactive
CD Male Red 200Lux 2 Light Red 25lux Active
CD Male Red 200Lux 2 Light Red 25lux Inactive
11 CD Male Red 200Lux 3 Dark Clear 200lux Active
11 CD Male Red 200Lux 3 Dark Clear 200lux Inactive
11 CD Male Red 200Lux 3 Dark Clear 25lux Active
11 CD Male Red 200Lux 3 Dark Clear 25lux Inactive
11 CD Male Red 200Lux 3 Dark Red 200lux Active
11 CD Male Red 200Lux 3 Dark Red 200lux Inactive
11 CD Male Red 200Lux 3 Dark Red 25lux Active
11 CD Male Red 200Lux 3 Dark Red 25lux Inactive
11 CD Male Red 200Lux 3 Light Clear 200lux Active
11 CD Male Red 200Lux 3 Light Clear 200lux Inactive
11 CD Male Red 200Lux 3 Light Clear 25lux Active
11 CD Male Red 200Lux 3 Light Clear 25lux Inactive
11 CD Male Red 200Lux 3 Light Red 200lux Active
11 CD Male Red 200Lux 3 Light Red 200lux Inactive
11 CD Male Red 200Lux 3 Light Red 25lux Active
11 CD Male Red 200Lux 3 Light Red 25lux Inactive
CD Female Red 200Lux 1 Dark Clear 200lux Active
CD Female Red 200Lux 1 Dark Clear 200lux Inactive
CD Female Red 200Lux 1 Dark Clear 25lux Active
CD Female Red 200Lux 1 Dark Clear 25lux Inactive
CD Female Red 200Lux 1 Dark Red 200lux Active
CD Female Red 200Lux 1 Dark Red 200lux Inactive
CD Female Red 200Lux 1 Dark Red 25lux Active

CD Female Red 200Lux 1 Dark Red 25lux Inactive
CD Female Red 200Lux 1 Light Clear 200lux Active
CD Female Red 200Lux 1 Light Clear 200lux Inactive
CD Female Red 200Lux 1 Light Clear 25lux Active
CD Female Red 200Lux 1 Light Clear 25lux Inactive
CD Female Red 200Lux 1 Light Red 200lux Active
CD Female Red 200Lux 1 Light Red 200lux Inactive
CD Female Red 200Lux 1 Light Red 25lux Active
CD Female Red 200Lux 1 Light Red 25lux Inactive
CD Female Red 200Lux 2 Dark Clear 200lux Active
CD Female Red 200Lux 2 Dark Clear 200lux Inactive
CD Female Red 200Lux 2 Dark Clear 25lux Active
CD Female Red 200Lux 2 Dark Clear 25lux Inactive
CD Female Red 200Lux 2 Dark Red 200lux Active
CD Female Red 200Lux 2 Dark Red 200lux Inactive
CD Female Red 200Lux 2 Dark Red 25lux Active
CD Female Red 200Lux 2 Dark Red 25lux Inactive
CD Female Red 200Lux 2 Light Clear 200lux Active
CD Female Red 200Lux 2 Light Clear 200lux Inactive
CD Female Red 200Lux 2 Light Clear 25lux Active
CD Female Red 200Lux 2 Light Clear 25lux Inactive
CD Female Red 200Lux 2 Light Red 200lux Active
CD Female Red 200Lux 2 Light Red 200lux Inactive
CD Female Red 200Lux 2 Light Red 25lux Active
CD Female Red 200Lux 2 Light Red 25lux Inactive
12 CD Female Red 200Lux 3 Dark Clear 200lux Active
12 CD Female Red 200Lux 3 Dark Clear 200lux Inactive
12 CD Female Red 200Lux 3 Dark Clear 25lux Active
12 CD Female Red 200Lux 3 Dark Clear 25lux Inactive
12 CD Female Red 200Lux 3 Dark Red 200lux Active
12 CD Female Red 200Lux 3 Dark Red 200lux Inactive
12 CD Female Red 200Lux 3 Dark Red 25lux Active
12 CD Female Red 200Lux 3 Dark Red 25lux Inactive
12 CD Female Red 200Lux 3 Light Clear 200lux Active
12 CD Female Red 200Lux 3 Light Clear 200lux Inactive
12 CD Female Red 200Lux 3 Light Clear 25lux Active
12 CD Female Red 200Lux 3 Light Clear 25lux Inactive
12 CD Female Red 200Lux 3 Light Red 200lux Active
12 CD Female Red 200Lux 3 Light Red 200lux Inactive
12 CD Female Red 200Lux 3 Light Red 25lux Active
12 CD Female Red 200Lux 3 Light Red 25lux Inactive
CD Male Clear 200Lux 1 Dark Clear 200lux Active
CD Male Clear 200Lux 1 Dark Clear 200lux Inactive
CD Male Clear 200Lux 1 Dark Clear 25lux Active
CD Male Clear 200Lux 1 Dark Clear 25lux Inactive
CD Male Clear 200Lux 1 Dark Red 200lux Active
CD Male Clear 200Lux 1 Dark Red 200lux Inactive
CD Male Clear 200Lux 1 Dark Red 25lux Active
CD Male Clear 200Lux 1 Dark Red 25lux Inactive
CD Male Clear 200Lux 1 Light Clear 200lux Active
CD Male Clear 200Lux 1 Light Clear 200lux Inactive
CD Male Clear 200Lux 1 Light Clear 25lux Active
CD Male Clear 200Lux 1 Light Clear 25lux Inactive
CD Male Clear 200Lux 1 Light Red 200lux Active
CD Male Clear 200Lux 1 Light Red 200lux Inactive

CD Male Clear 200Lux 1 Light Red 25lux Active
CD Male Clear 200Lux 1 Light Red 25lux Inactive
CD Male Clear 200Lux 2 Dark Clear 200lux Active
CD Male Clear 200Lux 2 Dark Clear 200lux Inactive
CD Male Clear 200Lux 2 Dark Clear 25lux Active
CD Male Clear 200Lux 2 Dark Clear 25lux Inactive
CD Male Clear 200Lux 2 Dark Red 200lux Active
CD Male Clear 200Lux 2 Dark Red 200lux Inactive
CD Male Clear 200Lux 2 Dark Red 25lux Active
CD Male Clear 200Lux 2 Dark Red 25lux Inactive
CD Male Clear 200Lux 2 Light Clear 200lux Active
CD Male Clear 200Lux 2 Light Clear 200lux Inactive
CD Male Clear 200Lux 2 Light Clear 25lux Active
CD Male Clear 200Lux 2 Light Clear 25lux Inactive
CD Male Clear 200Lux 2 Light Red 200lux Active
CD Male Clear 200Lux 2 Light Red 200lux Inactive
CD Male Clear 200Lux 2 Light Red 25lux Active
CD Male Clear 200Lux 2 Light Red 25lux Inactive
13 CD Male Clear 200Lux 3 Dark Clear 200lux Active
13 CD Male Clear 200Lux 3 Dark Clear 200lux Inactive
13 CD Male Clear 200Lux 3 Dark Clear 25lux Active
13 CD Male Clear 200Lux 3 Dark Clear 25lux Inactive
13 CD Male Clear 200Lux 3 Dark Red 200lux Active
13 CD Male Clear 200Lux 3 Dark Red 200lux Inactive
13 CD Male Clear 200Lux 3 Dark Red 25lux Active
13 CD Male Clear 200Lux 3 Dark Red 25lux Inactive
13 CD Male Clear 200Lux 3 Light Clear 200lux Active
13 CD Male Clear 200Lux 3 Light Clear 200lux Inactive
13 CD Male Clear 200Lux 3 Light Clear 25lux Active
13 CD Male Clear 200Lux 3 Light Clear 25lux Inactive
13 CD Male Clear 200Lux 3 Light Red 200lux Active
13 CD Male Clear 200Lux 3 Light Red 200lux Inactive
13 CD Male Clear 200Lux 3 Light Red 25lux Active
13 CD Male Clear 200Lux 3 Light Red 25lux Inactive
CD Male Clear 200Lux 1 Dark Clear 200lux Active
CD Male Clear 200Lux 1 Dark Clear 200lux Inactive
CD Male Clear 200Lux 1 Dark Clear 25lux Active
CD Male Clear 200Lux 1 Dark Clear 25lux Inactive
CD Male Clear 200Lux 1 Dark Red 200lux Active
CD Male Clear 200Lux 1 Dark Red 200lux Inactive
CD Male Clear 200Lux 1 Dark Red 25lux Active
CD Male Clear 200Lux 1 Dark Red 25lux Inactive
CD Male Clear 200Lux 1 Light Clear 200lux Active
CD Male Clear 200Lux 1 Light Clear 200lux Inactive
CD Male Clear 200Lux 1 Light Clear 25lux Active
CD Male Clear 200Lux 1 Light Clear 25lux Inactive
CD Male Clear 200Lux 1 Light Red 200lux Active
CD Male Clear 200Lux 1 Light Red 200lux Inactive
CD Male Clear 200Lux 1 Light Red 25lux Active
CD Male Clear 200Lux 1 Light Red 25lux Inactive
CD Male Clear 200Lux 2 Dark Clear 200lux Active
CD Male Clear 200Lux 2 Dark Clear 200lux Inactive
CD Male Clear 200Lux 2 Dark Clear 25lux Active
CD Male Clear 200Lux 2 Dark Clear 25lux Inactive
CD Male Clear 200Lux 2 Dark Red 200lux Active

CD Male Clear 200Lux 2 Dark Red 200lux Inactive
CD Male Clear 200Lux 2 Dark Red 25lux Active
CD Male Clear 200Lux 2 Dark Red 25lux Inactive
CD Male Clear 200Lux 2 Light Clear 200lux Active
CD Male Clear 200Lux 2 Light Clear 200lux Inactive
CD Male Clear 200Lux 2 Light Clear 25lux Active
CD Male Clear 200Lux 2 Light Clear 25lux Inactive
CD Male Clear 200Lux 2 Light Red 200lux Active
CD Male Clear 200Lux 2 Light Red 200lux Inactive
CD Male Clear 200Lux 2 Light Red 25lux Active
CD Male Clear 200Lux 2 Light Red 25lux Inactive
14 CD Male Clear 200Lux 3 Dark Clear 200lux Active
14 CD Male Clear 200Lux 3 Dark Clear 200lux Inactive
14 CD Male Clear 200Lux 3 Dark Clear 25lux Active
14 CD Male Clear 200Lux 3 Dark Clear 25lux Inactive
14 CD Male Clear 200Lux 3 Dark Red 200lux Active
14 CD Male Clear 200Lux 3 Dark Red 200lux Inactive
14 CD Male Clear 200Lux 3 Dark Red 25lux Active
14 CD Male Clear 200Lux 3 Dark Red 25lux Inactive
14 CD Male Clear 200Lux 3 Light Clear 200lux Active
14 CD Male Clear 200Lux 3 Light Clear 200lux Inactive
14 CD Male Clear 200Lux 3 Light Clear 25lux Active
14 CD Male Clear 200Lux 3 Light Clear 25lux Inactive
14 CD Male Clear 200Lux 3 Light Red 200lux Active
14 CD Male Clear 200Lux 3 Light Red 200lux Inactive
14 CD Male Clear 200Lux 3 Light Red 25lux Active
14 CD Male Clear 200Lux 3 Light Red 25lux Inactive
CD Female Clear 200Lux 1 Dark Clear 200lux Active
CD Female Clear 200Lux 1 Dark Clear 200lux Inactive
CD Female Clear 200Lux 1 Dark Clear 25lux Active
CD Female Clear 200Lux 1 Dark Clear 25lux Inactive
CD Female Clear 200Lux 1 Dark Red 200lux Active
CD Female Clear 200Lux 1 Dark Red 200lux Inactive
CD Female Clear 200Lux 1 Dark Red 25lux Active
CD Female Clear 200Lux 1 Dark Red 25lux Inactive
CD Female Clear 200Lux 1 Light Clear 200lux Active
CD Female Clear 200Lux 1 Light Clear 200lux Inactive
CD Female Clear 200Lux 1 Light Clear 25lux Active
CD Female Clear 200Lux 1 Light Clear 25lux Inactive
CD Female Clear 200Lux 1 Light Red 200lux Active
CD Female Clear 200Lux 1 Light Red 200lux Inactive
CD Female Clear 200Lux 1 Light Red 25lux Active
CD Female Clear 200Lux 1 Light Red 25lux Inactive
CD Female Clear 200Lux 2 Dark Clear 200lux Active
CD Female Clear 200Lux 2 Dark Clear 200lux Inactive
CD Female Clear 200Lux 2 Dark Clear 25lux Active
CD Female Clear 200Lux 2 Dark Clear 25lux Inactive
CD Female Clear 200Lux 2 Dark Red 200lux Active
CD Female Clear 200Lux 2 Dark Red 200lux Inactive
CD Female Clear 200Lux 2 Dark Red 25lux Active
CD Female Clear 200Lux 2 Dark Red 25lux Inactive
CD Female Clear 200Lux 2 Light Clear 200lux Active
CD Female Clear 200Lux 2 Light Clear 200lux Inactive
CD Female Clear 200Lux 2 Light Clear 25lux Active
CD Female Clear 200Lux 2 Light Clear 25lux Inactive

CD Female Clear 200Lux 2 Light Red 200lux Active
CD Female Clear 200Lux 2 Light Red 200lux Inactive
CD Female Clear 200Lux 2 Light Red 25lux Active
CD Female Clear 200Lux 2 Light Red 25lux Inactive
15 CD Female Clear 200Lux 3 Dark Clear 200lux Active
15 CD Female Clear 200Lux 3 Dark Clear 200lux Inactive
15 CD Female Clear 200Lux 3 Dark Clear 25lux Active
15 CD Female Clear 200Lux 3 Dark Clear 25lux Inactive
15 CD Female Clear 200Lux 3 Dark Red 200lux Active
15 CD Female Clear 200Lux 3 Dark Red 200lux Inactive
15 CD Female Clear 200Lux 3 Dark Red 25lux Active
15 CD Female Clear 200Lux 3 Dark Red 25lux Inactive
15 CD Female Clear 200Lux 3 Light Clear 200lux Active
15 CD Female Clear 200Lux 3 Light Clear 200lux Inactive
15 CD Female Clear 200Lux 3 Light Clear 25lux Active
15 CD Female Clear 200Lux 3 Light Clear 25lux Inactive
15 CD Female Clear 200Lux 3 Light Red 200lux Active
15 CD Female Clear 200Lux 3 Light Red 200lux Inactive
15 CD Female Clear 200Lux 3 Light Red 25lux Active
15 CD Female Clear 200Lux 3 Light Red 25lux Inactive
CD Female Clear 200Lux 1 Dark Clear 200lux Active
CD Female Clear 200Lux 1 Dark Clear 200lux Inactive
CD Female Clear 200Lux 1 Dark Clear 25lux Active
CD Female Clear 200Lux 1 Dark Clear 25lux Inactive
CD Female Clear 200Lux 1 Dark Red 200lux Active
CD Female Clear 200Lux 1 Dark Red 200lux Inactive
CD Female Clear 200Lux 1 Dark Red 25lux Active
CD Female Clear 200Lux 1 Dark Red 25lux Inactive
CD Female Clear 200Lux 1 Light Clear 200lux Active
CD Female Clear 200Lux 1 Light Clear 200lux Inactive
CD Female Clear 200Lux 1 Light Clear 25lux Active
CD Female Clear 200Lux 1 Light Clear 25lux Inactive
CD Female Clear 200Lux 1 Light Red 200lux Active
CD Female Clear 200Lux 1 Light Red 200lux Inactive
CD Female Clear 200Lux 1 Light Red 25lux Active
CD Female Clear 200Lux 1 Light Red 25lux Inactive
CD Female Clear 200Lux 2 Dark Clear 200lux Active
CD Female Clear 200Lux 2 Dark Clear 200lux Inactive
CD Female Clear 200Lux 2 Dark Clear 25lux Active
CD Female Clear 200Lux 2 Dark Clear 25lux Inactive
CD Female Clear 200Lux 2 Dark Red 200lux Active
CD Female Clear 200Lux 2 Dark Red 200lux Inactive
CD Female Clear 200Lux 2 Dark Red 25lux Active
CD Female Clear 200Lux 2 Dark Red 25lux Inactive
CD Female Clear 200Lux 2 Light Clear 200lux Active
CD Female Clear 200Lux 2 Light Clear 200lux Inactive
CD Female Clear 200Lux 2 Light Clear 25lux Active
CD Female Clear 200Lux 2 Light Clear 25lux Inactive
CD Female Clear 200Lux 2 Light Red 200lux Active
CD Female Clear 200Lux 2 Light Red 200lux Inactive
CD Female Clear 200Lux 2 Light Red 25lux Active
CD Female Clear 200Lux 2 Light Red 25lux Inactive
16 CD Female Clear 200Lux 3 Dark Clear 200lux Active
16 CD Female Clear 200Lux 3 Dark Clear 200lux Inactive
16 CD Female Clear 200Lux 3 Dark Clear 25lux Active

16 CD Female Clear 200Lux 3 Dark Clear 25lux Inactive
16 CD Female Clear 200Lux 3 Dark Red 200lux Active
16 CD Female Clear 200Lux 3 Dark Red 200lux Inactive
16 CD Female Clear 200Lux 3 Dark Red 25lux Active
16 CD Female Clear 200Lux 3 Dark Red 25lux Inactive
16 CD Female Clear 200Lux 3 Light Clear 200lux Active
16 CD Female Clear 200Lux 3 Light Clear 200lux Inactive
16 CD Female Clear 200Lux 3 Light Clear 25lux Active
16 CD Female Clear 200Lux 3 Light Clear 25lux Inactive
16 CD Female Clear 200Lux 3 Light Red 200lux Active
16 CD Female Clear 200Lux 3 Light Red 200lux Inactive
16 CD Female Clear 200Lux 3 Light Red 25lux Active
16 CD Female Clear 200Lux 3 Light Red 25lux Inactive
CD Male Red 200Lux 1 Dark Clear 200lux Active
CD Male Red 200Lux 1 Dark Clear 200lux Inactive
CD Male Red 200Lux 1 Dark Clear 25lux Active
CD Male Red 200Lux 1 Dark Clear 25lux Inactive
CD Male Red 200Lux 1 Dark Red 200lux Active
CD Male Red 200Lux 1 Dark Red 200lux Inactive
CD Male Red 200Lux 1 Dark Red 25lux Active
CD Male Red 200Lux 1 Dark Red 25lux Inactive
CD Male Red 200Lux 1 Light Clear 200lux Active
CD Male Red 200Lux 1 Light Clear 200lux Inactive
CD Male Red 200Lux 1 Light Clear 25lux Active
CD Male Red 200Lux 1 Light Clear 25lux Inactive
CD Male Red 200Lux 1 Light Red 200lux Active
CD Male Red 200Lux 1 Light Red 200lux Inactive
CD Male Red 200Lux 1 Light Red 25lux Active
CD Male Red 200Lux 1 Light Red 25lux Inactive
CD Male Red 200Lux 2 Dark Clear 200lux Active
CD Male Red 200Lux 2 Dark Clear 200lux Inactive
CD Male Red 200Lux 2 Dark Clear 25lux Active
CD Male Red 200Lux 2 Dark Clear 25lux Inactive
CD Male Red 200Lux 2 Dark Red 200lux Active
CD Male Red 200Lux 2 Dark Red 200lux Inactive
CD Male Red 200Lux 2 Dark Red 25lux Active
CD Male Red 200Lux 2 Dark Red 25lux Inactive
CD Male Red 200Lux 2 Light Clear 200lux Active
CD Male Red 200Lux 2 Light Clear 200lux Inactive
CD Male Red 200Lux 2 Light Clear 25lux Active
CD Male Red 200Lux 2 Light Clear 25lux Inactive
CD Male Red 200Lux 2 Light Red 200lux Active
CD Male Red 200Lux 2 Light Red 200lux Inactive
CD Male Red 200Lux 2 Light Red 25lux Active
CD Male Red 200Lux 2 Light Red 25lux Inactive
17 CD Male Red 200Lux 3 Dark Clear 200lux Active
17 CD Male Red 200Lux 3 Dark Clear 200lux Inactive
17 CD Male Red 200Lux 3 Dark Clear 25lux Active
17 CD Male Red 200Lux 3 Dark Clear 25lux Inactive
17 CD Male Red 200Lux 3 Dark Red 200lux Active
17 CD Male Red 200Lux 3 Dark Red 200lux Inactive
17 CD Male Red 200Lux 3 Dark Red 25lux Active
17 CD Male Red 200Lux 3 Dark Red 25lux Inactive
17 CD Male Red 200Lux 3 Light Clear 200lux Active
17 CD Male Red 200Lux 3 Light Clear 200lux Inactive

17 CD Male Red 200Lux 3 Light Clear 25lux Active
17 CD Male Red 200Lux 3 Light Clear 25lux Inactive
17 CD Male Red 200Lux 3 Light Red 200lux Active
17 CD Male Red 200Lux 3 Light Red 200lux Inactive
17 CD Male Red 200Lux 3 Light Red 25lux Active
17 CD Male Red 200Lux 3 Light Red 25lux Inactive
CD Male Red 200Lux 1 Dark Clear 200lux Active
CD Male Red 200Lux 1 Dark Clear 200lux Inactive
CD Male Red 200Lux 1 Dark Clear 25lux Active
CD Male Red 200Lux 1 Dark Clear 25lux Inactive
CD Male Red 200Lux 1 Dark Red 200lux Active
CD Male Red 200Lux 1 Dark Red 200lux Inactive
CD Male Red 200Lux 1 Dark Red 25lux Active
CD Male Red 200Lux 1 Dark Red 25lux Inactive
CD Male Red 200Lux 1 Light Clear 200lux Active
CD Male Red 200Lux 1 Light Clear 200lux Inactive
CD Male Red 200Lux 1 Light Clear 25lux Active
CD Male Red 200Lux 1 Light Clear 25lux Inactive
CD Male Red 200Lux 1 Light Red 200lux Active
CD Male Red 200Lux 1 Light Red 200lux Inactive
CD Male Red 200Lux 1 Light Red 25lux Active
CD Male Red 200Lux 1 Light Red 25lux Inactive
CD Male Red 200Lux 2 Dark Clear 200lux Active
CD Male Red 200Lux 2 Dark Clear 200lux Inactive
CD Male Red 200Lux 2 Dark Clear 25lux Active
CD Male Red 200Lux 2 Dark Clear 25lux Inactive
CD Male Red 200Lux 2 Dark Red 200lux Active
CD Male Red 200Lux 2 Dark Red 200lux Inactive
CD Male Red 200Lux 2 Dark Red 25lux Active
CD Male Red 200Lux 2 Dark Red 25lux Inactive
CD Male Red 200Lux 2 Light Clear 200lux Active
CD Male Red 200Lux 2 Light Clear 200lux Inactive
CD Male Red 200Lux 2 Light Clear 25lux Active
CD Male Red 200Lux 2 Light Clear 25lux Inactive
CD Male Red 200Lux 2 Light Red 200lux Active
CD Male Red 200Lux 2 Light Red 200lux Inactive
CD Male Red 200Lux 2 Light Red 25lux Active
CD Male Red 200Lux 2 Light Red 25lux Inactive
18 CD Male Red 200Lux 3 Dark Clear 200lux Active
18 CD Male Red 200Lux 3 Dark Clear 200lux Inactive
18 CD Male Red 200Lux 3 Dark Clear 25lux Active
18 CD Male Red 200Lux 3 Dark Clear 25lux Inactive
18 CD Male Red 200Lux 3 Dark Red 200lux Active
18 CD Male Red 200Lux 3 Dark Red 200lux Inactive
18 CD Male Red 200Lux 3 Dark Red 25lux Active
18 CD Male Red 200Lux 3 Dark Red 25lux Inactive
18 CD Male Red 200Lux 3 Light Clear 200lux Active
18 CD Male Red 200Lux 3 Light Clear 200lux Inactive
18 CD Male Red 200Lux 3 Light Clear 25lux Active
18 CD Male Red 200Lux 3 Light Clear 25lux Inactive
18 CD Male Red 200Lux 3 Light Red 200lux Active
18 CD Male Red 200Lux 3 Light Red 200lux Inactive
18 CD Male Red 200Lux 3 Light Red 25lux Active
18 CD Male Red 200Lux 3 Light Red 25lux Inactive
CD Female Red 200Lux 1 Dark Clear 200lux Active

CD Female Red 200Lux 1 Dark Clear 200lux Inactive
CD Female Red 200Lux 1 Dark Clear 25lux Active
CD Female Red 200Lux 1 Dark Clear 25lux Inactive
CD Female Red 200Lux 1 Dark Red 200lux Active
CD Female Red 200Lux 1 Dark Red 200lux Inactive
CD Female Red 200Lux 1 Dark Red 25lux Active
CD Female Red 200Lux 1 Dark Red 25lux Inactive
CD Female Red 200Lux 1 Light Clear 200lux Active
CD Female Red 200Lux 1 Light Clear 200lux Inactive
CD Female Red 200Lux 1 Light Clear 25lux Active
CD Female Red 200Lux 1 Light Clear 25lux Inactive
CD Female Red 200Lux 1 Light Red 200lux Active
CD Female Red 200Lux 1 Light Red 200lux Inactive
CD Female Red 200Lux 1 Light Red 25lux Active
CD Female Red 200Lux 1 Light Red 25lux Inactive
CD Female Red 200Lux 2 Dark Clear 200lux Active
CD Female Red 200Lux 2 Dark Clear 200lux Inactive
CD Female Red 200Lux 2 Dark Clear 25lux Active
CD Female Red 200Lux 2 Dark Clear 25lux Inactive
CD Female Red 200Lux 2 Dark Red 200lux Active
CD Female Red 200Lux 2 Dark Red 200lux Inactive
CD Female Red 200Lux 2 Dark Red 25lux Active
CD Female Red 200Lux 2 Dark Red 25lux Inactive
CD Female Red 200Lux 2 Light Clear 200lux Active
CD Female Red 200Lux 2 Light Clear 200lux Inactive
CD Female Red 200Lux 2 Light Clear 25lux Active
CD Female Red 200Lux 2 Light Clear 25lux Inactive
CD Female Red 200Lux 2 Light Red 200lux Active
CD Female Red 200Lux 2 Light Red 200lux Inactive
CD Female Red 200Lux 2 Light Red 25lux Active
CD Female Red 200Lux 2 Light Red 25lux Inactive
19 CD Female Red 200Lux 3 Dark Clear 200lux Active
19 CD Female Red 200Lux 3 Dark Clear 200lux Inactive
19 CD Female Red 200Lux 3 Dark Clear 25lux Active
19 CD Female Red 200Lux 3 Dark Clear 25lux Inactive
19 CD Female Red 200Lux 3 Dark Red 200lux Active
19 CD Female Red 200Lux 3 Dark Red 200lux Inactive
19 CD Female Red 200Lux 3 Dark Red 25lux Active
19 CD Female Red 200Lux 3 Dark Red 25lux Inactive
19 CD Female Red 200Lux 3 Light Clear 200lux Active
19 CD Female Red 200Lux 3 Light Clear 200lux Inactive
19 CD Female Red 200Lux 3 Light Clear 25lux Active
19 CD Female Red 200Lux 3 Light Clear 25lux Inactive
19 CD Female Red 200Lux 3 Light Red 200lux Active
19 CD Female Red 200Lux 3 Light Red 200lux Inactive
19 CD Female Red 200Lux 3 Light Red 25lux Active
19 CD Female Red 200Lux 3 Light Red 25lux Inactive
CD Female Red 200Lux 1 Dark Clear 200lux Active
CD Female Red 200Lux 1 Dark Clear 200lux Inactive
CD Female Red 200Lux 1 Dark Clear 25lux Active
CD Female Red 200Lux 1 Dark Clear 25lux Inactive
CD Female Red 200Lux 1 Dark Red 200lux Active
CD Female Red 200Lux 1 Dark Red 200lux Inactive
CD Female Red 200Lux 1 Dark Red 25lux Active
CD Female Red 200Lux 1 Dark Red 25lux Inactive

CD Female Red 200Lux 1 Light Clear 200lux Active
CD Female Red 200Lux 1 Light Clear 200lux Inactive
CD Female Red 200Lux 1 Light Clear 25lux Active
CD Female Red 200Lux 1 Light Clear 25lux Inactive
CD Female Red 200Lux 1 Light Red 200lux Active
CD Female Red 200Lux 1 Light Red 200lux Inactive
CD Female Red 200Lux 1 Light Red 25lux Active
CD Female Red 200Lux 1 Light Red 25lux Inactive
CD Female Red 200Lux 2 Dark Clear 200lux Active
CD Female Red 200Lux 2 Dark Clear 200lux Inactive
CD Female Red 200Lux 2 Dark Clear 25lux Active
CD Female Red 200Lux 2 Dark Clear 25lux Inactive
CD Female Red 200Lux 2 Dark Red 200lux Active
CD Female Red 200Lux 2 Dark Red 200lux Inactive
CD Female Red 200Lux 2 Dark Red 25lux Active
CD Female Red 200Lux 2 Dark Red 25lux Inactive
CD Female Red 200Lux 2 Light Clear 200lux Active
CD Female Red 200Lux 2 Light Clear 200lux Inactive
CD Female Red 200Lux 2 Light Clear 25lux Active
CD Female Red 200Lux 2 Light Clear 25lux Inactive
CD Female Red 200Lux 2 Light Red 200lux Active
CD Female Red 200Lux 2 Light Red 200lux Inactive
CD Female Red 200Lux 2 Light Red 25lux Active
CD Female Red 200Lux 2 Light Red 25lux Inactive
20 CD Female Red 200Lux 3 Dark Clear 200lux Active
20 CD Female Red 200Lux 3 Dark Clear 200lux Inactive
20 CD Female Red 200Lux 3 Dark Clear 25lux Active
20 CD Female Red 200Lux 3 Dark Clear 25lux Inactive
20 CD Female Red 200Lux 3 Dark Red 200lux Active
20 CD Female Red 200Lux 3 Dark Red 200lux Inactive
20 CD Female Red 200Lux 3 Dark Red 25lux Active
20 CD Female Red 200Lux 3 Dark Red 25lux Inactive
20 CD Female Red 200Lux 3 Light Clear 200lux Active
20 CD Female Red 200Lux 3 Light Clear 200lux Inactive
20 CD Female Red 200Lux 3 Light Clear 25lux Active
20 CD Female Red 200Lux 3 Light Clear 25lux Inactive
20 CD Female Red 200Lux 3 Light Red 200lux Active
20 CD Female Red 200Lux 3 Light Red 200lux Inactive
20 CD Female Red 200Lux 3 Light Red 25lux Active
20 CD Female Red 200Lux 3 Light Red 25lux Inactive
CD Male Clear 25Lux 1 Dark Clear 200lux Active
CD Male Clear 25Lux 1 Dark Clear 200lux Inactive
CD Male Clear 25Lux 1 Dark Clear 25lux Active
CD Male Clear 25Lux 1 Dark Clear 25lux Inactive
CD Male Clear 25Lux 1 Dark Red 200lux Active
CD Male Clear 25Lux 1 Dark Red 200lux Inactive
CD Male Clear 25Lux 1 Dark Red 25lux Active
CD Male Clear 25Lux 1 Dark Red 25lux Inactive
CD Male Clear 25Lux 1 Light Clear 200lux Active
CD Male Clear 25Lux 1 Light Clear 200lux Inactive
CD Male Clear 25Lux 1 Light Clear 25lux Active
CD Male Clear 25Lux 1 Light Clear 25lux Inactive
CD Male Clear 25Lux 1 Light Red 200lux Active
CD Male Clear 25Lux 1 Light Red 200lux Inactive
CD Male Clear 25Lux 1 Light Red 25lux Active

CD Male Clear 25Lux 1 Light Red 25lux Inactive
CD Male Clear 25Lux 2 Dark Clear 200lux Active
CD Male Clear 25Lux 2 Dark Clear 200lux Inactive
CD Male Clear 25Lux 2 Dark Clear 25lux Active
CD Male Clear 25Lux 2 Dark Clear 25lux Inactive
CD Male Clear 25Lux 2 Dark Red 200lux Active
CD Male Clear 25Lux 2 Dark Red 200lux Inactive
CD Male Clear 25Lux 2 Dark Red 25lux Active
CD Male Clear 25Lux 2 Dark Red 25lux Inactive
CD Male Clear 25Lux 2 Light Clear 200lux Active
CD Male Clear 25Lux 2 Light Clear 200lux Inactive
CD Male Clear 25Lux 2 Light Clear 25lux Active
CD Male Clear 25Lux 2 Light Clear 25lux Inactive
CD Male Clear 25Lux 2 Light Red 200lux Active
CD Male Clear 25Lux 2 Light Red 200lux Inactive
CD Male Clear 25Lux 2 Light Red 25lux Active
CD Male Clear 25Lux 2 Light Red 25lux Inactive
21 CD Male Clear 25Lux 3 Dark Clear 200lux Active
21 CD Male Clear 25Lux 3 Dark Clear 200lux Inactive
21 CD Male Clear 25Lux 3 Dark Clear 25lux Active
21 CD Male Clear 25Lux 3 Dark Clear 25lux Inactive
21 CD Male Clear 25Lux 3 Dark Red 200lux Active
21 CD Male Clear 25Lux 3 Dark Red 200lux Inactive
21 CD Male Clear 25Lux 3 Dark Red 25lux Active
21 CD Male Clear 25Lux 3 Dark Red 25lux Inactive
21 CD Male Clear 25Lux 3 Light Clear 200lux Active
21 CD Male Clear 25Lux 3 Light Clear 200lux Inactive
21 CD Male Clear 25Lux 3 Light Clear 25lux Active
21 CD Male Clear 25Lux 3 Light Clear 25lux Inactive
21 CD Male Clear 25Lux 3 Light Red 200lux Active
21 CD Male Clear 25Lux 3 Light Red 200lux Inactive
21 CD Male Clear 25Lux 3 Light Red 25lux Active
21 CD Male Clear 25Lux 3 Light Red 25lux Inactive
CD Female Clear 25Lux 1 Dark Clear 200lux Active
CD Female Clear 25Lux 1 Dark Clear 200lux Inactive
CD Female Clear 25Lux 1 Dark Clear 25lux Active
CD Female Clear 25Lux 1 Dark Clear 25lux Inactive
CD Female Clear 25Lux 1 Dark Red 200lux Active
CD Female Clear 25Lux 1 Dark Red 200lux Inactive
CD Female Clear 25Lux 1 Dark Red 25lux Active
CD Female Clear 25Lux 1 Dark Red 25lux Inactive
CD Female Clear 25Lux 1 Light Clear 200lux Active
CD Female Clear 25Lux 1 Light Clear 200lux Inactive
CD Female Clear 25Lux 1 Light Clear 25lux Active
CD Female Clear 25Lux 1 Light Clear 25lux Inactive
CD Female Clear 25Lux 1 Light Red 200lux Active
CD Female Clear 25Lux 1 Light Red 200lux Inactive
CD Female Clear 25Lux 1 Light Red 25lux Active
CD Female Clear 25Lux 1 Light Red 25lux Inactive
CD Female Clear 25Lux 2 Dark Clear 200lux Active
CD Female Clear 25Lux 2 Dark Clear 200lux Inactive
CD Female Clear 25Lux 2 Dark Clear 25lux Active
CD Female Clear 25Lux 2 Dark Clear 25lux Inactive
CD Female Clear 25Lux 2 Dark Red 200lux Active
CD Female Clear 25Lux 2 Dark Red 200lux Inactive

CD Female Clear 25Lux 2 Dark Red 25lux Active
CD Female Clear 25Lux 2 Dark Red 25lux Inactive
CD Female Clear 25Lux 2 Light Clear 200lux Active
CD Female Clear 25Lux 2 Light Clear 200lux Inactive
CD Female Clear 25Lux 2 Light Clear 25lux Active
CD Female Clear 25Lux 2 Light Clear 25lux Inactive
CD Female Clear 25Lux 2 Light Red 200lux Active
CD Female Clear 25Lux 2 Light Red 200lux Inactive
CD Female Clear 25Lux 2 Light Red 25lux Active
CD Female Clear 25Lux 2 Light Red 25lux Inactive
22 CD Female Clear 25Lux 3 Dark Clear 200lux Active
22 CD Female Clear 25Lux 3 Dark Clear 200lux Inactive
22 CD Female Clear 25Lux 3 Dark Clear 25lux Active
22 CD Female Clear 25Lux 3 Dark Clear 25lux Inactive
22 CD Female Clear 25Lux 3 Dark Red 200lux Active
22 CD Female Clear 25Lux 3 Dark Red 200lux Inactive
22 CD Female Clear 25Lux 3 Dark Red 25lux Active
22 CD Female Clear 25Lux 3 Dark Red 25lux Inactive
22 CD Female Clear 25Lux 3 Light Clear 200lux Active
22 CD Female Clear 25Lux 3 Light Clear 200lux Inactive
22 CD Female Clear 25Lux 3 Light Clear 25lux Active
22 CD Female Clear 25Lux 3 Light Clear 25lux Inactive
22 CD Female Clear 25Lux 3 Light Red 200lux Active
22 CD Female Clear 25Lux 3 Light Red 200lux Inactive
22 CD Female Clear 25Lux 3 Light Red 25lux Active
22 CD Female Clear 25Lux 3 Light Red 25lux Inactive
CD Male Red 25Lux 1 Dark Clear 200lux Active
CD Male Red 25Lux 1 Dark Clear 200lux Inactive
CD Male Red 25Lux 1 Dark Clear 25lux Active
CD Male Red 25Lux 1 Dark Clear 25lux Inactive
CD Male Red 25Lux 1 Dark Red 200lux Active
CD Male Red 25Lux 1 Dark Red 200lux Inactive
CD Male Red 25Lux 1 Dark Red 25lux Active
CD Male Red 25Lux 1 Dark Red 25lux Inactive
CD Male Red 25Lux 1 Light Clear 200lux Active
CD Male Red 25Lux 1 Light Clear 200lux Inactive
CD Male Red 25Lux 1 Light Clear 25lux Active
CD Male Red 25Lux 1 Light Clear 25lux Inactive
CD Male Red 25Lux 1 Light Red 200lux Active
CD Male Red 25Lux 1 Light Red 200lux Inactive
CD Male Red 25Lux 1 Light Red 25lux Active
CD Male Red 25Lux 1 Light Red 25lux Inactive
CD Male Red 25Lux 2 Dark Clear 200lux Active
CD Male Red 25Lux 2 Dark Clear 200lux Inactive
CD Male Red 25Lux 2 Dark Clear 25lux Active
CD Male Red 25Lux 2 Dark Clear 25lux Inactive
CD Male Red 25Lux 2 Dark Red 200lux Active
CD Male Red 25Lux 2 Dark Red 200lux Inactive
CD Male Red 25Lux 2 Dark Red 25lux Active
CD Male Red 25Lux 2 Dark Red 25lux Inactive
CD Male Red 25Lux 2 Light Clear 200lux Active
CD Male Red 25Lux 2 Light Clear 200lux Inactive
CD Male Red 25Lux 2 Light Clear 25lux Active
CD Male Red 25Lux 2 Light Clear 25lux Inactive
CD Male Red 25Lux 2 Light Red 200lux Active

CD Male Red 25Lux 2 Light Red 200lux Inactive
CD Male Red 25Lux 2 Light Red 25lux Active
CD Male Red 25Lux 2 Light Red 25lux Inactive
23 CD Male Red 25Lux 3 Dark Clear 200lux Active
23 CD Male Red 25Lux 3 Dark Clear 200lux Inactive
23 CD Male Red 25Lux 3 Dark Clear 25lux Active
23 CD Male Red 25Lux 3 Dark Clear 25lux Inactive
23 CD Male Red 25Lux 3 Dark Red 200lux Active
23 CD Male Red 25Lux 3 Dark Red 200lux Inactive
23 CD Male Red 25Lux 3 Dark Red 25lux Active
23 CD Male Red 25Lux 3 Dark Red 25lux Inactive
23 CD Male Red 25Lux 3 Light Clear 200lux Active
23 CD Male Red 25Lux 3 Light Clear 200lux Inactive
23 CD Male Red 25Lux 3 Light Clear 25lux Active
23 CD Male Red 25Lux 3 Light Clear 25lux Inactive
23 CD Male Red 25Lux 3 Light Red 200lux Active
23 CD Male Red 25Lux 3 Light Red 200lux Inactive
23 CD Male Red 25Lux 3 Light Red 25lux Active
23 CD Male Red 25Lux 3 Light Red 25lux Inactive
CD Female Red 25Lux 1 Dark Clear 200lux Active
CD Female Red 25Lux 1 Dark Clear 200lux Inactive
CD Female Red 25Lux 1 Dark Clear 25lux Active
CD Female Red 25Lux 1 Dark Clear 25lux Inactive
CD Female Red 25Lux 1 Dark Red 200lux Active
CD Female Red 25Lux 1 Dark Red 200lux Inactive
CD Female Red 25Lux 1 Dark Red 25lux Active
CD Female Red 25Lux 1 Dark Red 25lux Inactive
CD Female Red 25Lux 1 Light Clear 200lux Active
CD Female Red 25Lux 1 Light Clear 200lux Inactive
CD Female Red 25Lux 1 Light Clear 25lux Active
CD Female Red 25Lux 1 Light Clear 25lux Inactive
CD Female Red 25Lux 1 Light Red 200lux Active
CD Female Red 25Lux 1 Light Red 200lux Inactive
CD Female Red 25Lux 1 Light Red 25lux Active
CD Female Red 25Lux 1 Light Red 25lux Inactive
CD Female Red 25Lux 2 Dark Clear 200lux Active
CD Female Red 25Lux 2 Dark Clear 200lux Inactive
CD Female Red 25Lux 2 Dark Clear 25lux Active
CD Female Red 25Lux 2 Dark Clear 25lux Inactive
CD Female Red 25Lux 2 Dark Red 200lux Active
CD Female Red 25Lux 2 Dark Red 200lux Inactive
CD Female Red 25Lux 2 Dark Red 25lux Active
CD Female Red 25Lux 2 Dark Red 25lux Inactive
CD Female Red 25Lux 2 Light Clear 200lux Active
CD Female Red 25Lux 2 Light Clear 200lux Inactive
CD Female Red 25Lux 2 Light Clear 25lux Active
CD Female Red 25Lux 2 Light Clear 25lux Inactive
CD Female Red 25Lux 2 Light Red 200lux Active
CD Female Red 25Lux 2 Light Red 200lux Inactive
CD Female Red 25Lux 2 Light Red 25lux Active
CD Female Red 25Lux 2 Light Red 25lux Inactive
24 CD Female Red 25Lux 3 Dark Clear 200lux Active
24 CD Female Red 25Lux 3 Dark Clear 200lux Inactive
24 CD Female Red 25Lux 3 Dark Clear 25lux Active
24 CD Female Red 25Lux 3 Dark Clear 25lux Inactive

24 CD Female Red 25Lux 3 Dark Red 200lux Active
24 CD Female Red 25Lux 3 Dark Red 200lux Inactive
24 CD Female Red 25Lux 3 Dark Red 25lux Active
24 CD Female Red 25Lux 3 Dark Red 25lux Inactive
24 CD Female Red 25Lux 3 Light Clear 200lux Active
24 CD Female Red 25Lux 3 Light Clear 200lux Inactive
24 CD Female Red 25Lux 3 Light Clear 25lux Active
24 CD Female Red 25Lux 3 Light Clear 25lux Inactive
24 CD Female Red 25Lux 3 Light Red 200lux Active
24 CD Female Red 25Lux 3 Light Red 200lux Inactive
24 CD Female Red 25Lux 3 Light Red 25lux Active
24 CD Female Red 25Lux 3 Light Red 25lux Inactive
LE Male Clear 25Lux 1 Dark Clear 200lux Active
LE Male Clear 25Lux 1 Dark Red 25lux Inactive
LE Male Clear 25Lux 1 Dark Red 25lux Active
LE Male Clear 25Lux 1 Dark Red 200lux Inactive
LE Male Clear 25Lux 1 Dark Red 200lux Active
LE Male Clear 25Lux 1 Dark Clear 200lux Inactive
LE Male Clear 25Lux 1 Dark Clear 25lux Inactive
LE Male Clear 25Lux 1 Dark Clear 25lux Active
LE Male Clear 25Lux 1 Light Red 25lux Inactive
LE Male Clear 25Lux 1 Light Clear 25lux Active
LE Male Clear 25Lux 1 Light Red 200lux Inactive
LE Male Clear 25Lux 1 Light Clear 25lux Inactive
LE Male Clear 25Lux 1 Light Red 200lux Active
LE Male Clear 25Lux 1 Light Red 25lux Active
LE Male Clear 25Lux 1 Light Clear 200lux Inactive
LE Male Clear 25Lux 1 Light Clear 200lux Active
LE Male Clear 25Lux 2 Dark Red 25lux Active
LE Male Clear 25Lux 2 Dark Red 25lux Inactive
LE Male Clear 25Lux 2 Dark Red 200lux Inactive
LE Male Clear 25Lux 2 Dark Red 200lux Active
LE Male Clear 25Lux 2 Dark Clear 25lux Inactive
LE Male Clear 25Lux 2 Dark Clear 25lux Active
LE Male Clear 25Lux 2 Dark Clear 200lux Active
LE Male Clear 25Lux 2 Dark Clear 200lux Inactive
LE Male Clear 25Lux 2 Light Red 25lux Inactive
LE Male Clear 25Lux 2 Light Clear 25lux Active
LE Male Clear 25Lux 2 Light Clear 200lux Inactive
LE Male Clear 25Lux 2 Light Red 200lux Inactive
LE Male Clear 25Lux 2 Light Red 200lux Active
LE Male Clear 25Lux 2 Light Clear 25lux Inactive
LE Male Clear 25Lux 2 Light Red 25lux Active
LE Male Clear 25Lux 2 Light Clear 200lux Active
LE Male Clear 25Lux 3 Dark Red 200lux Active
LE Male Clear 25Lux 3 Dark Red 200lux Inactive
LE Male Clear 25Lux 3 Dark Clear 25lux Active
LE Male Clear 25Lux 3 Dark Red 25lux Active
LE Male Clear 25Lux 3 Dark Red 25lux Inactive
LE Male Clear 25Lux 3 Dark Clear 25lux Inactive
LE Male Clear 25Lux 3 Dark Clear 200lux Inactive
LE Male Clear 25Lux 3 Dark Clear 200lux Active
LE Male Clear 25Lux 3 Light Red 25lux Inactive
LE Male Clear 25Lux 3 Light Clear 200lux Active
LE Male Clear 25Lux 3 Light Clear 200lux Inactive

LE	Male	Clear	25Lux	3	Light	Red	200lux	Inactive
LE	Male	Clear	25Lux	3	Light	Red	200lux	Active
LE	Male	Clear	25Lux	3	Light	Clear	25lux	Inactive
LE	Male	Clear	25Lux	3	Light	Clear	25lux	Active
LE	Male	Clear	25Lux	3	Light	Red	25lux	Active
LE	Male	Clear	25Lux	1	Dark	Red	25lux	Active
LE	Male	Clear	25Lux	1	Dark	Red	25lux	Inactive
LE	Male	Clear	25Lux	1	Dark	Red	200lux	Active
LE	Male	Clear	25Lux	1	Dark	Red	200lux	Inactive
LE	Male	Clear	25Lux	1	Dark	Clear	200lux	Inactive
LE	Male	Clear	25Lux	1	Dark	Clear	25lux	Inactive
LE	Male	Clear	25Lux	1	Dark	Clear	25lux	Active
LE	Male	Clear	25Lux	1	Dark	Clear	200lux	Active
LE	Male	Clear	25Lux	1	Light	Red	200lux	Active
LE	Male	Clear	25Lux	1	Light	Red	25lux	Inactive
LE	Male	Clear	25Lux	1	Light	Clear	25lux	Active
LE	Male	Clear	25Lux	1	Light	Clear	25lux	Inactive
LE	Male	Clear	25Lux	1	Light	Red	25lux	Active
LE	Male	Clear	25Lux	1	Light	Red	200lux	Inactive
LE	Male	Clear	25Lux	1	Light	Clear	200lux	Inactive
LE	Male	Clear	25Lux	1	Light	Clear	200lux	Active
LE	Male	Clear	25Lux	2	Dark	Red	200lux	Active
LE	Male	Clear	25Lux	2	Dark	Red	25lux	Inactive
LE	Male	Clear	25Lux	2	Dark	Red	25lux	Active
LE	Male	Clear	25Lux	2	Dark	Red	200lux	Inactive
LE	Male	Clear	25Lux	2	Dark	Clear	200lux	Active
LE	Male	Clear	25Lux	2	Dark	Clear	25lux	Inactive
LE	Male	Clear	25Lux	2	Dark	Clear	25lux	Active
LE	Male	Clear	25Lux	2	Dark	Clear	200lux	Inactive
LE	Male	Clear	25Lux	2	Light	Red	25lux	Inactive
LE	Male	Clear	25Lux	2	Light	Red	25lux	Active
LE	Male	Clear	25Lux	2	Light	Clear	25lux	Inactive
LE	Male	Clear	25Lux	2	Light	Clear	200lux	Active
LE	Male	Clear	25Lux	2	Light	Red	200lux	Active
LE	Male	Clear	25Lux	2	Light	Red	200lux	Inactive
LE	Male	Clear	25Lux	2	Light	Clear	200lux	Inactive
LE	Male	Clear	25Lux	2	Light	Clear	25lux	Active
LE	Male	Clear	25Lux	3	Dark	Red	200lux	Active
LE	Male	Clear	25Lux	3	Dark	Red	200lux	Inactive
LE	Male	Clear	25Lux	3	Dark	Red	25lux	Inactive
LE	Male	Clear	25Lux	3	Dark	Red	25lux	Active
LE	Male	Clear	25Lux	3	Dark	Clear	200lux	Active
LE	Male	Clear	25Lux	3	Dark	Clear	25lux	Inactive
LE	Male	Clear	25Lux	3	Dark	Clear	200lux	Inactive
LE	Male	Clear	25Lux	3	Dark	Clear	25lux	Active
LE	Male	Clear	25Lux	3	Light	Red	25lux	Inactive
LE	Male	Clear	25Lux	3	Light	Red	200lux	Active
LE	Male	Clear	25Lux	3	Light	Clear	25lux	Active
LE	Male	Clear	25Lux	3	Light	Clear	200lux	Inactive
LE	Male	Clear	25Lux	3	Light	Red	200lux	Inactive
LE	Male	Clear	25Lux	3	Light	Red	25lux	Active
LE	Male	Clear	25Lux	3	Light	Clear	25lux	Inactive
LE	Male	Clear	25Lux	3	Light	Clear	200lux	Active
LE	Female	Clear	25Lux	1	Dark	Red	200lux	Active
LE	Female	Clear	25Lux	1	Dark	Red	200lux	Inactive

LE	Female	Clear	25Lux	1	Dark	Red	25lux	Inactive
LE	Female	Clear	25Lux	1	Dark	Red	25lux	Active
LE	Female	Clear	25Lux	1	Dark	Clear	25lux	Inactive
LE	Female	Clear	25Lux	1	Dark	Clear	25lux	Active
LE	Female	Clear	25Lux	1	Dark	Clear	200lux	Active
LE	Female	Clear	25Lux	1	Dark	Clear	200lux	Inactive
LE	Female	Clear	25Lux	1	Light	Red	25lux	Inactive
LE	Female	Clear	25Lux	1	Light	Red	25lux	Active
LE	Female	Clear	25Lux	1	Light	Clear	200lux	Inactive
LE	Female	Clear	25Lux	1	Light	Clear	200lux	Active
LE	Female	Clear	25Lux	1	Light	Clear	25lux	Active
LE	Female	Clear	25Lux	1	Light	Clear	25lux	Inactive
LE	Female	Clear	25Lux	1	Light	Red	200lux	Active
LE	Female	Clear	25Lux	1	Light	Red	200lux	Inactive
LE	Female	Clear	25Lux	2	Dark	Clear	25lux	Active
LE	Female	Clear	25Lux	2	Dark	Red	25lux	Active
LE	Female	Clear	25Lux	2	Dark	Red	200lux	Inactive
LE	Female	Clear	25Lux	2	Dark	Red	200lux	Active
LE	Female	Clear	25Lux	2	Dark	Red	25lux	Inactive
LE	Female	Clear	25Lux	2	Dark	Clear	25lux	Inactive
LE	Female	Clear	25Lux	2	Dark	Clear	200lux	Inactive
LE	Female	Clear	25Lux	2	Dark	Clear	200lux	Active
LE	Female	Clear	25Lux	2	Light	Red	25lux	Inactive
LE	Female	Clear	25Lux	2	Light	Red	25lux	Active
LE	Female	Clear	25Lux	2	Light	Clear	200lux	Inactive
LE	Female	Clear	25Lux	2	Light	Red	200lux	Active
LE	Female	Clear	25Lux	2	Light	Clear	25lux	Inactive
LE	Female	Clear	25Lux	2	Light	Clear	25lux	Active
LE	Female	Clear	25Lux	2	Light	Red	200lux	Inactive
LE	Female	Clear	25Lux	2	Light	Clear	200lux	Active
LE	Female	Clear	25Lux	3	Dark	Red	200lux	Inactive
LE	Female	Clear	25Lux	3	Dark	Red	25lux	Active
LE	Female	Clear	25Lux	3	Dark	Red	25lux	Inactive
LE	Female	Clear	25Lux	3	Dark	Clear	25lux	Active
LE	Female	Clear	25Lux	3	Dark	Red	200lux	Active
LE	Female	Clear	25Lux	3	Dark	Clear	200lux	Inactive
LE	Female	Clear	25Lux	3	Dark	Clear	200lux	Active
LE	Female	Clear	25Lux	3	Dark	Clear	25lux	Inactive
LE	Female	Clear	25Lux	3	Light	Red	25lux	Inactive
LE	Female	Clear	25Lux	3	Light	Clear	25lux	Inactive
LE	Female	Clear	25Lux	3	Light	Red	25lux	Active
LE	Female	Clear	25Lux	3	Light	Clear	25lux	Active
LE	Female	Clear	25Lux	3	Light	Clear	200lux	Active
LE	Female	Clear	25Lux	3	Light	Clear	200lux	Inactive
LE	Female	Clear	25Lux	3	Light	Red	200lux	Inactive
LE	Female	Clear	25Lux	3	Light	Red	200lux	Active
LE	Female	Clear	25Lux	1	Dark	Red	200lux	Inactive
LE	Female	Clear	25Lux	1	Dark	Red	200lux	Active
LE	Female	Clear	25Lux	1	Dark	Red	25lux	Active
LE	Female	Clear	25Lux	1	Dark	Red	25lux	Inactive
LE	Female	Clear	25Lux	1	Dark	Clear	25lux	Inactive
LE	Female	Clear	25Lux	1	Dark	Clear	25lux	Active
LE	Female	Clear	25Lux	1	Dark	Clear	200lux	Active
LE	Female	Clear	25Lux	1	Dark	Clear	200lux	Inactive
LE	Female	Clear	25Lux	1	Light	Clear	200lux	Active

LE Female Clear 25Lux 1 Light Red 25lux Inactive
LE Female Clear 25Lux 1 Light Clear 200lux Inactive
LE Female Clear 25Lux 1 Light Red 25lux Active
LE Female Clear 25Lux 1 Light Clear 25lux Active
LE Female Clear 25Lux 1 Light Clear 25lux Inactive
LE Female Clear 25Lux 1 Light Red 200lux Active
LE Female Clear 25Lux 1 Light Red 200lux Inactive
LE Female Clear 25Lux 2 Dark Red 200lux Inactive
LE Female Clear 25Lux 2 Dark Red 200lux Active
LE Female Clear 25Lux 2 Dark Clear 25lux Inactive
LE Female Clear 25Lux 2 Dark Red 25lux Inactive
LE Female Clear 25Lux 2 Dark Red 25lux Active
LE Female Clear 25Lux 2 Dark Clear 25lux Active
LE Female Clear 25Lux 2 Dark Clear 200lux Inactive
LE Female Clear 25Lux 2 Dark Clear 200lux Active
LE Female Clear 25Lux 2 Light Red 25lux Active
LE Female Clear 25Lux 2 Light Red 25lux Inactive
LE Female Clear 25Lux 2 Light Clear 200lux Inactive
LE Female Clear 25Lux 2 Light Red 200lux Inactive
LE Female Clear 25Lux 2 Light Clear 200lux Active
LE Female Clear 25Lux 2 Light Clear 25lux Active
LE Female Clear 25Lux 2 Light Clear 25lux Inactive
LE Female Clear 25Lux 2 Light Red 200lux Active
4 LE Female Clear 25Lux 3 Dark Red 200lux Active
4 LE Female Clear 25Lux 3 Dark Red 25lux Inactive
4 LE Female Clear 25Lux 3 Dark Red 25lux Active
4 LE Female Clear 25Lux 3 Dark Red 200lux Inactive
4 LE Female Clear 25Lux 3 Dark Clear 25lux Inactive
4 LE Female Clear 25Lux 3 Dark Clear 25lux Active
4 LE Female Clear 25Lux 3 Dark Clear 200lux Active
4 LE Female Clear 25Lux 3 Dark Clear 200lux Inactive
4 LE Female Clear 25Lux 3 Light Clear 25lux Active
4 LE Female Clear 25Lux 3 Light Red 25lux Inactive
4 LE Female Clear 25Lux 3 Light Clear 25lux Inactive
4 LE Female Clear 25Lux 3 Light Clear 200lux Active
4 LE Female Clear 25Lux 3 Light Clear 200lux Inactive
4 LE Female Clear 25Lux 3 Light Red 200lux Inactive
4 LE Female Clear 25Lux 3 Light Red 200lux Active
4 LE Female Clear 25Lux 3 Light Red 25lux Active
LE Male Red 25Lux 1 Dark Red 200lux Inactive
LE Male Red 25Lux 1 Dark Red 25lux Active
LE Male Red 25Lux 1 Dark Red 25lux Inactive
LE Male Red 25Lux 1 Dark Red 200lux Active
LE Male Red 25Lux 1 Dark Clear 200lux Inactive
LE Male Red 25Lux 1 Dark Clear 25lux Inactive
LE Male Red 25Lux 1 Dark Clear 25lux Active
LE Male Red 25Lux 1 Dark Clear 200lux Active
LE Male Red 25Lux 1 Light Clear 25lux Active
LE Male Red 25Lux 1 Light Red 25lux Inactive
LE Male Red 25Lux 1 Light Red 25lux Active
LE Male Red 25Lux 1 Light Clear 200lux Inactive
LE Male Red 25Lux 1 Light Red 200lux Inactive
LE Male Red 25Lux 1 Light Red 200lux Active
LE Male Red 25Lux 1 Light Clear 200lux Active
LE Male Red 25Lux 1 Light Clear 25lux Inactive

LE Male Red 25Lux 2 Dark Red 200lux Active
LE Male Red 25Lux 2 Dark Red 200lux Inactive
LE Male Red 25Lux 2 Dark Red 25lux Active
LE Male Red 25Lux 2 Dark Red 25lux Inactive
LE Male Red 25Lux 2 Dark Clear 25lux Inactive
LE Male Red 25Lux 2 Dark Clear 25lux Active
LE Male Red 25Lux 2 Dark Clear 200lux Active
LE Male Red 25Lux 2 Dark Clear 200lux Inactive
LE Male Red 25Lux 2 Light Red 25lux Inactive
LE Male Red 25Lux 2 Light Red 25lux Active
LE Male Red 25Lux 2 Light Red 200lux Inactive
LE Male Red 25Lux 2 Light Red 200lux Active
LE Male Red 25Lux 2 Light Clear 25lux Active
LE Male Red 25Lux 2 Light Clear 200lux Inactive
LE Male Red 25Lux 2 Light Clear 200lux Active
LE Male Red 25Lux 2 Light Clear 25lux Inactive
5 LE Male Red 25Lux 3 Dark Red 200lux Active
5 LE Male Red 25Lux 3 Dark Red 200lux Inactive
5 LE Male Red 25Lux 3 Dark Red 25lux Active
5 LE Male Red 25Lux 3 Dark Red 25lux Inactive
5 LE Male Red 25Lux 3 Dark Clear 25lux Inactive
5 LE Male Red 25Lux 3 Dark Clear 25lux Active
5 LE Male Red 25Lux 3 Dark Clear 200lux Inactive
5 LE Male Red 25Lux 3 Dark Clear 200lux Active
5 LE Male Red 25Lux 3 Light Clear 25lux Active
5 LE Male Red 25Lux 3 Light Red 25lux Inactive
5 LE Male Red 25Lux 3 Light Red 25lux Active
5 LE Male Red 25Lux 3 Light Clear 200lux Inactive
5 LE Male Red 25Lux 3 Light Red 200lux Inactive
5 LE Male Red 25Lux 3 Light Red 200lux Active
5 LE Male Red 25Lux 3 Light Clear 200lux Active
5 LE Male Red 25Lux 3 Light Clear 25lux Inactive
LE Male Red 25Lux 1 Dark Red 200lux Active
LE Male Red 25Lux 1 Dark Red 200lux Inactive
LE Male Red 25Lux 1 Dark Red 25lux Inactive
LE Male Red 25Lux 1 Dark Red 25lux Active
LE Male Red 25Lux 1 Dark Clear 25lux Inactive
LE Male Red 25Lux 1 Dark Clear 25lux Active
LE Male Red 25Lux 1 Dark Clear 200lux Active
LE Male Red 25Lux 1 Dark Clear 200lux Inactive
LE Male Red 25Lux 1 Light Red 25lux Inactive
LE Male Red 25Lux 1 Light Red 25lux Active
LE Male Red 25Lux 1 Light Clear 200lux Active
LE Male Red 25Lux 1 Light Red 200lux Inactive
LE Male Red 25Lux 1 Light Clear 200lux Inactive
LE Male Red 25Lux 1 Light Clear 25lux Active
LE Male Red 25Lux 1 Light Clear 25lux Inactive
LE Male Red 25Lux 1 Light Red 200lux Active
LE Male Red 25Lux 2 Dark Red 25lux Active
LE Male Red 25Lux 2 Dark Red 200lux Inactive
LE Male Red 25Lux 2 Dark Red 200lux Active
LE Male Red 25Lux 2 Dark Red 25lux Inactive
LE Male Red 25Lux 2 Dark Clear 25lux Active
LE Male Red 25Lux 2 Dark Clear 200lux Inactive
LE Male Red 25Lux 2 Dark Clear 200lux Active

LE Male Red 25Lux 2 Dark Clear 25lux Inactive
LE Male Red 25Lux 2 Light Red 25lux Inactive
LE Male Red 25Lux 2 Light Red 25lux Active
LE Male Red 25Lux 2 Light Clear 200lux Active
LE Male Red 25Lux 2 Light Red 200lux Inactive
LE Male Red 25Lux 2 Light Red 200lux Active
LE Male Red 25Lux 2 Light Clear 25lux Active
LE Male Red 25Lux 2 Light Clear 200lux Inactive
LE Male Red 25Lux 2 Light Clear 25lux Inactive
6 LE Male Red 25Lux 3 Dark Red 200lux Active
6 LE Male Red 25Lux 3 Dark Red 200lux Inactive
6 LE Male Red 25Lux 3 Dark Red 25lux Inactive
6 LE Male Red 25Lux 3 Dark Red 25lux Active
6 LE Male Red 25Lux 3 Dark Clear 25lux Active
6 LE Male Red 25Lux 3 Dark Clear 200lux Inactive
6 LE Male Red 25Lux 3 Dark Clear 200lux Active
6 LE Male Red 25Lux 3 Dark Clear 25lux Inactive
6 LE Male Red 25Lux 3 Light Red 25lux Inactive
6 LE Male Red 25Lux 3 Light Red 25lux Active
6 LE Male Red 25Lux 3 Light Red 200lux Active
6 LE Male Red 25Lux 3 Light Clear 25lux Inactive
6 LE Male Red 25Lux 3 Light Clear 25lux Active
6 LE Male Red 25Lux 3 Light Clear 200lux Inactive
6 LE Male Red 25Lux 3 Light Clear 200lux Active
6 LE Male Red 25Lux 3 Light Red 200lux Inactive
LE Female Red 25Lux 1 Dark Red 200lux Active
LE Female Red 25Lux 1 Dark Red 200lux Inactive
LE Female Red 25Lux 1 Dark Red 25lux Active
LE Female Red 25Lux 1 Dark Red 25lux Inactive
LE Female Red 25Lux 1 Dark Clear 25lux Inactive
LE Female Red 25Lux 1 Dark Clear 25lux Active
LE Female Red 25Lux 1 Dark Clear 200lux Active
LE Female Red 25Lux 1 Dark Clear 200lux Inactive
LE Female Red 25Lux 1 Light Clear 25lux Active
LE Female Red 25Lux 1 Light Red 25lux Inactive
LE Female Red 25Lux 1 Light Red 25lux Active
LE Female Red 25Lux 1 Light Clear 200lux Inactive
LE Female Red 25Lux 1 Light Red 200lux Inactive
LE Female Red 25Lux 1 Light Red 200lux Active
LE Female Red 25Lux 1 Light Clear 200lux Active
LE Female Red 25Lux 1 Light Clear 25lux Inactive
LE Female Red 25Lux 2 Dark Red 200lux Active
LE Female Red 25Lux 2 Dark Red 200lux Inactive
LE Female Red 25Lux 2 Dark Red 25lux Inactive
LE Female Red 25Lux 2 Dark Red 25lux Active
LE Female Red 25Lux 2 Dark Clear 25lux Inactive
LE Female Red 25Lux 2 Dark Clear 25lux Active
LE Female Red 25Lux 2 Dark Clear 200lux Active
LE Female Red 25Lux 2 Dark Clear 200lux Inactive
LE Female Red 25Lux 2 Light Red 25lux Inactive
LE Female Red 25Lux 2 Light Red 25lux Active
LE Female Red 25Lux 2 Light Clear 200lux Inactive
LE Female Red 25Lux 2 Light Clear 25lux Inactive
LE Female Red 25Lux 2 Light Clear 200lux Active
LE Female Red 25Lux 2 Light Clear 25lux Active

LE Female Red 25Lux 2 Light Red 200lux Active
LE Female Red 25Lux 2 Light Red 200lux Inactive
7 LE Female Red 25Lux 3 Dark Red 200lux Active
7 LE Female Red 25Lux 3 Dark Red 25lux Inactive
7 LE Female Red 25Lux 3 Dark Red 25lux Active
7 LE Female Red 25Lux 3 Dark Red 200lux Inactive
7 LE Female Red 25Lux 3 Dark Clear 200lux Active
7 LE Female Red 25Lux 3 Dark Clear 200lux Inactive
7 LE Female Red 25Lux 3 Dark Clear 25lux Active
7 LE Female Red 25Lux 3 Dark Clear 25lux Inactive
7 LE Female Red 25Lux 3 Light Clear 25lux Inactive
7 LE Female Red 25Lux 3 Light Clear 25lux Active
7 LE Female Red 25Lux 3 Light Red 200lux Active
7 LE Female Red 25Lux 3 Light Red 200lux Inactive
7 LE Female Red 25Lux 3 Light Red 25lux Inactive
7 LE Female Red 25Lux 3 Light Clear 200lux Inactive
7 LE Female Red 25Lux 3 Light Clear 200lux Active
7 LE Female Red 25Lux 3 Light Red 25lux Active
LE Female Red 25Lux 1 Dark Red 200lux Active
LE Female Red 25Lux 1 Dark Red 25lux Inactive
LE Female Red 25Lux 1 Dark Red 25lux Active
LE Female Red 25Lux 1 Dark Red 200lux Inactive
LE Female Red 25Lux 1 Dark Clear 200lux Active
LE Female Red 25Lux 1 Dark Clear 200lux Inactive
LE Female Red 25Lux 1 Dark Clear 25lux Active
LE Female Red 25Lux 1 Dark Clear 25lux Inactive
LE Female Red 25Lux 1 Light Clear 25lux Active
LE Female Red 25Lux 1 Light Red 25lux Inactive
LE Female Red 25Lux 1 Light Clear 25lux Inactive
LE Female Red 25Lux 1 Light Red 25lux Active
LE Female Red 25Lux 1 Light Clear 200lux Active
LE Female Red 25Lux 1 Light Clear 200lux Inactive
LE Female Red 25Lux 1 Light Red 200lux Inactive
LE Female Red 25Lux 1 Light Red 200lux Active
LE Female Red 25Lux 2 Dark Red 25lux Active
LE Female Red 25Lux 2 Dark Red 200lux Inactive
LE Female Red 25Lux 2 Dark Red 200lux Active
LE Female Red 25Lux 2 Dark Red 25lux Inactive
LE Female Red 25Lux 2 Dark Clear 25lux Inactive
LE Female Red 25Lux 2 Dark Clear 25lux Active
LE Female Red 25Lux 2 Dark Clear 200lux Active
LE Female Red 25Lux 2 Dark Clear 200lux Inactive
LE Female Red 25Lux 2 Light Red 25lux Inactive
LE Female Red 25Lux 2 Light Red 25lux Active
LE Female Red 25Lux 2 Light Red 200lux Inactive
LE Female Red 25Lux 2 Light Red 200lux Active
LE Female Red 25Lux 2 Light Clear 25lux Active
LE Female Red 25Lux 2 Light Clear 200lux Inactive
LE Female Red 25Lux 2 Light Clear 200lux Active
LE Female Red 25Lux 2 Light Clear 25lux Inactive
8 LE Female Red 25Lux 3 Dark Red 200lux Active
8 LE Female Red 25Lux 3 Dark Red 200lux Inactive
8 LE Female Red 25Lux 3 Dark Red 25lux Active
8 LE Female Red 25Lux 3 Dark Red 25lux Inactive
8 LE Female Red 25Lux 3 Dark Clear 25lux Inactive

8 LE Female Red 25Lux 3 Dark Clear 25lux Active
8 LE Female Red 25Lux 3 Dark Clear 200lux Active
8 LE Female Red 25Lux 3 Dark Clear 200lux Inactive
8 LE Female Red 25Lux 3 Light Clear 25lux Active
8 LE Female Red 25Lux 3 Light Red 25lux Inactive
8 LE Female Red 25Lux 3 Light Red 25lux Active
8 LE Female Red 25Lux 3 Light Clear 200lux Inactive
8 LE Female Red 25Lux 3 Light Red 200lux Inactive
8 LE Female Red 25Lux 3 Light Red 200lux Active
8 LE Female Red 25Lux 3 Light Clear 200lux Active
8 LE Female Red 25Lux 3 Light Clear 25lux Inactive
LE Male Clear 200Lux 1 Dark Red 200lux Active
LE Male Clear 200Lux 1 Dark Red 200lux Inactive
LE Male Clear 200Lux 1 Dark Red 25lux Inactive
LE Male Clear 200Lux 1 Dark Red 25lux Active
LE Male Clear 200Lux 1 Dark Clear 25lux Inactive
LE Male Clear 200Lux 1 Dark Clear 25lux Active
LE Male Clear 200Lux 1 Dark Clear 200lux Active
LE Male Clear 200Lux 1 Dark Clear 200lux Inactive
LE Male Clear 200Lux 1 Light Red 25lux Inactive
LE Male Clear 200Lux 1 Light Red 25lux Active
LE Male Clear 200Lux 1 Light Red 200lux Inactive
LE Male Clear 200Lux 1 Light Clear 200lux Inactive
LE Male Clear 200Lux 1 Light Clear 25lux Active
LE Male Clear 200Lux 1 Light Clear 25lux Inactive
LE Male Clear 200Lux 1 Light Red 200lux Active
LE Male Clear 200Lux 1 Light Clear 200lux Active
LE Male Clear 200Lux 2 Dark Red 25lux Active
LE Male Clear 200Lux 2 Dark Red 200lux Inactive
LE Male Clear 200Lux 2 Dark Red 200lux Active
LE Male Clear 200Lux 2 Dark Red 25lux Inactive
LE Male Clear 200Lux 2 Dark Clear 25lux Active
LE Male Clear 200Lux 2 Dark Clear 200lux Inactive
LE Male Clear 200Lux 2 Dark Clear 200lux Active
LE Male Clear 200Lux 2 Dark Clear 25lux Inactive
LE Male Clear 200Lux 2 Light Red 25lux Inactive
LE Male Clear 200Lux 2 Light Red 25lux Active
LE Male Clear 200Lux 2 Light Clear 200lux Active
LE Male Clear 200Lux 2 Light Red 200lux Inactive
LE Male Clear 200Lux 2 Light Clear 200lux Inactive
LE Male Clear 200Lux 2 Light Clear 25lux Active
LE Male Clear 200Lux 2 Light Clear 25lux Inactive
LE Male Clear 200Lux 2 Light Red 200lux Active
9 LE Male Clear 200Lux 3 Dark Red 200lux Inactive
9 LE Male Clear 200Lux 3 Dark Red 200lux Active
9 LE Male Clear 200Lux 3 Dark Red 25lux Active
9 LE Male Clear 200Lux 3 Dark Clear 25lux Inactive
9 LE Male Clear 200Lux 3 Dark Red 25lux Inactive
9 LE Male Clear 200Lux 3 Dark Clear 25lux Active
9 LE Male Clear 200Lux 3 Dark Clear 200lux Inactive
9 LE Male Clear 200Lux 3 Dark Clear 200lux Active
9 LE Male Clear 200Lux 3 Light Red 25lux Active
9 LE Male Clear 200Lux 3 Light Clear 200lux Inactive
9 LE Male Clear 200Lux 3 Light Red 25lux Inactive
9 LE Male Clear 200Lux 3 Light Clear 200lux Active

9 LE Male Clear 200Lux 3 Light Clear 25lux Active
9 LE Male Clear 200Lux 3 Light Clear 25lux Inactive
9 LE Male Clear 200Lux 3 Light Red 200lux Active
9 LE Male Clear 200Lux 3 Light Red 200lux Inactive
LE Female Clear 200Lux 1 Dark Red 200lux Inactive
LE Female Clear 200Lux 1 Dark Red 200lux Active
LE Female Clear 200Lux 1 Dark Clear 25lux Inactive
LE Female Clear 200Lux 1 Dark Red 25lux Active
LE Female Clear 200Lux 1 Dark Clear 25lux Active
LE Female Clear 200Lux 1 Dark Clear 200lux Inactive
LE Female Clear 200Lux 1 Dark Clear 200lux Active
LE Female Clear 200Lux 1 Dark Red 25lux Inactive
LE Female Clear 200Lux 1 Light Red 25lux Inactive
LE Female Clear 200Lux 1 Light Clear 200lux Active
LE Female Clear 200Lux 1 Light Clear 200lux Inactive
LE Female Clear 200Lux 1 Light Red 25lux Active
LE Female Clear 200Lux 1 Light Clear 25lux Active
LE Female Clear 200Lux 1 Light Clear 25lux Inactive
LE Female Clear 200Lux 1 Light Red 200lux Active
LE Female Clear 200Lux 1 Light Red 200lux Inactive
LE Female Clear 200Lux 2 Dark Clear 25lux Active
LE Female Clear 200Lux 2 Dark Red 25lux Active
LE Female Clear 200Lux 2 Dark Red 200lux Inactive
LE Female Clear 200Lux 2 Dark Red 200lux Active
LE Female Clear 200Lux 2 Dark Red 25lux Inactive
LE Female Clear 200Lux 2 Dark Clear 25lux Inactive
LE Female Clear 200Lux 2 Dark Clear 200lux Inactive
LE Female Clear 200Lux 2 Dark Clear 200lux Active
LE Female Clear 200Lux 2 Light Red 25lux Inactive
LE Female Clear 200Lux 2 Light Red 25lux Active
LE Female Clear 200Lux 2 Light Clear 200lux Inactive
LE Female Clear 200Lux 2 Light Red 200lux Inactive
LE Female Clear 200Lux 2 Light Clear 200lux Active
LE Female Clear 200Lux 2 Light Clear 25lux Active
LE Female Clear 200Lux 2 Light Clear 25lux Inactive
LE Female Clear 200Lux 2 Light Red 200lux Active
10 LE Female Clear 200Lux 3 Dark Red 200lux Inactive
10 LE Female Clear 200Lux 3 Dark Red 200lux Active
10 LE Female Clear 200Lux 3 Dark Red 25lux Inactive
10 LE Female Clear 200Lux 3 Dark Red 25lux Active
10 LE Female Clear 200Lux 3 Dark Clear 200lux Active
10 LE Female Clear 200Lux 3 Dark Clear 25lux Inactive
10 LE Female Clear 200Lux 3 Dark Clear 200lux Inactive
10 LE Female Clear 200Lux 3 Dark Clear 25lux Active
10 LE Female Clear 200Lux 3 Light Clear 25lux Active
10 LE Female Clear 200Lux 3 Light Red 200lux Active
10 LE Female Clear 200Lux 3 Light Red 25lux Inactive
10 LE Female Clear 200Lux 3 Light Clear 200lux Inactive
10 LE Female Clear 200Lux 3 Light Red 200lux Inactive
10 LE Female Clear 200Lux 3 Light Clear 25lux Inactive
10 LE Female Clear 200Lux 3 Light Red 25lux Active
10 LE Female Clear 200Lux 3 Light Clear 200lux Active
LE Male Red 200Lux 1 Dark Red 200lux Active
LE Male Red 200Lux 1 Dark Red 200lux Inactive
LE Male Red 200Lux 1 Dark Red 25lux Active

LE Male Red 200Lux 1 Dark Red 25lux Inactive
LE Male Red 200Lux 1 Dark Clear 25lux Inactive
LE Male Red 200Lux 1 Dark Clear 200lux Inactive
LE Male Red 200Lux 1 Dark Clear 200lux Active
LE Male Red 200Lux 1 Dark Clear 25lux Active
LE Male Red 200Lux 1 Light Clear 25lux Active
LE Male Red 200Lux 1 Light Clear 25lux Inactive
LE Male Red 200Lux 1 Light Red 25lux Active
LE Male Red 200Lux 1 Light Clear 200lux Active
LE Male Red 200Lux 1 Light Clear 200lux Inactive
LE Male Red 200Lux 1 Light Red 200lux Inactive
LE Male Red 200Lux 1 Light Red 200lux Active
LE Male Red 200Lux 1 Light Red 25lux Inactive
LE Male Red 200Lux 2 Dark Red 200lux Inactive
LE Male Red 200Lux 2 Dark Red 25lux Active
LE Male Red 200Lux 2 Dark Red 25lux Inactive
LE Male Red 200Lux 2 Dark Red 200lux Active
LE Male Red 200Lux 2 Dark Clear 200lux Inactive
LE Male Red 200Lux 2 Dark Clear 200lux Active
LE Male Red 200Lux 2 Dark Clear 25lux Active
LE Male Red 200Lux 2 Dark Clear 25lux Inactive
LE Male Red 200Lux 2 Light Clear 25lux Inactive
LE Male Red 200Lux 2 Light Clear 25lux Active
LE Male Red 200Lux 2 Light Red 25lux Active
LE Male Red 200Lux 2 Light Clear 200lux Active
LE Male Red 200Lux 2 Light Clear 200lux Inactive
LE Male Red 200Lux 2 Light Red 200lux Inactive
LE Male Red 200Lux 2 Light Red 200lux Active
LE Male Red 200Lux 2 Light Red 25lux Inactive
11 LE Male Red 200Lux 3 Dark Red 200lux Inactive
11 LE Male Red 200Lux 3 Dark Red 25lux Active
11 LE Male Red 200Lux 3 Dark Red 25lux Inactive
11 LE Male Red 200Lux 3 Dark Red 200lux Active
11 LE Male Red 200Lux 3 Dark Clear 25lux Inactive
11 LE Male Red 200Lux 3 Dark Clear 200lux Active
11 LE Male Red 200Lux 3 Dark Clear 200lux Inactive
11 LE Male Red 200Lux 3 Dark Clear 25lux Active
11 LE Male Red 200Lux 3 Light Clear 25lux Active
11 LE Male Red 200Lux 3 Light Clear 25lux Inactive
11 LE Male Red 200Lux 3 Light Red 25lux Inactive
11 LE Male Red 200Lux 3 Light Clear 200lux Inactive
11 LE Male Red 200Lux 3 Light Red 200lux Inactive
11 LE Male Red 200Lux 3 Light Red 200lux Active
11 LE Male Red 200Lux 3 Light Red 25lux Active
11 LE Male Red 200Lux 3 Light Clear 200lux Active
LE Female Red 200Lux 1 Dark Red 200lux Active
LE Female Red 200Lux 1 Dark Red 200lux Inactive
LE Female Red 200Lux 1 Dark Red 25lux Inactive
LE Female Red 200Lux 1 Dark Red 25lux Active
LE Female Red 200Lux 1 Dark Clear 200lux Inactive
LE Female Red 200Lux 1 Dark Clear 25lux Inactive
LE Female Red 200Lux 1 Dark Clear 25lux Active
LE Female Red 200Lux 1 Dark Clear 200lux Active
LE Female Red 200Lux 1 Light Red 25lux Inactive
LE Female Red 200Lux 1 Light Red 25lux Active

LE Female Red 200Lux 1 Light Clear 25lux Active
LE Female Red 200Lux 1 Light Clear 25lux Inactive
LE Female Red 200Lux 1 Light Clear 200lux Active
LE Female Red 200Lux 1 Light Red 200lux Active
LE Female Red 200Lux 1 Light Red 200lux Inactive
LE Female Red 200Lux 1 Light Clear 200lux Inactive
LE Female Red 200Lux 2 Dark Red 200lux Active
LE Female Red 200Lux 2 Dark Red 25lux Active
LE Female Red 200Lux 2 Dark Red 25lux Inactive
LE Female Red 200Lux 2 Dark Red 200lux Inactive
LE Female Red 200Lux 2 Dark Clear 25lux Inactive
LE Female Red 200Lux 2 Dark Clear 25lux Active
LE Female Red 200Lux 2 Dark Clear 200lux Active
LE Female Red 200Lux 2 Dark Clear 200lux Inactive
LE Female Red 200Lux 2 Light Red 25lux Inactive
LE Female Red 200Lux 2 Light Red 25lux Active
LE Female Red 200Lux 2 Light Clear 200lux Inactive
LE Female Red 200Lux 2 Light Red 200lux Inactive
LE Female Red 200Lux 2 Light Red 200lux Active
LE Female Red 200Lux 2 Light Clear 25lux Active
LE Female Red 200Lux 2 Light Clear 200lux Active
LE Female Red 200Lux 2 Light Clear 25lux Inactive
12 LE Female Red 200Lux 3 Dark Red 200lux Active
12 LE Female Red 200Lux 3 Dark Red 200lux Inactive
12 LE Female Red 200Lux 3 Dark Red 25lux Active
12 LE Female Red 200Lux 3 Dark Red 25lux Inactive
12 LE Female Red 200Lux 3 Dark Clear 200lux Active
12 LE Female Red 200Lux 3 Dark Clear 25lux Active
12 LE Female Red 200Lux 3 Dark Clear 200lux Inactive
12 LE Female Red 200Lux 3 Dark Clear 25lux Inactive
12 LE Female Red 200Lux 3 Light Red 25lux Inactive
12 LE Female Red 200Lux 3 Light Red 25lux Active
12 LE Female Red 200Lux 3 Light Clear 200lux Active
12 LE Female Red 200Lux 3 Light Red 200lux Inactive
12 LE Female Red 200Lux 3 Light Red 200lux Active
12 LE Female Red 200Lux 3 Light Clear 25lux Inactive
12 LE Female Red 200Lux 3 Light Clear 25lux Active
12 LE Female Red 200Lux 3 Light Clear 200lux Inactive
LE Male Clear 200Lux 1 Dark Clear 200lux Active
LE Male Clear 200Lux 1 Dark Red 25lux Inactive
LE Male Clear 200Lux 1 Dark Red 25lux Active
LE Male Clear 200Lux 1 Dark Red 200lux Inactive
LE Male Clear 200Lux 1 Dark Red 200lux Active
LE Male Clear 200Lux 1 Dark Clear 200lux Inactive
LE Male Clear 200Lux 1 Dark Clear 25lux Inactive
LE Male Clear 200Lux 1 Dark Clear 25lux Active
LE Male Clear 200Lux 1 Light Red 25lux Inactive
LE Male Clear 200Lux 1 Light Clear 25lux Active
LE Male Clear 200Lux 1 Light Red 200lux Inactive
LE Male Clear 200Lux 1 Light Clear 25lux Inactive
LE Male Clear 200Lux 1 Light Red 200lux Active
LE Male Clear 200Lux 1 Light Red 25lux Active
LE Male Clear 200Lux 1 Light Clear 200lux Inactive
LE Male Clear 200Lux 1 Light Clear 200lux Active
LE Male Clear 200Lux 2 Dark Red 25lux Active

LE Male Clear 200Lux 2 Dark Red 25lux Inactive
LE Male Clear 200Lux 2 Dark Red 200lux Inactive
LE Male Clear 200Lux 2 Dark Red 200lux Active
LE Male Clear 200Lux 2 Dark Clear 25lux Inactive
LE Male Clear 200Lux 2 Dark Clear 25lux Active
LE Male Clear 200Lux 2 Dark Clear 200lux Active
LE Male Clear 200Lux 2 Dark Clear 200lux Inactive
LE Male Clear 200Lux 2 Light Red 25lux Inactive
LE Male Clear 200Lux 2 Light Clear 25lux Active
LE Male Clear 200Lux 2 Light Clear 200lux Inactive
LE Male Clear 200Lux 2 Light Red 200lux Inactive
LE Male Clear 200Lux 2 Light Red 200lux Active
LE Male Clear 200Lux 2 Light Clear 25lux Inactive
LE Male Clear 200Lux 2 Light Red 25lux Active
LE Male Clear 200Lux 2 Light Clear 200lux Active
13 LE Male Clear 200Lux 3 Dark Red 200lux Active
13 LE Male Clear 200Lux 3 Dark Red 200lux Inactive
13 LE Male Clear 200Lux 3 Dark Clear 25lux Active
13 LE Male Clear 200Lux 3 Dark Red 25lux Active
13 LE Male Clear 200Lux 3 Dark Red 25lux Inactive
13 LE Male Clear 200Lux 3 Dark Clear 25lux Inactive
13 LE Male Clear 200Lux 3 Dark Clear 200lux Inactive
13 LE Male Clear 200Lux 3 Dark Clear 200lux Active
13 LE Male Clear 200Lux 3 Light Red 25lux Inactive
13 LE Male Clear 200Lux 3 Light Clear 200lux Active
13 LE Male Clear 200Lux 3 Light Clear 200lux Inactive
13 LE Male Clear 200Lux 3 Light Red 200lux Inactive
13 LE Male Clear 200Lux 3 Light Red 200lux Active
13 LE Male Clear 200Lux 3 Light Clear 25lux Inactive
13 LE Male Clear 200Lux 3 Light Clear 25lux Active
13 LE Male Clear 200Lux 3 Light Red 25lux Active
LE Male Clear 200Lux 1 Dark Red 25lux Active
LE Male Clear 200Lux 1 Dark Red 25lux Inactive
LE Male Clear 200Lux 1 Dark Red 200lux Active
LE Male Clear 200Lux 1 Dark Red 200lux Inactive
LE Male Clear 200Lux 1 Dark Clear 200lux Inactive
LE Male Clear 200Lux 1 Dark Clear 25lux Inactive
LE Male Clear 200Lux 1 Dark Clear 25lux Active
LE Male Clear 200Lux 1 Dark Clear 200lux Active
LE Male Clear 200Lux 1 Light Red 200lux Active
LE Male Clear 200Lux 1 Light Red 25lux Inactive
LE Male Clear 200Lux 1 Light Clear 25lux Active
LE Male Clear 200Lux 1 Light Clear 25lux Inactive
LE Male Clear 200Lux 1 Light Red 25lux Active
LE Male Clear 200Lux 1 Light Red 200lux Inactive
LE Male Clear 200Lux 1 Light Clear 200lux Inactive
LE Male Clear 200Lux 1 Light Clear 200lux Active
LE Male Clear 200Lux 2 Dark Red 200lux Active
LE Male Clear 200Lux 2 Dark Red 25lux Inactive
LE Male Clear 200Lux 2 Dark Red 25lux Active
LE Male Clear 200Lux 2 Dark Red 200lux Inactive
LE Male Clear 200Lux 2 Dark Clear 200lux Active
LE Male Clear 200Lux 2 Dark Clear 25lux Inactive
LE Male Clear 200Lux 2 Dark Clear 25lux Active
LE Male Clear 200Lux 2 Dark Clear 200lux Inactive

LE Male Clear 200Lux 2 Light Red 25lux Inactive
LE Male Clear 200Lux 2 Light Red 25lux Active
LE Male Clear 200Lux 2 Light Clear 25lux Inactive
LE Male Clear 200Lux 2 Light Clear 200lux Active
LE Male Clear 200Lux 2 Light Red 200lux Active
LE Male Clear 200Lux 2 Light Red 200lux Inactive
LE Male Clear 200Lux 2 Light Clear 200lux Inactive
LE Male Clear 200Lux 2 Light Clear 25lux Active
14 LE Male Clear 200Lux 3 Dark Red 200lux Active
14 LE Male Clear 200Lux 3 Dark Red 200lux Inactive
14 LE Male Clear 200Lux 3 Dark Red 25lux Inactive
14 LE Male Clear 200Lux 3 Dark Red 25lux Active
14 LE Male Clear 200Lux 3 Dark Clear 200lux Active
14 LE Male Clear 200Lux 3 Dark Clear 25lux Inactive
14 LE Male Clear 200Lux 3 Dark Clear 200lux Inactive
14 LE Male Clear 200Lux 3 Dark Clear 25lux Active
14 LE Male Clear 200Lux 3 Light Red 25lux Inactive
14 LE Male Clear 200Lux 3 Light Red 200lux Active
14 LE Male Clear 200Lux 3 Light Clear 25lux Active
14 LE Male Clear 200Lux 3 Light Clear 200lux Inactive
14 LE Male Clear 200Lux 3 Light Red 200lux Inactive
14 LE Male Clear 200Lux 3 Light Red 25lux Active
14 LE Male Clear 200Lux 3 Light Clear 25lux Inactive
14 LE Male Clear 200Lux 3 Light Clear 200lux Active
LE Female Clear 200Lux 1 Dark Red 200lux Active
LE Female Clear 200Lux 1 Dark Red 200lux Inactive
LE Female Clear 200Lux 1 Dark Red 25lux Inactive
LE Female Clear 200Lux 1 Dark Red 25lux Active
LE Female Clear 200Lux 1 Dark Clear 25lux Inactive
LE Female Clear 200Lux 1 Dark Clear 25lux Active
LE Female Clear 200Lux 1 Dark Clear 200lux Active
LE Female Clear 200Lux 1 Dark Clear 200lux Inactive
LE Female Clear 200Lux 1 Light Red 25lux Inactive
LE Female Clear 200Lux 1 Light Red 25lux Active
LE Female Clear 200Lux 1 Light Clear 200lux Inactive
LE Female Clear 200Lux 1 Light Clear 200lux Active
LE Female Clear 200Lux 1 Light Clear 25lux Active
LE Female Clear 200Lux 1 Light Clear 25lux Inactive
LE Female Clear 200Lux 1 Light Red 200lux Active
LE Female Clear 200Lux 1 Light Red 200lux Inactive
LE Female Clear 200Lux 2 Dark Clear 25lux Active
LE Female Clear 200Lux 2 Dark Red 25lux Active
LE Female Clear 200Lux 2 Dark Red 200lux Inactive
LE Female Clear 200Lux 2 Dark Red 200lux Active
LE Female Clear 200Lux 2 Dark Red 25lux Inactive
LE Female Clear 200Lux 2 Dark Clear 25lux Inactive
LE Female Clear 200Lux 2 Dark Clear 200lux Inactive
LE Female Clear 200Lux 2 Dark Clear 200lux Active
LE Female Clear 200Lux 2 Light Red 25lux Inactive
LE Female Clear 200Lux 2 Light Red 25lux Active
LE Female Clear 200Lux 2 Light Clear 200lux Inactive
LE Female Clear 200Lux 2 Light Red 200lux Active
LE Female Clear 200Lux 2 Light Clear 25lux Inactive
LE Female Clear 200Lux 2 Light Clear 25lux Active
LE Female Clear 200Lux 2 Light Red 200lux Inactive

LE Female Clear 200Lux 2 Light Clear 200lux Active
15 LE Female Clear 200Lux 3 Dark Red 200lux Inactive
15 LE Female Clear 200Lux 3 Dark Red 25lux Active
15 LE Female Clear 200Lux 3 Dark Red 25lux Inactive
15 LE Female Clear 200Lux 3 Dark Clear 25lux Active
15 LE Female Clear 200Lux 3 Dark Red 200lux Active
15 LE Female Clear 200Lux 3 Dark Clear 200lux Inactive
15 LE Female Clear 200Lux 3 Dark Clear 200lux Active
15 LE Female Clear 200Lux 3 Dark Clear 25lux Inactive
15 LE Female Clear 200Lux 3 Light Red 25lux Inactive
15 LE Female Clear 200Lux 3 Light Clear 25lux Inactive
15 LE Female Clear 200Lux 3 Light Red 25lux Active
15 LE Female Clear 200Lux 3 Light Clear 25lux Active
15 LE Female Clear 200Lux 3 Light Clear 200lux Active
15 LE Female Clear 200Lux 3 Light Clear 200lux Inactive
15 LE Female Clear 200Lux 3 Light Red 200lux Inactive
15 LE Female Clear 200Lux 3 Light Red 200lux Active
LE Female Clear 200Lux 1 Dark Red 200lux Inactive
LE Female Clear 200Lux 1 Dark Red 200lux Active
LE Female Clear 200Lux 1 Dark Red 25lux Active
LE Female Clear 200Lux 1 Dark Red 25lux Inactive
LE Female Clear 200Lux 1 Dark Clear 25lux Inactive
LE Female Clear 200Lux 1 Dark Clear 25lux Active
LE Female Clear 200Lux 1 Dark Clear 200lux Active
LE Female Clear 200Lux 1 Dark Clear 200lux Inactive
LE Female Clear 200Lux 1 Light Clear 200lux Active
LE Female Clear 200Lux 1 Light Red 25lux Inactive
LE Female Clear 200Lux 1 Light Clear 200lux Inactive
LE Female Clear 200Lux 1 Light Red 25lux Active
LE Female Clear 200Lux 1 Light Clear 25lux Active
LE Female Clear 200Lux 1 Light Clear 25lux Inactive
LE Female Clear 200Lux 1 Light Red 200lux Active
LE Female Clear 200Lux 1 Light Red 200lux Inactive
LE Female Clear 200Lux 2 Dark Red 200lux Inactive
LE Female Clear 200Lux 2 Dark Red 200lux Active
LE Female Clear 200Lux 2 Dark Clear 25lux Inactive
LE Female Clear 200Lux 2 Dark Red 25lux Inactive
LE Female Clear 200Lux 2 Dark Red 25lux Active
LE Female Clear 200Lux 2 Dark Clear 25lux Active
LE Female Clear 200Lux 2 Dark Clear 200lux Inactive
LE Female Clear 200Lux 2 Dark Clear 200lux Active
LE Female Clear 200Lux 2 Light Red 25lux Active
LE Female Clear 200Lux 2 Light Red 25lux Inactive
LE Female Clear 200Lux 2 Light Clear 200lux Inactive
LE Female Clear 200Lux 2 Light Red 200lux Inactive
LE Female Clear 200Lux 2 Light Clear 200lux Active
LE Female Clear 200Lux 2 Light Clear 25lux Active
LE Female Clear 200Lux 2 Light Clear 25lux Inactive
LE Female Clear 200Lux 2 Light Red 200lux Active
16 LE Female Clear 200Lux 3 Dark Red 200lux Active
16 LE Female Clear 200Lux 3 Dark Red 25lux Inactive
16 LE Female Clear 200Lux 3 Dark Red 25lux Active
16 LE Female Clear 200Lux 3 Dark Red 200lux Inactive
16 LE Female Clear 200Lux 3 Dark Clear 25lux Inactive
16 LE Female Clear 200Lux 3 Dark Clear 25lux Active

16 LE Female Clear 200Lux 3 Dark Clear 200lux Active
16 LE Female Clear 200Lux 3 Dark Clear 200lux Inactive
16 LE Female Clear 200Lux 3 Light Clear 25lux Active
16 LE Female Clear 200Lux 3 Light Red 25lux Inactive
16 LE Female Clear 200Lux 3 Light Clear 25lux Inactive
16 LE Female Clear 200Lux 3 Light Clear 200lux Active
16 LE Female Clear 200Lux 3 Light Clear 200lux Inactive
16 LE Female Clear 200Lux 3 Light Red 200lux Inactive
16 LE Female Clear 200Lux 3 Light Red 200lux Active
16 LE Female Clear 200Lux 3 Light Red 25lux Active
LE Male Red 200Lux 1 Dark Red 200lux Inactive
LE Male Red 200Lux 1 Dark Red 25lux Active
LE Male Red 200Lux 1 Dark Red 25lux Inactive
LE Male Red 200Lux 1 Dark Red 200lux Active
LE Male Red 200Lux 1 Dark Clear 200lux Inactive
LE Male Red 200Lux 1 Dark Clear 25lux Inactive
LE Male Red 200Lux 1 Dark Clear 25lux Active
LE Male Red 200Lux 1 Dark Clear 200lux Active
LE Male Red 200Lux 1 Light Clear 25lux Active
LE Male Red 200Lux 1 Light Red 25lux Inactive
LE Male Red 200Lux 1 Light Red 25lux Active
LE Male Red 200Lux 1 Light Clear 200lux Inactive
LE Male Red 200Lux 1 Light Red 200lux Inactive
LE Male Red 200Lux 1 Light Red 200lux Active
LE Male Red 200Lux 1 Light Clear 200lux Active
LE Male Red 200Lux 1 Light Clear 25lux Inactive
LE Male Red 200Lux 2 Dark Red 200lux Active
LE Male Red 200Lux 2 Dark Red 200lux Inactive
LE Male Red 200Lux 2 Dark Red 25lux Active
LE Male Red 200Lux 2 Dark Red 25lux Inactive
LE Male Red 200Lux 2 Dark Clear 25lux Inactive
LE Male Red 200Lux 2 Dark Clear 25lux Active
LE Male Red 200Lux 2 Dark Clear 200lux Active
LE Male Red 200Lux 2 Dark Clear 200lux Inactive
LE Male Red 200Lux 2 Light Red 25lux Inactive
LE Male Red 200Lux 2 Light Red 25lux Active
LE Male Red 200Lux 2 Light Red 200lux Inactive
LE Male Red 200Lux 2 Light Red 200lux Active
LE Male Red 200Lux 2 Light Clear 25lux Active
LE Male Red 200Lux 2 Light Clear 200lux Inactive
LE Male Red 200Lux 2 Light Clear 200lux Active
LE Male Red 200Lux 2 Light Clear 25lux Inactive
17 LE Male Red 200Lux 3 Dark Red 200lux Active
17 LE Male Red 200Lux 3 Dark Red 200lux Inactive
17 LE Male Red 200Lux 3 Dark Red 25lux Active
17 LE Male Red 200Lux 3 Dark Red 25lux Inactive
17 LE Male Red 200Lux 3 Dark Clear 25lux Inactive
17 LE Male Red 200Lux 3 Dark Clear 25lux Active
17 LE Male Red 200Lux 3 Dark Clear 200lux Inactive
17 LE Male Red 200Lux 3 Dark Clear 200lux Active
17 LE Male Red 200Lux 3 Light Clear 25lux Active
17 LE Male Red 200Lux 3 Light Red 25lux Inactive
17 LE Male Red 200Lux 3 Light Red 25lux Active
17 LE Male Red 200Lux 3 Light Clear 200lux Inactive
17 LE Male Red 200Lux 3 Light Red 200lux Inactive

17 LE Male Red 200Lux 3 Light Red 200lux Active
17 LE Male Red 200Lux 3 Light Clear 200lux Active
17 LE Male Red 200Lux 3 Light Clear 25lux Inactive
LE Male Red 200Lux 1 Dark Red 200lux Active
LE Male Red 200Lux 1 Dark Red 200lux Inactive
LE Male Red 200Lux 1 Dark Red 25lux Inactive
LE Male Red 200Lux 1 Dark Red 25lux Active
LE Male Red 200Lux 1 Dark Clear 25lux Inactive
LE Male Red 200Lux 1 Dark Clear 25lux Active
LE Male Red 200Lux 1 Dark Clear 200lux Active
LE Male Red 200Lux 1 Dark Clear 200lux Inactive
LE Male Red 200Lux 1 Light Red 25lux Inactive
LE Male Red 200Lux 1 Light Red 25lux Active
LE Male Red 200Lux 1 Light Clear 200lux Active
LE Male Red 200Lux 1 Light Red 200lux Inactive
LE Male Red 200Lux 1 Light Clear 200lux Inactive
LE Male Red 200Lux 1 Light Clear 25lux Active
LE Male Red 200Lux 1 Light Clear 25lux Inactive
LE Male Red 200Lux 1 Light Red 200lux Active
LE Male Red 200Lux 2 Dark Red 25lux Active
LE Male Red 200Lux 2 Dark Red 200lux Inactive
LE Male Red 200Lux 2 Dark Red 200lux Active
LE Male Red 200Lux 2 Dark Red 25lux Inactive
LE Male Red 200Lux 2 Dark Clear 25lux Active
LE Male Red 200Lux 2 Dark Clear 200lux Inactive
LE Male Red 200Lux 2 Dark Clear 200lux Active
LE Male Red 200Lux 2 Dark Clear 25lux Inactive
LE Male Red 200Lux 2 Light Red 25lux Inactive
LE Male Red 200Lux 2 Light Red 25lux Active
LE Male Red 200Lux 2 Light Clear 200lux Active
LE Male Red 200Lux 2 Light Red 200lux Inactive
LE Male Red 200Lux 2 Light Red 200lux Active
LE Male Red 200Lux 2 Light Clear 25lux Active
LE Male Red 200Lux 2 Light Clear 200lux Inactive
LE Male Red 200Lux 2 Light Clear 25lux Inactive
18 LE Male Red 200Lux 3 Dark Red 200lux Active
18 LE Male Red 200Lux 3 Dark Red 200lux Inactive
18 LE Male Red 200Lux 3 Dark Red 25lux Inactive
18 LE Male Red 200Lux 3 Dark Red 25lux Active
18 LE Male Red 200Lux 3 Dark Clear 25lux Active
18 LE Male Red 200Lux 3 Dark Clear 200lux Inactive
18 LE Male Red 200Lux 3 Dark Clear 200lux Active
18 LE Male Red 200Lux 3 Dark Clear 25lux Inactive
18 LE Male Red 200Lux 3 Light Red 25lux Inactive
18 LE Male Red 200Lux 3 Light Red 25lux Active
18 LE Male Red 200Lux 3 Light Red 200lux Active
18 LE Male Red 200Lux 3 Light Clear 25lux Inactive
18 LE Male Red 200Lux 3 Light Clear 25lux Active
18 LE Male Red 200Lux 3 Light Clear 200lux Inactive
18 LE Male Red 200Lux 3 Light Clear 200lux Active
18 LE Male Red 200Lux 3 Light Red 200lux Inactive
LE Female Red 200Lux 1 Dark Red 200lux Inactive
LE Female Red 200Lux 1 Dark Red 25lux Active
LE Female Red 200Lux 1 Dark Red 25lux Inactive
LE Female Red 200Lux 1 Dark Red 200lux Active

LE Female Red 200Lux 1 Dark Clear 25lux Inactive
LE Female Red 200Lux 1 Dark Clear 25lux Active
LE Female Red 200Lux 1 Dark Clear 200lux Inactive
LE Female Red 200Lux 1 Dark Clear 200lux Active
LE Female Red 200Lux 1 Light Clear 25lux Active
LE Female Red 200Lux 1 Light Red 25lux Inactive
LE Female Red 200Lux 1 Light Red 25lux Active
LE Female Red 200Lux 1 Light Clear 200lux Inactive
LE Female Red 200Lux 1 Light Red 200lux Inactive
LE Female Red 200Lux 1 Light Red 200lux Active
LE Female Red 200Lux 1 Light Clear 200lux Active
LE Female Red 200Lux 1 Light Clear 25lux Inactive
LE Female Red 200Lux 2 Dark Red 200lux Active
LE Female Red 200Lux 2 Dark Red 200lux Inactive
LE Female Red 200Lux 2 Dark Red 25lux Inactive
LE Female Red 200Lux 2 Dark Red 25lux Active
LE Female Red 200Lux 2 Dark Clear 25lux Inactive
LE Female Red 200Lux 2 Dark Clear 25lux Active
LE Female Red 200Lux 2 Dark Clear 200lux Active
LE Female Red 200Lux 2 Dark Clear 200lux Inactive
LE Female Red 200Lux 2 Light Red 25lux Inactive
LE Female Red 200Lux 2 Light Red 25lux Active
LE Female Red 200Lux 2 Light Clear 200lux Inactive
LE Female Red 200Lux 2 Light Clear 25lux Inactive
LE Female Red 200Lux 2 Light Clear 200lux Active
LE Female Red 200Lux 2 Light Clear 25lux Active
LE Female Red 200Lux 2 Light Red 200lux Active
LE Female Red 200Lux 2 Light Red 200lux Inactive
19 LE Female Red 200Lux 3 Dark Red 200lux Active
19 LE Female Red 200Lux 3 Dark Red 25lux Inactive
19 LE Female Red 200Lux 3 Dark Red 25lux Active
19 LE Female Red 200Lux 3 Dark Red 200lux Inactive
19 LE Female Red 200Lux 3 Dark Clear 200lux Active
19 LE Female Red 200Lux 3 Dark Clear 200lux Inactive
19 LE Female Red 200Lux 3 Dark Clear 25lux Active
19 LE Female Red 200Lux 3 Dark Clear 25lux Inactive
19 LE Female Red 200Lux 3 Light Clear 25lux Inactive
19 LE Female Red 200Lux 3 Light Clear 25lux Active
19 LE Female Red 200Lux 3 Light Red 200lux Active
19 LE Female Red 200Lux 3 Light Red 200lux Inactive
19 LE Female Red 200Lux 3 Light Red 25lux Inactive
19 LE Female Red 200Lux 3 Light Clear 200lux Inactive
19 LE Female Red 200Lux 3 Light Clear 200lux Active
19 LE Female Red 200Lux 3 Light Red 25lux Active
LE Female Red 200Lux 1 Dark Red 200lux Active
LE Female Red 200Lux 1 Dark Red 25lux Inactive
LE Female Red 200Lux 1 Dark Red 25lux Active
LE Female Red 200Lux 1 Dark Red 200lux Inactive
LE Female Red 200Lux 1 Dark Clear 200lux Active
LE Female Red 200Lux 1 Dark Clear 200lux Inactive
LE Female Red 200Lux 1 Dark Clear 25lux Active
LE Female Red 200Lux 1 Dark Clear 25lux Inactive
LE Female Red 200Lux 1 Light Clear 25lux Active
LE Female Red 200Lux 1 Light Red 25lux Inactive
LE Female Red 200Lux 1 Light Clear 25lux Inactive

LE Female Red 200Lux 1 Light Red 25lux Active
LE Female Red 200Lux 1 Light Clear 200lux Active
LE Female Red 200Lux 1 Light Clear 200lux Inactive
LE Female Red 200Lux 1 Light Red 200lux Inactive
LE Female Red 200Lux 1 Light Red 200lux Active
LE Female Red 200Lux 2 Dark Red 25lux Active
LE Female Red 200Lux 2 Dark Red 200lux Inactive
LE Female Red 200Lux 2 Dark Red 200lux Active
LE Female Red 200Lux 2 Dark Red 25lux Inactive
LE Female Red 200Lux 2 Dark Clear 25lux Inactive
LE Female Red 200Lux 2 Dark Clear 25lux Active
LE Female Red 200Lux 2 Dark Clear 200lux Active
LE Female Red 200Lux 2 Dark Clear 200lux Inactive
LE Female Red 200Lux 2 Light Red 25lux Inactive
LE Female Red 200Lux 2 Light Red 25lux Active
LE Female Red 200Lux 2 Light Red 200lux Inactive
LE Female Red 200Lux 2 Light Red 200lux Active
LE Female Red 200Lux 2 Light Clear 25lux Active
LE Female Red 200Lux 2 Light Clear 200lux Inactive
LE Female Red 200Lux 2 Light Clear 200lux Active
LE Female Red 200Lux 2 Light Clear 25lux Inactive
20 LE Female Red 200Lux 3 Dark Red 200lux Active
20 LE Female Red 200Lux 3 Dark Red 200lux Inactive
20 LE Female Red 200Lux 3 Dark Red 25lux Active
20 LE Female Red 200Lux 3 Dark Red 25lux Inactive
20 LE Female Red 200Lux 3 Dark Clear 25lux Inactive
20 LE Female Red 200Lux 3 Dark Clear 25lux Active
20 LE Female Red 200Lux 3 Dark Clear 200lux Active
20 LE Female Red 200Lux 3 Dark Clear 200lux Inactive
20 LE Female Red 200Lux 3 Light Clear 25lux Active
20 LE Female Red 200Lux 3 Light Red 25lux Inactive
20 LE Female Red 200Lux 3 Light Red 25lux Active
20 LE Female Red 200Lux 3 Light Clear 200lux Inactive
20 LE Female Red 200Lux 3 Light Red 200lux Inactive
20 LE Female Red 200Lux 3 Light Red 200lux Active
20 LE Female Red 200Lux 3 Light Clear 200lux Active
20 LE Female Red 200Lux 3 Light Clear 25lux Inactive
LE Male Clear 25Lux 1 Dark Red 200lux Active
LE Male Clear 25Lux 1 Dark Red 200lux Inactive
LE Male Clear 25Lux 1 Dark Red 25lux Inactive
LE Male Clear 25Lux 1 Dark Red 25lux Active
LE Male Clear 25Lux 1 Dark Clear 25lux Inactive
LE Male Clear 25Lux 1 Dark Clear 25lux Active
LE Male Clear 25Lux 1 Dark Clear 200lux Active
LE Male Clear 25Lux 1 Dark Clear 200lux Inactive
LE Male Clear 25Lux 1 Light Red 25lux Inactive
LE Male Clear 25Lux 1 Light Red 25lux Active
LE Male Clear 25Lux 1 Light Red 200lux Inactive
LE Male Clear 25Lux 1 Light Clear 200lux Inactive
LE Male Clear 25Lux 1 Light Clear 25lux Active
LE Male Clear 25Lux 1 Light Clear 25lux Inactive
LE Male Clear 25Lux 1 Light Red 200lux Active
LE Male Clear 25Lux 1 Light Clear 200lux Active
LE Male Clear 25Lux 2 Dark Red 25lux Active
LE Male Clear 25Lux 2 Dark Red 200lux Inactive

LE Male Clear 25Lux 2 Dark Red 200lux Active
LE Male Clear 25Lux 2 Dark Red 25lux Inactive
LE Male Clear 25Lux 2 Dark Clear 25lux Active
LE Male Clear 25Lux 2 Dark Clear 200lux Inactive
LE Male Clear 25Lux 2 Dark Clear 200lux Active
LE Male Clear 25Lux 2 Dark Clear 25lux Inactive
LE Male Clear 25Lux 2 Light Red 25lux Inactive
LE Male Clear 25Lux 2 Light Red 25lux Active
LE Male Clear 25Lux 2 Light Clear 200lux Active
LE Male Clear 25Lux 2 Light Red 200lux Inactive
LE Male Clear 25Lux 2 Light Clear 200lux Inactive
LE Male Clear 25Lux 2 Light Clear 25lux Active
LE Male Clear 25Lux 2 Light Clear 25lux Inactive
LE Male Clear 25Lux 2 Light Red 200lux Active
21 LE Male Clear 25Lux 3 Dark Red 200lux Inactive
21 LE Male Clear 25Lux 3 Dark Red 200lux Active
21 LE Male Clear 25Lux 3 Dark Red 25lux Active
21 LE Male Clear 25Lux 3 Dark Clear 25lux Inactive
21 LE Male Clear 25Lux 3 Dark Red 25lux Inactive
21 LE Male Clear 25Lux 3 Dark Clear 25lux Active
21 LE Male Clear 25Lux 3 Dark Clear 200lux Inactive
21 LE Male Clear 25Lux 3 Dark Clear 200lux Active
21 LE Male Clear 25Lux 3 Light Red 25lux Active
21 LE Male Clear 25Lux 3 Light Clear 200lux Inactive
21 LE Male Clear 25Lux 3 Light Red 25lux Inactive
21 LE Male Clear 25Lux 3 Light Clear 200lux Active
21 LE Male Clear 25Lux 3 Light Clear 25lux Active
21 LE Male Clear 25Lux 3 Light Clear 25lux Inactive
21 LE Male Clear 25Lux 3 Light Red 200lux Active
21 LE Male Clear 25Lux 3 Light Red 200lux Inactive
LE Female Clear 25Lux 1 Dark Red 200lux Inactive
LE Female Clear 25Lux 1 Dark Red 200lux Active
LE Female Clear 25Lux 1 Dark Clear 25lux Inactive
LE Female Clear 25Lux 1 Dark Red 25lux Active
LE Female Clear 25Lux 1 Dark Clear 25lux Active
LE Female Clear 25Lux 1 Dark Clear 200lux Inactive
LE Female Clear 25Lux 1 Dark Clear 200lux Active
LE Female Clear 25Lux 1 Dark Red 25lux Inactive
LE Female Clear 25Lux 1 Light Red 25lux Inactive
LE Female Clear 25Lux 1 Light Clear 200lux Active
LE Female Clear 25Lux 1 Light Clear 200lux Inactive
LE Female Clear 25Lux 1 Light Red 25lux Active
LE Female Clear 25Lux 1 Light Clear 25lux Active
LE Female Clear 25Lux 1 Light Clear 25lux Inactive
LE Female Clear 25Lux 1 Light Red 200lux Active
LE Female Clear 25Lux 1 Light Red 200lux Inactive
LE Female Clear 25Lux 2 Dark Clear 25lux Active
LE Female Clear 25Lux 2 Dark Red 25lux Active
LE Female Clear 25Lux 2 Dark Red 200lux Inactive
LE Female Clear 25Lux 2 Dark Red 200lux Active
LE Female Clear 25Lux 2 Dark Red 25lux Inactive
LE Female Clear 25Lux 2 Dark Clear 25lux Inactive
LE Female Clear 25Lux 2 Dark Clear 200lux Inactive
LE Female Clear 25Lux 2 Dark Clear 200lux Active
LE Female Clear 25Lux 2 Light Red 25lux Inactive

LE Female Clear 25Lux 2 Light Red 25lux Active
LE Female Clear 25Lux 2 Light Clear 200lux Inactive
LE Female Clear 25Lux 2 Light Red 200lux Inactive
LE Female Clear 25Lux 2 Light Clear 200lux Active
LE Female Clear 25Lux 2 Light Clear 25lux Active
LE Female Clear 25Lux 2 Light Clear 25lux Inactive
LE Female Clear 25Lux 2 Light Red 200lux Active
22 LE Female Clear 25Lux 3 Dark Red 200lux Inactive
22 LE Female Clear 25Lux 3 Dark Red 200lux Active
22 LE Female Clear 25Lux 3 Dark Red 25lux Inactive
22 LE Female Clear 25Lux 3 Dark Red 25lux Active
22 LE Female Clear 25Lux 3 Dark Clear 200lux Active
22 LE Female Clear 25Lux 3 Dark Clear 25lux Inactive
22 LE Female Clear 25Lux 3 Dark Clear 200lux Inactive
22 LE Female Clear 25Lux 3 Dark Clear 25lux Active
22 LE Female Clear 25Lux 3 Light Clear 25lux Active
22 LE Female Clear 25Lux 3 Light Red 200lux Active
22 LE Female Clear 25Lux 3 Light Red 25lux Inactive
22 LE Female Clear 25Lux 3 Light Clear 200lux Inactive
22 LE Female Clear 25Lux 3 Light Red 200lux Inactive
22 LE Female Clear 25Lux 3 Light Clear 25lux Inactive
22 LE Female Clear 25Lux 3 Light Red 25lux Active
22 LE Female Clear 25Lux 3 Light Clear 200lux Active
LE Male Red 25Lux 1 Dark Red 200lux Active
LE Male Red 25Lux 1 Dark Red 200lux Inactive
LE Male Red 25Lux 1 Dark Red 25lux Active
LE Male Red 25Lux 1 Dark Red 25lux Inactive
LE Male Red 25Lux 1 Dark Clear 25lux Inactive
LE Male Red 25Lux 1 Dark Clear 200lux Inactive
LE Male Red 25Lux 1 Dark Clear 200lux Active
LE Male Red 25Lux 1 Dark Clear 25lux Active
LE Male Red 25Lux 1 Light Clear 25lux Active
LE Male Red 25Lux 1 Light Clear 25lux Inactive
LE Male Red 25Lux 1 Light Red 25lux Active
LE Male Red 25Lux 1 Light Clear 200lux Active
LE Male Red 25Lux 1 Light Clear 200lux Inactive
LE Male Red 25Lux 1 Light Red 200lux Inactive
LE Male Red 25Lux 1 Light Red 200lux Active
LE Male Red 25Lux 1 Light Red 25lux Inactive
LE Male Red 25Lux 2 Dark Red 200lux Inactive
LE Male Red 25Lux 2 Dark Red 25lux Active
LE Male Red 25Lux 2 Dark Red 25lux Inactive
LE Male Red 25Lux 2 Dark Red 200lux Active
LE Male Red 25Lux 2 Dark Clear 200lux Inactive
LE Male Red 25Lux 2 Dark Clear 200lux Active
LE Male Red 25Lux 2 Dark Clear 25lux Active
LE Male Red 25Lux 2 Dark Clear 25lux Inactive
LE Male Red 25Lux 2 Light Clear 25lux Inactive
LE Male Red 25Lux 2 Light Clear 25lux Active
LE Male Red 25Lux 2 Light Red 25lux Active
LE Male Red 25Lux 2 Light Clear 200lux Active
LE Male Red 25Lux 2 Light Clear 200lux Inactive
LE Male Red 25Lux 2 Light Red 200lux Inactive
LE Male Red 25Lux 2 Light Red 200lux Active
LE Male Red 25Lux 2 Light Red 25lux Inactive

23 LE Male Red 25Lux 3 Dark Red 200lux Inactive
23 LE Male Red 25Lux 3 Dark Red 25lux Active
23 LE Male Red 25Lux 3 Dark Red 25lux Inactive
23 LE Male Red 25Lux 3 Dark Red 200lux Active
23 LE Male Red 25Lux 3 Dark Clear 25lux Inactive
23 LE Male Red 25Lux 3 Dark Clear 200lux Active
23 LE Male Red 25Lux 3 Dark Clear 200lux Inactive
23 LE Male Red 25Lux 3 Dark Clear 25lux Active
23 LE Male Red 25Lux 3 Light Clear 25lux Active
23 LE Male Red 25Lux 3 Light Clear 25lux Inactive
23 LE Male Red 25Lux 3 Light Red 25lux Inactive
23 LE Male Red 25Lux 3 Light Clear 200lux Inactive
23 LE Male Red 25Lux 3 Light Red 200lux Inactive
23 LE Male Red 25Lux 3 Light Red 200lux Active
23 LE Male Red 25Lux 3 Light Red 25lux Active
23 LE Male Red 25Lux 3 Light Clear 200lux Active
LE Female Red 25Lux 1 Dark Red 200lux Active
LE Female Red 25Lux 1 Dark Red 200lux Inactive
LE Female Red 25Lux 1 Dark Red 25lux Inactive
LE Female Red 25Lux 1 Dark Red 25lux Active
LE Female Red 25Lux 1 Dark Clear 200lux Inactive
LE Female Red 25Lux 1 Dark Clear 25lux Inactive
LE Female Red 25Lux 1 Dark Clear 25lux Active
LE Female Red 25Lux 1 Dark Clear 200lux Active
LE Female Red 25Lux 1 Light Red 25lux Inactive
LE Female Red 25Lux 1 Light Red 25lux Active
LE Female Red 25Lux 1 Light Clear 25lux Active
LE Female Red 25Lux 1 Light Clear 25lux Inactive
LE Female Red 25Lux 1 Light Clear 200lux Active
LE Female Red 25Lux 1 Light Red 200lux Active
LE Female Red 25Lux 1 Light Red 200lux Inactive
LE Female Red 25Lux 1 Light Clear 200lux Inactive
LE Female Red 25Lux 2 Dark Red 200lux Active
LE Female Red 25Lux 2 Dark Red 25lux Active
LE Female Red 25Lux 2 Dark Red 25lux Inactive
LE Female Red 25Lux 2 Dark Red 200lux Inactive
LE Female Red 25Lux 2 Dark Clear 25lux Inactive
LE Female Red 25Lux 2 Dark Clear 25lux Active
LE Female Red 25Lux 2 Dark Clear 200lux Active
LE Female Red 25Lux 2 Dark Clear 200lux Inactive
LE Female Red 25Lux 2 Light Red 25lux Inactive
LE Female Red 25Lux 2 Light Red 25lux Active
LE Female Red 25Lux 2 Light Clear 200lux Inactive
LE Female Red 25Lux 2 Light Red 200lux Inactive
LE Female Red 25Lux 2 Light Red 200lux Active
LE Female Red 25Lux 2 Light Clear 25lux Active
LE Female Red 25Lux 2 Light Clear 200lux Active
LE Female Red 25Lux 2 Light Clear 25lux Inactive
24 LE Female Red 25Lux 3 Dark Red 200lux Active
24 LE Female Red 25Lux 3 Dark Red 200lux Inactive
24 LE Female Red 25Lux 3 Dark Red 25lux Active
24 LE Female Red 25Lux 3 Dark Red 25lux Inactive
24 LE Female Red 25Lux 3 Dark Clear 200lux Active
24 LE Female Red 25Lux 3 Dark Clear 25lux Active
24 LE Female Red 25Lux 3 Dark Clear 200lux Inactive

```
24  LE  Female  Red  25Lux  3  Dark  Clear  25lux  Inactive
24  LE  Female  Red  25Lux  3  Light  Red  25lux  Inactive
24  LE  Female  Red  25Lux  3  Light  Red  25lux  Active
24  LE  Female  Red  25Lux  3  Light  Clear  200lux  Active
24  LE  Female  Red  25Lux  3  Light  Red  200lux  Inactive
24  LE  Female  Red  25Lux  3  Light  Red  200lux  Active
24  LE  Female  Red  25Lux  3  Light  Clear  25lux  Inactive
24  LE  Female  Red  25Lux  3  Light  Clear  25lux  Active
24  LE  Female  Red  25Lux  3  Light  Clear  200lux  Inactive
;
RUN;

PROC MIXED ASYCOV NOBOUND  DATA=RatCageColorAnalyses09012023_aid ALPHA=0.05;
CLASS cage Strain Sex Color_Housing Lighting_Housing Preference_ Light_Phase
Color_Preference Lighting_Preference BehaviorCategory2;
MODEL   =  Strain Sex Color_Housing Lighting_Housing Light_Phase Color_Preference
Lighting_Preference BehaviorCategory2 Strain*Sex Strain*Color_Housing
Strain*Lighting_Housing Strain*Light_Phase Strain*Color_Preference
Strain*Lighting_Preference Strain*BehaviorCategory2 Sex*Color_Housing
Sex*Lighting_Housing Sex*Light_Phase Sex*Color_Preference Sex*Lighting_Preference
Sex*BehaviorCategory2 Color_Housing*Lighting_Housing Color_Housing*Light_Phase
Color_Housing*Color_Preference Color_Housing*Lighting_Preference
Color_Housing*BehaviorCategory2 Lighting_Housing*Light_Phase
Lighting_Housing*Color_Preference Lighting_Housing*Lighting_Preference
Lighting_Housing*BehaviorCategory2 Light_Phase*Color_Preference
Light_Phase*Lighting_Preference Light_Phase*BehaviorCategory2
Color_Preference*Lighting_Preference Color_Preference*BehaviorCategory2
Lighting_Preference*BehaviorCategory2 Strain*Sex*Color_Preference
Strain*Sex*BehaviorCategory2 Strain*Color_Housing*Lighting_Preference
Strain*Light_Phase*BehaviorCategory2 Sex*Color_Housing*Color_Preference
Sex*Color_Preference*BehaviorCategory2
Color_Housing*Lighting_Housing*Color_Preference
Color_Housing*Lighting_Housing*Lighting_Preference
Color_Housing*Lighting_Housing*BehaviorCategory2
Color_Housing*Color_Preference*Lighting_Preference
Color_Housing*Color_Preference*BehaviorCategory2
Color_Housing*Lighting_Preference*BehaviorCategory2
Lighting_Housing*Color_Preference*Lighting_Preference
Lighting_Housing*Color_Preference*BehaviorCategory2
Lighting_Housing*Lighting_Preference*BehaviorCategory2
Color_Preference*Lighting_Preference*BehaviorCategory2 Preference_
Color_Housing*Preference_ Preference_*Lighting_Housing
Preference_*Color_Preference Preference_*Lighting_Preference Preference_*Strain
Preference_*Sex/ SOLUTION DDFM=KENWARDROGER;
RANDOM cage(Sex Color_Housing Lighting_Housing ) / SOLUTION ;
RUN;
```

2. Nesting material movement data and model

```
DATA Nesting_Material_Movement_Analys; INPUT  Cage_ Pref_ Caging_Treatment &$
Lighting_Treatment &$ Sex &$ Location &$12. Sqrt_NM_Diff; Lines;
```

1 Clear 25Lux Male Clear-25lux 3.04959013639538
1 Clear 25Lux Male Clear-200lux 4.31277173056957
1 Clear 25Lux Male Red-200lux 4.25440947723653
1 Clear 25Lux Male Red-25lux 5
2 Clear 25Lux Male Red-25lux 7.79102047231298
2 Clear 25Lux Male Clear-25lux 3.08220700148449
2 Clear 25Lux Male Clear-200lux 3.27108544675923
2 Clear 25Lux Male Red-200lux 3.08220700148449
3 Clear 25Lux Male Red-200lux 3.06594194335118
3 Clear 25Lux Male Red-25lux 5.24404424085076
3 Clear 25Lux Male Clear-25lux 5.6302753041037
3 Clear 25Lux Male Clear-200lux 3.74165738677394
10 1 Clear 200Lux Female Clear-25lux 4.85798312059645
10 1 Clear 200Lux Female Clear-200lux 4.85798312059645
10 1 Clear 200Lux Female Red-200lux 2.50998007960223
10 1 Clear 200Lux Female Red-25lux 5.92452529743945
10 2 Clear 200Lux Female Red-25lux 3.68781778291716
10 2 Clear 200Lux Female Clear-25lux 3.87298334620742
10 2 Clear 200Lux Female Clear-200lux 6.6783231428256
10 2 Clear 200Lux Female Red-200lux 2.0976176963403
10 3 Clear 200Lux Female Red-200lux 2.32379000772445
10 3 Clear 200Lux Female Red-25lux 2.68328157299975
10 3 Clear 200Lux Female Clear-25lux 5.82237065120386
10 3 Clear 200Lux Female Clear-200lux 4.33589667773576
11 1 Red 200Lux Male Red-200lux 4.37035467668243
11 1 Red 200Lux Male Red-25lux 5.31977443130815
11 1 Red 200Lux Male Clear-25lux 4.83735464897913
11 1 Red 200Lux Male Clear-200lux 4.31277173056957
11 2 Red 200Lux Male Clear-25lux 4.85798312059645
11 2 Red 200Lux Male Clear-200lux 2.30217288664427
11 2 Red 200Lux Male Red-200lux 4.41588043316392
11 2 Red 200Lux Male Red-25lux 5.10881590977792
11 3 Red 200Lux Male Red-25lux 4.28952211790544
11 3 Red 200Lux Male Clear-25lux 5.03984126734166
11 3 Red 200Lux Male Clear-200lux 4.25440947723653
11 3 Red 200Lux Male Red-200lux 3.72827037646145
12 1 Red 200Lux Female Red-200lux 4.26614580154031
12 1 Red 200Lux Female Red-25lux 2.40831891575846
12 1 Red 200Lux Female Clear-25lux 4.69041575982343
12 1 Red 200Lux Female Clear-200lux 4.6690470119715
12 2 Red 200Lux Female Red-25lux 6.09097693313643
12 2 Red 200Lux Female Clear-25lux 2.44948974278318
12 2 Red 200Lux Female Clear-200lux 4.32434966208793
12 2 Red 200Lux Female Red-200lux 3.79473319220206
12 3 Red 200Lux Female Clear-25lux 3.39116499156263
12 3 Red 200Lux Female Clear-200lux 2.88097205817759
12 3 Red 200Lux Female Red-200lux 3.08220700148449
12 3 Red 200Lux Female Red-25lux 6.35609943282828
13 1 Clear 200Lux Male Clear-25lux 8.05605362444913
13 1 Clear 200Lux Male Clear-200lux 1.7606816861659
13 1 Clear 200Lux Male Red-200lux 3.78153408023781
13 1 Clear 200Lux Male Red-25lux 1.22474487139159
13 2 Clear 200Lux Male Red-25lux 0.774596669241484
13 2 Clear 200Lux Male Clear-25lux 0.948683298050513
13 2 Clear 200Lux Male Clear-200lux 8.76356092008266

13	2	Clear	200Lux	Male	Red-200lux	1.22474487139159
13	3	Clear	200Lux	Male	Red-200lux	2.0976176963403
13	3	Clear	200Lux	Male	Red-25lux	5.53172667437573
13	3	Clear	200Lux	Male	Clear-25lux	6.68580586017871
13	3	Clear	200Lux	Male	Clear-200lux	2.14476105895272
14	1	Clear	200Lux	Male	Red-200lux	3.50713558335004
14	1	Clear	200Lux	Male	Red-25lux	2.86356421265527
14	1	Clear	200Lux	Male	Clear-25lux	2.19089023002066
14	1	Clear	200Lux	Male	Clear-200lux	6.77495387438173
14	2	Clear	200Lux	Male	Clear-25lux	5.69209978830308
14	2	Clear	200Lux	Male	Clear-200lux	4.73286382647969
14	2	Clear	200Lux	Male	Red-200lux	2.66458251889485
14	2	Clear	200Lux	Male	Red-25lux	3.24037034920393
14	3	Clear	200Lux	Male	Red-25lux	4.9598387070549
14	3	Clear	200Lux	Male	Clear-25lux	2.77488738510232
14	3	Clear	200Lux	Male	Clear-200lux	5.29150262212918
14	3	Clear	200Lux	Male	Red-200lux	3.57770876399966
15	1	Clear	200Lux	Female	Red-200lux	4.31277173056957
15	1	Clear	200Lux	Female	Red-25lux	4.54972526643093
15	1	Clear	200Lux	Female	Clear-25lux	5.51361950083609
15	1	Clear	200Lux	Female	Clear-200lux	4.6690470119715
15	2	Clear	200Lux	Female	Clear-25lux	3.22490309931942
15	2	Clear	200Lux	Female	Clear-200lux	7.21803297304744
15	2	Clear	200Lux	Female	Red-200lux	2.58843582110896
15	2	Clear	200Lux	Female	Red-25lux	3.03315017762062
15	3	Clear	200Lux	Female	Red-25lux	2.44948974278318
15	3	Clear	200Lux	Female	Clear-25lux	1.70293863659264
15	3	Clear	200Lux	Female	Clear-200lux	7.81024967590665
15	3	Clear	200Lux	Female	Red-200lux	2.84604989415154
16	1	Clear	200Lux	Female	Red-25lux	5.06951674225463
16	1	Clear	200Lux	Female	Clear-25lux	5.68330889535313
16	1	Clear	200Lux	Female	Clear-200lux	4.34741302385683
16	1	Clear	200Lux	Female	Red-200lux	2.44948974278318
16	2	Clear	200Lux	Female	Red-200lux	0
16	2	Clear	200Lux	Female	Clear-25lux	5.40370243444252
16	2	Clear	200Lux	Female	Clear-200lux	2.75680975041804
16	2	Clear	200Lux	Female	Red-25lux	4.38178046004133
16	2	Clear	200Lux	Female	Red-200lux	5.3665631459995
16	2	Clear	200Lux	Female	Clear-25lux	7.40945342113708
16	2	Clear	200Lux	Female	Red-25lux	4.37035467668243
16	2	Clear	200Lux	Female	Clear-200lux	2.02484567313166
17	1	Red	200Lux	Male	Clear-25lux	4.42718872423573
17	1	Red	200Lux	Male	Clear-200lux	4.13521462562707
17	1	Red	200Lux	Male	Red-200lux	3.42052627529741
17	1	Red	200Lux	Male	Red-25lux	3.91152144312159
17	2	Red	200Lux	Male	Red-200lux	3.96232255123179
17	2	Red	200Lux	Male	Red-25lux	5.59464029227975
17	2	Red	200Lux	Male	Clear-25lux	4.13521462562707
17	2	Red	200Lux	Male	Clear-200lux	4.40454310910905
17	3	Red	200Lux	Male	Red-25lux	4.32434966208793
17	3	Red	200Lux	Male	Clear-25lux	4.9295030175465
17	3	Red	200Lux	Male	Clear-200lux	4.96990945591567
17	3	Red	200Lux	Male	Red-200lux	4.76445169982864
18	1	Red	200Lux	Male	Clear-25lux	3.37638860322683
18	1	Red	200Lux	Male	Clear-200lux	5.27257053058563

18 1 Red 200Lux Male Red-200lux 4.27784992724149
18 1 Red 200Lux Male Red-25lux 4.9598387070549
18 2 Red 200Lux Male Red-25lux 3.80788655293195
18 2 Red 200Lux Male Clear-25lux 3.87298334620742
18 2 Red 200Lux Male Clear-200lux 3.89871773792359
18 2 Red 200Lux Male Red-200lux 4.82700735445887
18 3 Red 200Lux Male Red-200lux 4.62601340248815
18 3 Red 200Lux Male Red-25lux 5.15751878329105
18 3 Red 200Lux Male Clear-25lux 4.80624593627917
18 3 Red 200Lux Male Clear-200lux 4.20713679359253
19 1 Red 200Lux Female Clear-25lux 2
19 1 Red 200Lux Female Clear-200lux 3.27108544675923
19 1 Red 200Lux Female Red-200lux 4.11096095821889
19 1 Red 200Lux Female Red-25lux 5.2820450584977
19 2 Red 200Lux Female Red-25lux 4.52769256906871
19 2 Red 200Lux Female Clear-25lux 4.53872228716409
19 2 Red 200Lux Female Clear-200lux 4.52769256906871
19 2 Red 200Lux Female Red-200lux 3.91152144312159
19 3 Red 200Lux Female Red-200lux 5.50454357780915
19 3 Red 200Lux Female Red-25lux 4.87852436706019
19 3 Red 200Lux Female Clear-25lux 2.84604989415154
19 3 Red 200Lux Female Clear-200lux 4.09878030638384
2 Clear 25Lux Male Clear-25lux 5.03984126734166
2 Clear 25Lux Male Clear-200lux 4.53872228716409
2 Clear 25Lux Male Red-200lux 4.32434966208793
2 Clear 25Lux Male Red-25lux 5.25357021462548
3 Clear 25Lux Male Red-25lux 4.69041575982343
3 Clear 25Lux Male Clear-25lux 4.75394572960189
3 Clear 25Lux Male Clear-200lux 5.45893762558247
3 Clear 25Lux Male Red-200lux 4.65832587954085
20 1 Red 200Lux Female Red-25lux 3.46410161513775
20 1 Red 200Lux Female Clear-25lux 3.72827037646145
20 1 Red 200Lux Female Clear-200lux 4.41588043316392
20 1 Red 200Lux Female Red-200lux 3.87298334620742
20 2 Red 200Lux Female Clear-25lux 3.50713558335004
20 2 Red 200Lux Female Clear-200lux 4.28952211790544
20 2 Red 200Lux Female Red-200lux 4.07430975749267
20 2 Red 200Lux Female Red-25lux 4.04969134626332
20 3 Red 200Lux Female Red-200lux 4.62601340248815
20 3 Red 200Lux Female Red-25lux 4.65832587954085
20 3 Red 200Lux Female Clear-25lux 4.53872228716409
20 3 Red 200Lux Female Clear-200lux 4.12310562561766
21 1 Clear 25Lux Male Red-200lux 4.91934955049954
21 1 Clear 25Lux Male Red-25lux 4.2190046219458
21 1 Clear 25Lux Male Clear-25lux 4.78539444560216
21 1 Clear 25Lux Male Clear-200lux 3.88587184554509
21 2 Clear 25Lux Male Clear-25lux 4.07430975749267
21 2 Clear 25Lux Male Clear-200lux 4.31277173056957
21 2 Clear 25Lux Male Red-200lux 4.61519230368573
21 2 Clear 25Lux Male Red-25lux 4.83735464897913
21 3 Clear 25Lux Male Red-25lux 4.51663591625449
21 3 Clear 25Lux Male Clear-25lux 5.07937003968012
21 3 Clear 25Lux Male Clear-200lux 4.38178046004133
21 3 Clear 25Lux Male Red-200lux 4.43846820423443
22 1 Clear 25Lux Female Red-200lux 4.70106370941726

22 1 Clear 25Lux Female Red-25lux 4.39317652729776
22 1 Clear 25Lux Female Clear-25lux 5.69209978830308
22 1 Clear 25Lux Female Clear-200lux 3.42052627529741
22 2 Clear 25Lux Female Red-25lux 5.19615242270663
22 2 Clear 25Lux Female Clear-25lux 3.86005181312376
22 2 Clear 25Lux Female Clear-200lux 3.76828873628335
22 2 Clear 25Lux Female Red-200lux 4.08656334834051
22 3 Clear 25Lux Female Clear-25lux 1.87082869338697
22 3 Clear 25Lux Female Clear-200lux 1.26491106406735
22 3 Clear 25Lux Female Red-200lux 3.57770876399966
22 3 Clear 25Lux Female Red-25lux 6.79705818718657
23 1 Red 25Lux Male Red-25lux 5.76194411635517
23 1 Red 25Lux Male Clear-25lux 3.36154726279432
23 1 Red 25Lux Male Clear-200lux 5.06951674225463
23 1 Red 25Lux Male Red-200lux 4.74341649025257
23 2 Red 25Lux Male Clear-25lux 4.93963561409139
23 2 Red 25Lux Male Clear-200lux 4.56070170039655
23 2 Red 25Lux Male Red-200lux 3.72827037646145
23 2 Red 25Lux Male Red-25lux 4.82700735445887
23 3 Red 25Lux Male Red-25lux 1
23 3 Red 25Lux Male Clear-25lux 5.40370243444252
23 3 Red 25Lux Male Clear-200lux 4.61519230368573
23 3 Red 25Lux Male Red-200lux 6.32455532033676
24 1 Red 25Lux Female Clear-25lux 4.04969134626332
24 1 Red 25Lux Female Clear-200lux 4.65832587954085
24 1 Red 25Lux Female Red-200lux 5.16720427310553
24 1 Red 25Lux Female Red-25lux 4.04969134626332
24 2 Red 25Lux Female Red-25lux 3.47850542618522
24 2 Red 25Lux Female Clear-25lux 4.81663783151692
24 2 Red 25Lux Female Clear-200lux 5.64800849857718
24 2 Red 25Lux Female Red-200lux 3.286335345031
24 3 Red 25Lux Female Red-200lux 3.3166247903554
24 3 Red 25Lux Female Red-25lux 6.73795221116921
24 3 Red 25Lux Female Clear-25lux 5.26307894677631
24 3 Red 25Lux Female Clear-200lux 2.28035085019828
1 Clear 25Lux Female Red-25lux 4.24264068711929
1 Clear 25Lux Female Clear-25lux 5.19615242270663
1 Clear 25Lux Female Clear-200lux 5.2820450584977
1 Clear 25Lux Female Red-200lux 3.286335345031
2 Clear 25Lux Female Clear-25lux 5.25357021462548
2 Clear 25Lux Female Clear-200lux 4.4497190922574
2 Clear 25Lux Female Red-200lux 3.93700393700591
2 Clear 25Lux Female Red-25lux 5.55877684387492
3 Clear 25Lux Female Red-200lux 4.58257569495584
3 Clear 25Lux Female Red-25lux 1.78885438199983
3 Clear 25Lux Female Clear-25lux 4.94974746830583
3 Clear 25Lux Female Clear-200lux 6.29285308902091
1 Clear 25Lux Female Clear-25lux 4.14728827066554
1 Clear 25Lux Female Clear-200lux 4.54972526643093
1 Clear 25Lux Female Red-200lux 4.78539444560216
1 Clear 25Lux Female Red-25lux 4.4497190922574
2 Clear 25Lux Female Red-200lux 3.97492138287036
2 Clear 25Lux Female Red-25lux 5.3103672189407
2 Clear 25Lux Female Clear-25lux 4.74341649025257
2 Clear 25Lux Female Clear-200lux 5.03984126734166

4 3 Clear 25Lux Female Clear-25lux 4.60434577328854
4 3 Clear 25Lux Female Clear-200lux 5.23450093132096
4 3 Clear 25Lux Female Red-200lux 4.78539444560216
4 3 Clear 25Lux Female Red-25lux 4.83735464897913
1 Red 25Lux Male Red-25lux 4.52769256906871
1 Red 25Lux Male Clear-25lux 4.76445169982864
1 Red 25Lux Male Clear-200lux 2.44948974278318
1 Red 25Lux Male Red-200lux 5.33853912601566
2 Red 25Lux Male Clear-25lux 5.42217668469038
2 Red 25Lux Male Clear-200lux 5.37587202228625
2 Red 25Lux Male Red-200lux 3.22490309931942
5 2 Red 25Lux Male Red-25lux 4.69041575982343
5 3 Red 25Lux Male Red-200lux 5.42217668469038
5 3 Red 25Lux Male Red-25lux 3.03315017762062
5 3 Red 25Lux Male Clear-25lux 4.43846820423443
5 3 Red 25Lux Male Clear-200lux 4.17133072292284
1 Red 25Lux Male Red-200lux 3.87298334620742
1 Red 25Lux Male Red-25lux 4.9598387070549
1 Red 25Lux Male Clear-25lux 5.11859355682789
1 Red 25Lux Male Clear-200lux 4.28952211790544
2 Red 25Lux Male Red-25lux 4.38178046004133
2 Red 25Lux Male Clear-25lux 4.93963561409139
2 Red 25Lux Male Clear-200lux 4.76445169982864
6 2 Red 25Lux Male Red-200lux 4.43846820423443
6 3 Red 25Lux Male Clear-25lux 4.98998997994986
6 3 Red 25Lux Male Clear-200lux 4.26614580154031
6 3 Red 25Lux Male Red-200lux 5.07937003968012
6 3 Red 25Lux Male Red-25lux 4.7116875957559
2 Red 25Lux Female Red-200lux 4.07430975749267
2 Red 25Lux Female Red-25lux 4.2190046219458
2 Red 25Lux Female Clear-25lux 5.2820450584977
2 Red 25Lux Female Clear-200lux 4.06201920231798
3 Red 25Lux Female Red-25lux 5.84807660688538
3 Red 25Lux Female Clear-25lux 4.9598387070549
3 Red 25Lux Female Clear-200lux 5.51361950083609
7 3 Red 25Lux Female Red-200lux 3.16227766016838
1 Red 25Lux Female Red-200lux 4.76445169982864
1 Red 25Lux Female Red-25lux 3.24037034920393
1 Red 25Lux Female Clear-25lux 4.81663783151692
1 Red 25Lux Female Clear-200lux 3.61939221417077
2 Red 25Lux Female Red-25lux 5.16720427310553
2 Red 25Lux Female Clear-25lux 4.17133072292284
2 Red 25Lux Female Clear-200lux 5.37587202228625
8 2 Red 25Lux Female Red-200lux 4.34741302385683
8 3 Red 25Lux Female Clear-25lux 4.35889894354067
8 3 Red 25Lux Female Clear-200lux 4.06201920231798
8 3 Red 25Lux Female Red-200lux 3.14642654451046
8 3 Red 25Lux Female Red-25lux 5.22494019104525
1 Clear 200Lux Male Red-200lux 6.94262198308391
1 Clear 200Lux Male Red-25lux 1.73205080756888
1 Clear 200Lux Male Clear-25lux 3.30151480384384
1 Clear 200Lux Male Clear-200lux 4.23083916026124
2 Clear 200Lux Male Clear-25lux 5.96657355607052
2 Clear 200Lux Male Clear-200lux 2.73861278752583
2 Clear 200Lux Male Red-200lux 1.8165902124585

```
9  2  Clear  200Lux  Male  Red-25lux  5.91607978309962
9  3  Clear  200Lux  Male  Red-25lux  4.32434966208793
9  3  Clear  200Lux  Male  Clear-25lux  4.47213595499958
9  3  Clear  200Lux  Male  Clear-200lux  4.49444101084885
9  3  Clear  200Lux  Male  Red-200lux  4.58257569495584
;
RUN;

PROC MIXED ASYCOV NOBOUND  DATA=Nesting_Material_Movement_Analys ALPHA=0.05;
CLASS Cage_ Caging_Treatment Lighting_Treatment Sex Location;
MODEL  Sqrt_NM_Diff =  Cage_(Caging_Treatment Lighting_Treatment Sex ) Location
Sex Caging_Treatment Lighting_Treatment Caging_Treatment*Lighting_Treatment
Caging_Treatment*Sex Caging_Treatment*Location Lighting_Treatment*Sex
Lighting_Treatment*Location Sex*Location Caging_Treatment*Lighting_Treatment*Sex
Caging_Treatment*Lighting_Treatment*Location Pref_/ SOLUTION DDFM=KENWARDROGER;
RANDOM Caging_Treatment Lighting_Treatment Sex / SOLUTION ;
RUN;
```
