# Supplementary figures and images for: The Future Is Not Bright: Evaluation of Rat Preferences for Color and Intensity of Light
Source: Animals (Basel). 2024 Jul 12;14(14):2045. doi: 10.3390/ani14142045 (PMC11273897; doi:10.3390/ani14142045)

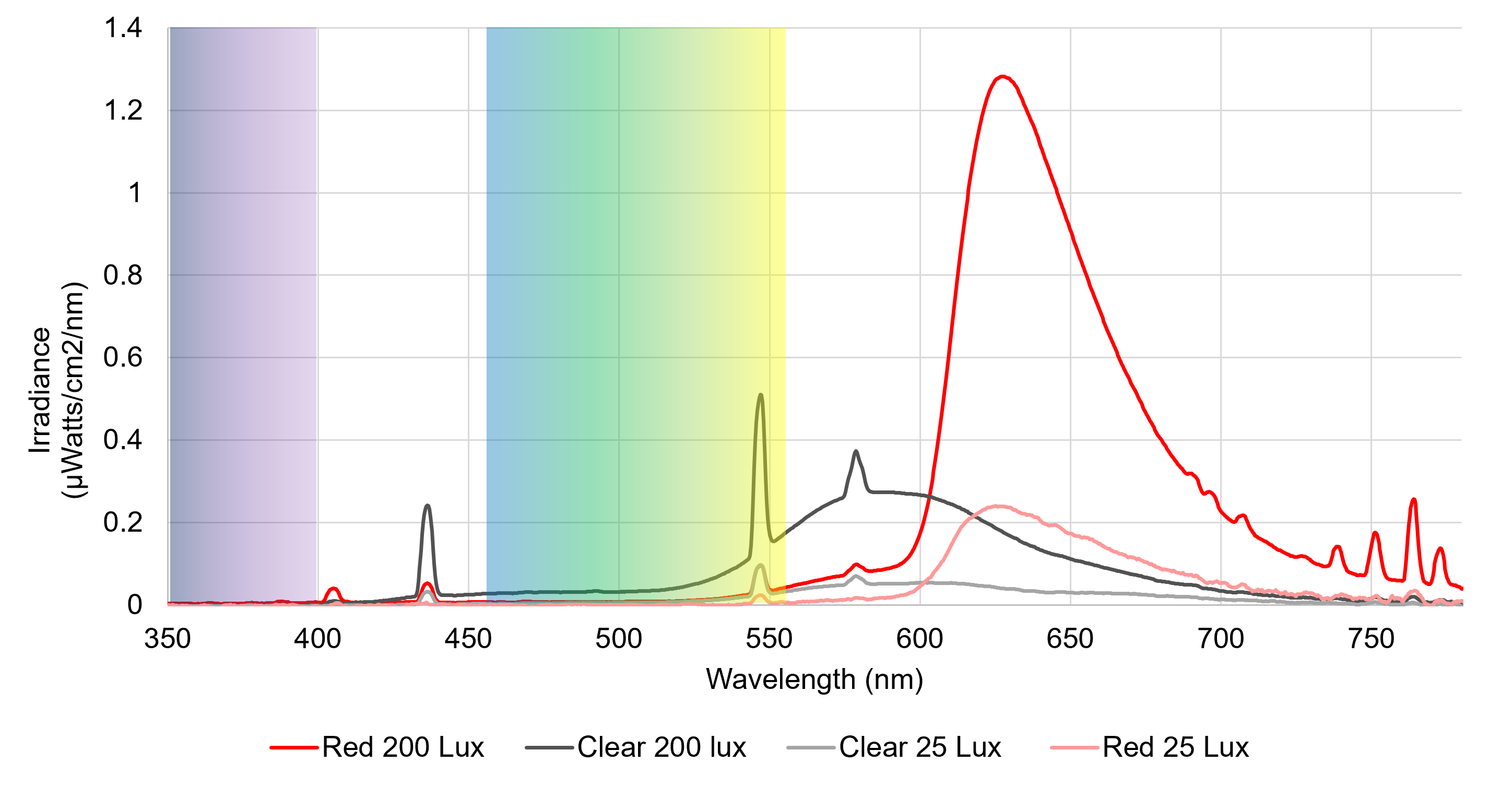

Supplement: Supplementary file 1 [file animals-14-02045-s001.zip › Figure S1 - Spectrograph of the four lighting conditions.png]
